# Modeling the Effects of Single Nucleotide Polymorphisms (SNPs) on the Structure and Function of the Human *RET* Gene: An In Silico Study

**DOI:** 10.1155/humu/8848146

**Published:** 2026-06-27

**Authors:** Nabilah Anzoom, Md. Arju Hossain, Md. Tanvir Hossain, Md. Moin Uddin, Mahfuj Khan, Md. Shofiqul Islam, Siddique Akber Ansari, Md. Habibur Rahman

**Affiliations:** ^1^ Department of Computer Science and Engineering, Islamic University, Kushtia, Bangladesh, iu.ac.bd; ^2^ Department of BUBT Research Graduate School, Bangladesh University of Business and Technology, Dhaka, Bangladesh, bubt.edu.bd; ^3^ Department of Biochemistry and Biotechnology, Khwaja Yunus Ali University, Sirajganj, Bangladesh, kyau.edu.bd; ^4^ Department of Biotechnology, Bangladesh Agricultural University, Mymensingh, Bangladesh, bau.edu.bd; ^5^ Institute for Intelligent Systems Research and Innovation (IISRI), Deakin University, Geelong, Victoria, Australia, deakin.edu.au; ^6^ Department of Pharmaceutical Chemistry, College of Pharmacy, King Saud University, Riyadh, Saudi Arabia, ksu.edu.sa; ^7^ Center for Advanced Bioinformatics and Artificial Intelligence Research, Islamic University, Kushtia, Bangladesh, iu.ac.bd

**Keywords:** entrectinib, M918T, MEN2, missense, nonsynonymous and polymorphism, *RET*

## Abstract

The *RET* proto‐oncogene plays a critical role in multiple cancers and developmental disorders, where nonsynonymous single nucleotide polymorphisms (nsSNPs) may alter protein stability, function, and therapeutic response. In this study, a total of 28,335 SNPs from the NCBI dbSNP database, including 2377 nsSNPs, were systematically analyzed using 10 in silico prediction tools. Among these 33 high‐risk variants were identified with 28 predicted to destabilize *RET* protein. Further structural and functional analyses highlighted 11 potentially pathogenic nsSNPs (R721G, A756D, Y791C, E734K, E805K, F893L, R897Q, R897G, R912Q, R912G, and M918T), predominantly clustered within the tyrosine kinase domain, suggesting functional hotspots may contribute disease susceptibility. Molecular docking of eight *RET* inhibitors revealed mutation‐specific differences in drug binding affinity. Among the tested compounds, entrectinib exhibited consistently strong binding affinities across all variants (−9.7 to −10.7 kcal/mol), whereas reduced binding affinities were observed for several inhibitors against E734K, A756D, and R897G variants. Molecular dynamics simulations further supported these findings, showing that A756D, E734K, and R897Q variants maintained comparatively stable conformations, whereas R897G and Y791C exhibited increased structural flexibility and destabilization. Survival analyses revealed that RET dysregulation correlates with poor prognosis in thyroid cancer, lung carcinoma, breast cancer, and sarcoma. Clinical databases reported that p.Met918Thr (pathogenic) and p.Arg897Gln (risk factor) emerged as significant variants linked to MEN2‐related cancers and Hirschsprung disease. As a conclusion, this integrative in silico study identifies deleterious *RET* nsSNPs with potential structural, functional, and therapeutic significance providing mechanisms for precision oncology and future clinical investigation.

**Highlights**

• Multiple Endocrine Neoplasia Type 2 (MEN2) is one of the disorders linked to *RET* gene mutations, which are linked to several cancers and developmental issues.
• Thirty‐three (33) of the 2377 nonsynonymous single nucleotide polymorphisms (nsSNPs) in the RET gene were determined to be harmful; 11 of them were discovered to be in highly conserved areas and eight variants may be carcinogenic.
• MEM2A and MEN2B were found to be directly linked to the M918T nsSNP via clinical database validation.
• RET gene variations are potentially linked to survival outcomes in several cancers, highlighting their role in personalized medicine and drug development.

## 1. Introduction

*RET* is a gene that has been associated with multiple cancers in humans due to rearrangements and point mutations, serving as a key example of how a single gene can responsible for diverse tumors and developmental disorders [1, 2]. These mutations typically enhance RET activity and are found in cancers including lung adenocarcinomas, medullary and papillary thyroid carcinomas, and myeloproliferative disorders [3]. MEN2 is a hereditary cancer syndrome caused by germline mutations in the RET proto‐oncogene, leading to constitutive activation of its tyrosine kinase domain. It is characterized by medullary thyroid carcinoma (MTC), pheochromocytoma, and parathyroid disease, with subtype variations (MEN2A, MEN2B, and FMTC). Early genetic screening enables timely interventions, including prophylactic thyroidectomy, significantly improving patient outcomes [4–7]. Additionally, RET is essential in a subset of ER^+^ (estrogen receptor‐positive) breast tumors, where its expression is increased [8]. ER, is a pivotal transcription factor in breast cancer, modulates the expression of the RET and GFRA1 genes, which collaboratively promote estrogen‐induced cellular proliferation [9]. Head and neck squamous cell carcinoma (HNSC) and breast invasive carcinoma (BRCA) both exhibits significantly higher RET expression compared to normal tissues. Moreover, higher expression and mutation rates of the RET gene are associated with negative outcomes in patients, particularly those diagnosed with sarcomas [10].

Different RET proto‐oncogene polymorphisms are associated with different MEN2 onset and severity rates. Found on the long arm of Chromosome 10 (10q11), the RET gene consists of 21 exons [11]; and either 18 or 5 introns [12, 13], with a molecular mass of 170 kilodaltons [14]. The cytoplasmic tyrosine kinase domain, transmembrane domain, and extracellular ligand‐binding domain are the three functional domains that make up the RET protein [15]. RET dimerizes and generates heterohexameric complexes from GDNF‐family ligands, coreceptors, and alpha receptors of the GDNF family (GFR*α*1 to GFR*α*4). This intricate activation leads to transcellular kinase activation and ensuing signaling [16–19]. Wells and Santoro [15] and Donis‐Keller et al. [5] identified seven RET gene mutations in Exons 7 and 8, associated with MEN2A and FMTC. All mutations impacted cysteine‐coding codons such as 6 in Exon 7 and 1 in Exon 8 [20]. The phenotype linked to RET gene mutations in MTC is influenced by disease severity, coexisting endocrine tumors like hyperparathyroidism or pheochromocytoma, and age of onset. Although mutations at codons 609, 611, 618, and 620 explain 10%–15% of cases, codon 634 mutations in Exon 11 are found in approximately 85% of MEN2 patients [21].

The differences in DNA within genes are referred to as single nucleotide polymorphisms (SNPs), although their prevalence differs by sequence. They occur every 100 to 300 base pairs on average and, remarkably, constitute nearly 90% of all human genetic variations [22]. In human genomes, SNPs affect splicing, gene expression, and the binding of transcription factors that are both coding and noncoding. Although certain SNPs do not affect cells, others can change a person′s sensitivity to illnesses or how they react physiologically to drugs [23, 24]. Because they can change amino acid residues, nsSNPs are important among SNPs in comparison to other proteins [25]. Although some nsSNPs influence structural traits, others have functional implications [26]. Alterations in the characteristics of a protein can either enhance or detrimentally affect its folding and functionality. These modifications may disrupt gene regulation [27, 28], destabilize protein structure, modify protein hydrophobicity, charge, and geometry [29, 30], and affect protein conformation, all of which are unwanted side effects [31]. As a consequence of this, there is a predominant association between nsSNPs and a number of human disorders. Over the course of the past several years, this study has become more diverse because of the impact that nsSNPs have on human health and disease conditions.

Recent developments in genotyping techniques have facilitated multiple investigations that have identified several hundred SNPs in the coding region of the RET gene. Further research is inevitable to identify which RET gene SNPs are most associated with disease risk. Several different methodologies, such as NCBI dbSNP, UniProtKB, SIFT, SNAP2, Predict‐SNP, SNPs&GO, PANTHER, PhD‐SNP, PolyPhen, GPS‐MSP, NetPhos, Mutpred2, MUpro, I‐Mutant, Mutation 3D, Project HOPE, EnrichR, STRING, BioGRID, Autodock Vina, AlphaFold, Phyre2, and Kaplan‐Meier Plotter, have been utilized in a multitude of studies in order to evaluate the significant implications of the most deleterious nsSNPs that have been also investigated in the RET gene according to Hossen et al., (2025) [32] studies. Furthermore, we gave prospective views that were able to provide substantial information regarding the potential consequences that these factors could have on the structure, activity, and stability of the protein. Overall, this study enhances the understanding of the RET gene, offering insights into disease mechanisms and supporting the development of targeted therapies for better clinical results.

## 2. Materials and Methods

### 2.1. Data Mining

The mutant information of RET gene was obtained from the NCBI dbSNP database (https://www.ncbi.nlm.nih.gov/snp/: accessed on 06 March 2026) [33]. Then the amino acid sequence was collected from UniProt (UniProt ID: P07949.3) database (https://www.uniprot.org; accessed on 06 March 2026) [34].

### 2.2. Estimation of the Deleterious nsSNPs

To evaluate the potential impacts of genetic diversity derived from the dbSNP datasets, our study used 10 different bioinformatics tools, each intended for a particular purpose. Among these seven tools including SIFT, PhD‐SNP, Predict‐SNP, SNAP2, PANTHER, PolyPhen‐1, and PolyPhen‐2, were utilized in the integrated Predict‐SNP (https://loschmidt.chemi.muni.cz/predictsnp1/; accessed on 10 March 2026) database. Predict‐SNP furnished data for each mutation and demonstrated enhanced predictive efficacy, underscoring the precision and reliability of consensus predictions compared to those from individual tools [35]. Another three tools, including PROVEAN, SNP&GO and FATHMM were utilized in their separate databases for rechecking our results. SNPs&GO (https://snps-and-go.biocomp.unibo.it/snps-and-go/; accessed on 10 March 2026) forecasts deleterious single amino acid polymorphisms (SAPs), providing an assessment of their potential correlation with human diseases for each protein variant. A likelihood value beyond 0.5 implies disease‐associated mutations, whereas a score of 0.5 denotes no meaningful effect [36]. With exceptional results in benchmark evaluations, the FATHMM server (https://fathmm.biocompute.org.uk/inherited.html; accessed on 10 March 2026) predicts harmful point mutations in the human genome, particularly in noncoding regions where the likelihood of identifying severe pathogenic mutations is higher [37]. PROVEAN (http://provean.jcvi.org; accessed on 10 March 2026). predicts the potential deleterious impact of amino acid substitutions and indels by calculating sequence homology‐based scores. Variants with scores below the default cutoff (≤ −2.5) were classified as “deleterious,” whereas those above were considered “neutral.” [38].

### 2.3. Protein Stability Analysis

For the purpose of determining the durability of proteins, we utilized the SVM‐based service known as I‐Mutant 2.0 (https://folding.biofold.org/cgi-bin/i-mutant2.0.cgi; accessed on 16 March 2026). This approach was crucial in our research as it predicted changes in the stability of proteins resulting from mutations. The input parameters included the RET sequence, its variants, and a specified temperature of 25°C and pH of 7. The accuracy index ranged from 0 to 10, with higher values denoting greater dependability [39]. MUpro (https://mupro.proteomics.ics.uci.edu/; accessed on 17 March 2026), compares wild‐type and mutant‐type residues to determine changes in the protein sequence. A number below zero implies that the mutation adversely affects protein functionality, whereas a value above zero signifies enhanced protein stability [40].

### 2.4. Evaluation of Cancer‐Associated nsSNPs

Mutation3D (http://www.mutation3d.org/; accessed on 17 March 2026) is used to identify clusters of amino acid substitutions resulting from somatic cancer mutations. It plays a crucial role in analyzing the spatial distribution of these alterations within protein structures and models. It applies a structural clustering approach to detect potential cancer‐driving mutations by mapping variants onto protein models and evaluating their spatial distribution [41].

### 2.5. Identification of Functional and Structural Modifications of *RET*

The pathogenic potential of amino acid substitutions and their effects on protein structure and function were assessed using the Mutpred2 tool (http://mutpred.mutdb.org/; accessed on 18 March 2026). This tool applies machine‐learning approaches to predict the functional impact of missense variants based on sequence, structural, and evolutionary features. Furthermore, it provides molecular mechanisms and disease‐related biological pathways [42].

### 2.6. Identification of the Domains of *RET*

The domain organization of the RET protein was analyzed using the InterPro database (https://www.ebi.ac.uk/interpro/; accessed on 18 March 2026). InterPro integrates multiple protein signature databases to classify proteins into families and predict functional domains [43]. Based on this analysis, RET was classified into distinct protein families, and its conserved functional domains were identified, highlighting key regions responsible for its biological activity.

### 2.7. Posttranslational Modification (PTM) Site Prediction

Identifying PTM sites is essential, as these alterations profoundly influence protein stability, and enzyme activity, functional regulation and facilitating the comprehension of pathogenicity associated with genetic differences [44]. Phosphorylation sites (serine, threonine, and tyrosine) were predicted using the NetPhos 3.1 (https://services.healthtech.dtu.dk/services/NetPhos-3.1/; accessed on 19 March 2026) [45], which employs neural network‐based algorithms. Potential methylation sites were identified using GPS‐MSP 1.0 (https://msp.biocuckoo.org/; accessed on 19 March 2026) [46]. Additionally, ubiquitination sites were predicted using the UbPred online tool (https://gpsuber.biocuckoo.cn/online.php; accessed on 19 March 2026), which identifies lysine residues with high probability of ubiquitination based on sequence‐derived features [47].

### 2.8. Prediction of Protein–Protein Interaction (PPI)

PPIs associated with *RET* were predicted using multiple publicly available databases, including STRING (https://string-db.org/; accessed on 19 March 2026) [48], BioGRID (https://thebiogrid.org; accessed on 08 April 2026) [49], and IntAct (https://www.ebi.ac.uk/intact/; accessed on 08 April 2026) [50]. These databases integrate experimentally validated interactions, computational predictions, and literature‐derived evidence to generate comprehensive interaction networks across multiple species. Both direct (physical) and indirect (functional) interactions were considered to construct the *RET* interaction network. STRING provides a confidence‐based interaction framework combining diverse evidence sources, whereas BioGRID and IntAct primarily curate experimentally validated molecular interactions, thereby enhancing the reliability of the predicted network. PPI analysis is essential, as mutations in *RET* may disrupt normal interaction patterns, thereby affecting key signaling pathways and contributing to disease progression. This integrative approach facilitates a deeper understanding of disease mechanisms and supports the identification of potential therapeutic targets.

### 2.9. Prediction of the Alterations of Protein 3D Structure Upon Mutation

The protein‐coding sections of the human genome have recently been discovered to have many point mutations. Comprehending the effects of these modifications on the protein′s three‐dimensional conformation is essential for developing innovative diagnostics and therapies, as well as for elucidating the protein′s functionality [51]. The structural as well as biological investigation of point mutations within a sequence can be investigated with Project HOPE (https://www3.cmbi.umcn.nl/hope/input/; accessed on 26 March 2026). It provides an exhaustive examination of amino acid modifications and evaluates the structural and functional consequences on proteins after mutation. Conserved areas and domains of functional importance are the focus of our current studies [52].

### 2.10. Gene Ontology (GO) Analysis

GO was founded on the principle of methodically connecting a group of genes to a term that is under the purview of functional biology [53]. To identify significant (*p* < 0.05) correlations within the input gene set and carefully selected databases that span biological processes (BPs), cellular components (CCs), and molecular activities. Enrichment analysis was conducted using the EnrichR platform (https://maayanlab.cloud/enrichr-kg; accessed on 19 March 2026) [54]. The enriched GO results were subsequently visualized using the SRplot (https://www.bioinformatics.com.cn/e; accessed on 19 March 2026) server, enabling clear graphical representation of the analysis outcomes [55].

### 2.11. Validation of Potential RET Gene Variants

#### 2.11.1. Molecular Docking

Molecular docking analysis was carried out with AutoDock Vina [56] to assess the influence of deleterious point mutations on RET binding affinity. Based on MutPred2 scoring, the pool of mutated proteins was filtered from 11 to 7. Using Phyre2 (https://www.sbg.bio.ic.ac.uk/phyre2/html/page.cgi?id=index; accessed on 20 March 2026) [57] and AlphaFold server (https://alphafoldserver.com/; accessed on 20 March 2026) [58], we modeled target protein structure derived from the crystal structure of the RET, along with eight specific RET mutations: E734K, A756D, Y791C, E805K, F893L, R897Q, R897G, and M918T. Four compounds such as dabrafenib (CID: 44462760), entrectinib (CID: 25141092), larotrectinib (CID: 46188928), and sorafenib (CID: 216239) were selected after reviewing literature for docking studies [59, 60]. The PDB of target proteins and ligands were converted to the PDBQT format utilizing AutoDock Vina. To ensure maximum coverage, the grid box dimensions were expanded to their maximum coordinates *x* = −12.50, *y* = 2.917, and *z* = −12.667, respectively. BIOVIA Discovery Studio Visualizer V21 [61] was utilized to visualize the docking outcomes and explore the binding interactions between the ligands and receptor proteins.

#### 2.11.2. Survival Analysis

An effective instrument for accurately anticipating the effects of medicinal medicines and forecasting their influence on cancer survival rates is the Kaplan‐Meier plotter (https://kmplot.com/analysis/; accessed on 21 March 2026). Comprehensive meta‐analyses allow for the detection and evaluation of cancer biomarkers. Estimating the time before death, which is an inevitable event for everyone, can help guide important clinical decisions, alter healthcare policy, and allocate resources effectively [62].

#### 2.11.3. Molecular Dynamics Simulation Studies

A 100 ns molecular dynamics simulation was conducted to evaluate the binding stability of seven mutants including E734K, A756D, Y791C, F893L, R897Q, M918T, and R897G and wild‐type RET protein against only entrectinib ligand based on molecular docking binding score analysis. Molecular dynamics simulations were performed on a Linux operating system using Maestro 2020.4 Schrodinger (LLC, New York, New York, 2020) with the OPLS 2005 force field to assess the stability of various protein‐ligand complexes [63]. The TIP3P water model was used to create a predefined orthorhombic periodic boundary box, and 0.15 M Na^+^ and Cl^−^ were added to neutralize the system. Energy minimization was performed using a step‐based steepest descent algorithm until convergence was achieved based on the predefined maximum force threshold. Following minimization, the system was subjected to a 100 ps equilibration phase under controlled temperature and pressure conditions to stabilize the system prior to production simulation. The SHAKE algorithm was applied to constrain hydrogen bonds, and the system was heated to 310 K [64]. Subsequently, a 100 ns production MD simulation was carried out, and trajectory snapshots were recorded at 100 ps intervals. The stability of the protein‐ligand complex was assessed by analyzing root mean square deviation (RMSD), root mean square fluctuation (RMSF), radius of gyration (Rg) and solvent‐accessible surface area (SASA) analysis [65, 66].

#### 2.11.4. Validation Using Clinical and Cancer Databases

To validate the predicted deleterious nsSNPs in the RET gene, selected variants were cross‐referenced with curated databases, including ClinVar, the Leiden Open Variation Database (LOVD), and the Clinical Knowledgebase (CKB). Information on clinical significance, associated phenotypes, and SNP IDs was obtained from ClinVar (https://www.ncbi.nlm.nih.gov/clinvar/; accessed on 10 April 2026) [67], whereas LOVD (https://www.lovd.nl/; accessed on 10 April 2026) was used for additional confirmation of variant pathogenicity [68]. The CKB (https://ckb.jax.org/; accessed on 10 April 2026) database was employed to identify functional annotations, including gain‐ or loss‐of‐function effects and oncogenic relevance [69]. Variants were further mapped to their exon locations and protein domains, with emphasis on the RET kinase domain. This integrative approach ensured reliable validation and supported the selection of high‐impact variants for downstream analyses.

## 3. Results

### 3.1. Data Mining

**Figure 1:**
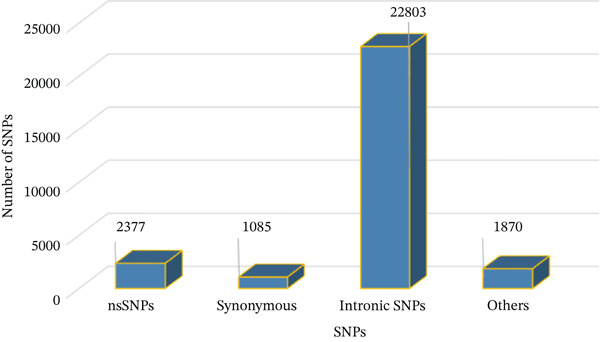
Distribution of SNPs in the RET gene using dbSNP database. The majority of variants are intronic SNPs (22,803), followed by nonsynonymous SNPs (2377), others (1870), and synonymous SNPs (1085). This chart represents a predominance of noncoding variants, with a notable proportion of coding SNPs that may influence RET gene function and expression.

Data on RET gene polymorphisms was sourced from the NCBI dbSNP database. There were 28,335 SNPs found in all, with 1085 synonymous variants, 22,803 intron region variants, 2,377 missense variants, and other types of variants making up the remaining portion (Accessed time: 25 March 2026). The 3D bar chart (Figure 1) illustrates the distribution of different types of SNPs identified in the RET gene. Intronic SNPs are the most abundant (22,803), suggesting a high frequency of noncoding variants that may influence gene regulation or splicing. Nonsynonymous SNPs (2377) can alter the amino acid sequence and potentially impact protein function, whereas synonymous SNPs (1085) do not change the protein sequence but may affect translation efficiency or splicing. This distribution highlights the dominance of intronic variations and the presence of functionally relevant coding SNPs in the RET gene. We removed the remaining SNPs and focused exclusively on 2377 nonsynonymous SNPs for our analysis.

### 3.2. Estimation of the Deleterious nsSNPs

We found harmful nsSNPs that could alter the RET protein′s function or structure by applying 10 distinct deleterious SNP prediction algorithms. These algorithms are as follows: SIFT, PROVEAN, Predict‐SNP, SNAP2, PolyPhen‐1, SNP&GO, PhD‐SNP, PANTHER, PolyPhen‐2, and FATHMM. For analysis, 2377 polymorphic inputs in all were taken from the dataset. According to all 10 in silico techniques, 33 out of 2377 nsSNPs were harmful (Table 1).

**Table 1:** High‐risk 33 nsSNPs identified by 10 bioinformatics tools.

SNP (rs) id	AA change	SIFT	Predict‐SNP	PROVEAN	SNAP2	PolyPhen‐1	SNP&GO	PhD‐SNP	PANTHER	PolyPhen‐2	FATHMM
rs74799832	M918T	DL	DL	D	E	PD	D	D	PD	PD	D
rs76087194	R897Q	DL	DL	D	E	PD	D	D	PD	PD	D
rs1060500759	R897G	DL	DL	D	E	PD	D	D	PD	PD	D
rs77503355	C620Y	DL	DL	D	E	PD	D	D	PD	PD	D
rs79890926	C620W	DL	DL	D	E	PD	D	D	PD	PD	D
rs77724903	Y791C	DL	DL	D	E	PD	D	D	PD	PD	D
rs78347871	R912Q	DL	DL	D	E	PD	D	D	PD	PD	D
rs1838227061	R912G	DL	DL	D	E	PD	D	D	PD	PD	D
rs142318626	R817S	DL	DL	D	E	PD	D	D	PD	PD	D
rs201487882	G885R	DL	DL	D	E	PD	D	D	PD	PD	D
rs377767432	S922Y	DL	DL	D	E	PD	D	D	PD	PD	D
rs768188546	F893L	DL	DL	D	E	PD	D	D	PD	PD	D
rs779996040	R813W	DL	DL	D	E	PD	D	D	PD	PD	D
rs1554819146	R721G	DL	DL	D	E	PD	D	D	PD	PD	D
rs1588875462	P720H	DL	DL	D	E	PD	D	D	PD	PD	D
rs1588880114	P996R	DL	DL	D	E	PD	D	D	PD	PD	D
rs1838077120	E734K	DL	DL	D	E	PD	D	D	PD	PD	D
rs1838079858	A756D	DL	DL	D	E	PD	D	D	PD	PD	D
rs138010639	Y1062N	DL	DL	D	E	PD	D	D	PD	PD	D
rs80069458	C611Y	DL	DL	D	E	PD	D	D	PD	PD	D
rs112448213	C243S	DL	DL	D	E	PD	D	D	PD	PD	D
rs121913306	A883F	DL	DL	D	E	PD	D	D	PD	PD	D
rs144801580	G183D	DL	DL	D	E	PD	D	D	PD	PD	D
rs149148794	L790S	DL	DL	D	E	PD	D	D	PD	PD	D
rs150261092	N151T	DL	DL	D	E	PD	D	D	PD	PD	D
rs377767396	C609W	DL	DL	D	E	PD	D	D	PD	PD	D
rs377767400	C618W	DL	DL	D	E	PD	D	D	PD	PD	D
rs377767416	Q781P	DL	DL	D	E	PD	D	D	PD	PD	D
rs377767418	E805K	DL	DL	D	E	PD	D	D	PD	PD	D
rs587778657	S811Y	DL	DL	D	E	PD	D	D	PD	PD	D
rs536486113	L1061H	DL	DL	D	E	PD	D	D	PD	PD	D
rs587778659	Y1062S	DL	DL	D	E	PD	D	D	PD	PD	D
rs755369964	C585W	DL	DL	D	E	PD	D	D	PD	PD	D

Abbreviations: AA stands for amino acid; D for disease; DL for detrimental; E for impact; and PD for likely damage.

### 3.3. Protein Stability Analysis

I‐Mutant 2.0, as well as MUpro, were the two additional approaches that were utilized to reevaluate 33 nsSNPs that had been previously defined as harmful. This was done to investigate the stability of proteins. For the protein, 28 nsSNPs were responsible for its destabilization, whereas only 5 (S922Y, C611Y, A883F, L790S, and C618W) were responsible for its stabilization, as stated by MUpro. As part of our analysis, we focused on 28 nsSNPs for further analysis that were predicted to exhibit “decrease” stability by both servers (Table 2).

**Table 2:** Predicted RET protein stability changes in MUpro and I‐mutant 2.0.

SNP (rs) id	AA change	I‐Mutant 2.0	RI	MUpro	DDG Value Prediction
rs74799832	M918T	Decrease	7	Decrease	−1.2599304
rs76087194	R897Q	Decrease	9	Decrease	−1.2662051
rs1060500759	R897G	Decrease	8	Decrease	−1.7482604
rs77503355	C620Y	Decrease	0	Decrease	−0.57749087
rs79890926	C620W	Decrease	5	Decrease	−0.84054886
rs77724903	Y791C	Decrease	2	Decrease	−0.35915796
rs78347871	R912Q	Decrease	9	Decrease	−0.842332
rs1838227061	R912G	Decrease	8	Decrease	−1.5041949
rs142318626	R817S	Decrease	7	Decrease	−0.40018634
rs201487882	G885R	Decrease	8	Decrease	−0.60653478
rs377767432	S922Y	Increase	1	Decrease	−0.65287457
rs768188546	F893L	Decrease	7	Decrease	−1.2392436
rs779996040	R813W	Decrease	7	Decrease	−0.62088032
rs1554819146	R721G	Decrease	8	Decrease	−1.5609221
rs1588875462	P720H	Decrease	9	Decrease	−1.1158621
rs1588880114	P996R	Decrease	5	Decrease	−0.8229215
rs1838077120	E734K	Decrease	6	Decrease	−0.88158634
rs1838079858	A756D	Decrease	6	Decrease	−0.64553137
rs138010639	Y1062N	Decrease	4	Decrease	−1.1722596
rs80069458	C611Y	Increase	3	Decrease	−1.0407691
rs112448213	C243S	Decrease	7	Decrease	−1.428069
rs121913306	A883F	Increase	3	Decrease	−0.084154533
rs144801580	G183D	Decrease	8	Decrease	−0.37249931
rs149148794	L790S	Increase	3	Decrease	−1.753337
rs150261092	N151T	Decrease	6	Decrease	−1.4453598
rs377767396	C609W	Decrease	3	Decrease	−0.65990973
rs377767400	C618W	Increase	0	Decrease	−1.189269
rs377767416	Q781P	Decrease	7	Decrease	−1.1793584
rs377767418	E805K	Decrease	8	Decrease	−1.1302396
rs587778657	S811Y	Decrease	1	Decrease	−0.86906937
rs536486113	L1061H	Decrease	8	Decrease	−1.9683244
rs587778659	Y1062S	Decrease	3	Decrease	−1.2863102
rs755369964	C585W	Decrease	6	Decrease	−0.9244615

### 3.4. Evaluation of Cancer‐Associated nsSNPs

**Figure 2:**
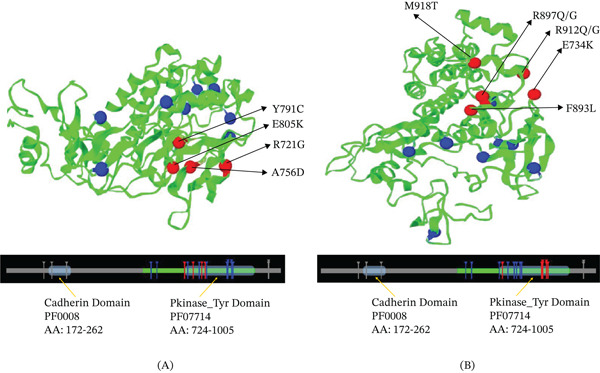
By using Mutation3D server, structural visualization of spatial clustering of cancer‐associated nsSNPs in RET gene was generated. Figure (A) represents Cluster‐1, which contains four nsSNPs (R721G, A756D, Y791C and E805K); and (B) represents the Cluster‐2 includes seven nsSNPs (E734K, F893L, R897Q, R897G, R912Q, R912G, and M918T) linked to cancer. Red marks indicate nsSNPs that the Mutation 3D service has recognized as potentially carcinogenic. Red is used to show clustered mutations whereas blue is used to identify covered mutations.

The study identified 11 out of 28 nsSNPs that are significantly associated with an increased risk of cancer, as determined using the Mutation 3D server. These 11 nsSNPs include R721G, A756D, Y791C, E734K, E805K, F893L, R897Q, R897G, R912Q, R912G, and M918T represent clustered mutations, as visually highlighted in red color in Figure 2. Except R721G and E734K, these nine mutations suggest potential functional hotspots within the protein structure in the tyrosine kinase domain that may critically influence oncogenic pathways. Further investigation of these 11 nsSNPs is warranted to elucidate their mechanistic roles in cancer development, including their effects on protein stability, interactions, and signaling pathways. The findings underscore the importance of these genetic variants in cancer susceptibility and highlight their potential as targets for diagnostic or therapeutic strategies.

### 3.5. Identification of Functional and Structural Modifications of *RET*

The MutPred2 tool was utilized in order to conduct additional research on 11 nsSNPs that were found to have the potential to cause harm. Table S1 (see Supporting Information file) compiles the outcomes, encompassing g‐scores and *p* values. A g‐score exceeding 0.50 signifies the presence of pathogenicity [42]. R721G, A756D, Y791C, E734K, E805K, F893L, R897Q, R897G, R912Q, R912G, and M918T were the 11 nsSNPs that exhibited considerable pathogenic potential. Both of their *p* values were less than 0.05, and their g‐scores were higher than 0.60.

### 3.6. Identification of the Domains of RET

**Figure 3:**
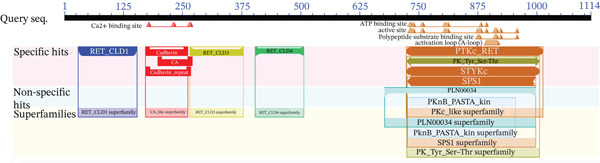
Conserved domain architecture of the RET protein predicted via Interpro server. The RET sequence comprises cadherin‐like domains at the N‐terminus, followed by RET‐specific domains (RET_CLD1, RET_CLD3 and RET_CLD4), and a conserved tyrosine kinase domain at the C‐terminus. Different colors indicate unique domain in the above figure.

The domain architecture of the RET gene reveals five functionally important regions, as shown in the conserved domain analysis. InterPro predicted five functional domains including RET_CLD1 (29‐153), CADHERIN_2 (168‐272), RET_CLD3 (265‐379), RET_CLD4 (405‐506), and PROTEIN_KINASE_DOM (724‐1016) are represented in Figure 3. The N‐terminal portion of the protein contains cadherin‐like domains (Cadherin_C and Cadherin_repeat), which are commonly involved in cell adhesion and extracellular interactions. Following this, RET_CLD1, RET_CLD3 and RET_CLD4 domains are observed, which are characteristic of RET family proteins. Notably, the C‐terminal region encompasses a well‐defined tyrosine kinase domain, which overlaps with multiple kinase families including PKc_RET, STYKc, and SPS1. This region also aligns with several superfamilies, such as PK_Tyr‐Ser‐Thr and PKc_like superfamily, indicating its conserved kinase functionality. Several key binding and active sites are also annotated, including ATP‐binding sites, catalytic sites, and substrate binding motifs, supporting its role in signal transduction.

### 3.7. PTM Site Prediction

**Figure 4:**
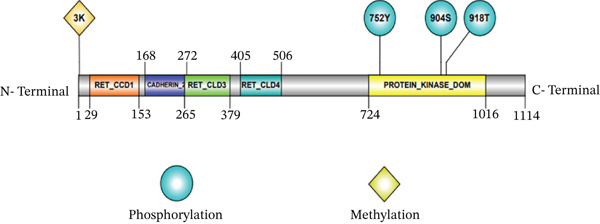
Two programs, GPS‐MSP 1.0 and NetPhos 3.1, were used with IBS software to predict PTM sites, which are phosphorylation and methylation, respectively.

By utilizing GPS‐MSP 1.0, we were able to determine that 3 K is a possible region for the methylation of RET proteins. Using NetPhos 3.1, a tool for estimating phosphorylation sites, it was also shown that the 918 T of the mutant protein, along with the 752Y and 904S of the wild‐type protein, are phosphorylation sites (Figure 4; and Table S2, see Supporting Information file).

Furthermore, ubiquitination site prediction using the UbPred tool identified 12 lysine residues (positions 108, 161, 424, 549, 716, 728, 737, 821, 869, 889, 907, and 965) as potential ubiquitination sites (Table 3). Notably, none of these predicted sites overlapped with the high‐risk nsSNP positions identified in this study, suggesting that the deleterious variants may not directly interfere with ubiquitination but could impact RET protein function through alternative structural or biochemical mechanisms.

**Table 3:** Predicted ubiquitination sites in the RET protein using UbPred.

Position	Code	Peptide	Score	Cutoff
108	K	NRSLDHSSWEKLSVRNRGFPL	0.7017	0.3566
161	K	NTSFPACSSLKPRELCFPETR	0.3903	0.3566
424	K	RRARRFAQIGKVCVENCQAFS	0.4206	0.3566
549	K	GRCEWRQGDGKGITRNFSTCS	0.4109	0.3566
716	K	VDAFKILEDPKWEFPRKNLVL	0.4001	0.3566
728	K	EFPRKNLVLGKTLGEGEFGKV	0.7498	0.3566
737	K	GKTLGEGEFGKVVKATAFHLK	0.4167	0.3566
821	K	SLRGFLRESRKVGPGYLGSGG	0.4822	0.3566
869	K	SQGMQYLAEMKLVHRDLAARN	0.4652	0.3566
889	K	NILVAEGRKMKISDFGLSRDV	0.4512	0.3566
907	K	RDVYEEDSYVKRSQGRIPVKW	0.5831	0.3566
965	K	IPPERLFNLLKTGHRMERPDN	0.4371	0.3566

### 3.8. PPI Prediction

The RET protein has been found to interact with a total of 10 different proteins, which are as follows: GFRA3, NRTN, GFRA1, ARTN, GDNF, GFRA2, GFRAL, PSPN, NCOA4, and CCDC6. These proteins were identified by the STRING service. In addition, AIP, GRB10, CBL, SHC1, GRB2 EGFR, CBLC, STAT3, GFRA1, NRTN and GDNF were identified from BioGRID database with minimum evidence 1. Among them GFRA1, NRTN and GDNF were common between both databases (Figures S1, S2, Table S3 and Table S4; see Supporting Information file).

### 3.9. Prediction of the Alterations of Protein 3D Structure Upon Mutation

**Figure 5:**
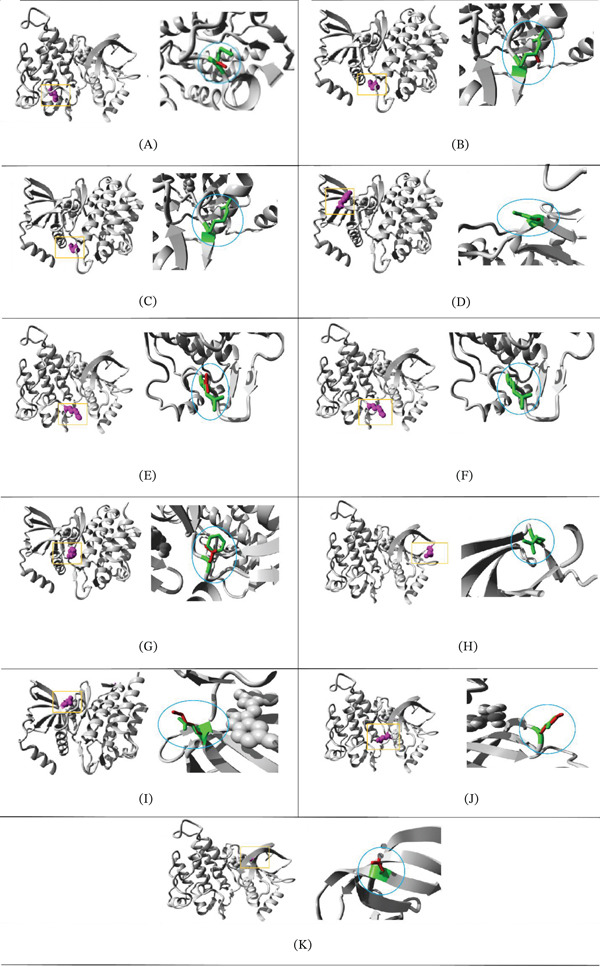
Prediction of 3D structure of mutant *RET* protein with by using Project HOPE. Our identified 11 SNPs were represented as a close view of the mutated site including (A) rs74799832 (M918T: methionine → threonine), (B) rs76087194 (R897Q: arginine → glutamine), (C) rs1060500759 (R897G: arginine → glycine), (D) rs77724903 (Y791C: tyrosine → cysteine), (E) rs78347871 (R912Q: arginine → glutamine), (F) rs1838227061 (R912G: arginine → glycine), (G) rs768188546 (F893L: phenylalanine → leucine), (H) rs1554819146 (R721G: arginine → glycine), (I) rs377767418 (E805K: glutamic acid → lysine), (J) rs1838077120 (E734K: glutamic acid → lysine), and (K) rs1838079858 (A756D: alanine → aspartic acid).

Through the examination of changes in charge, size, hydrophobicity, and conformation about the wild‐type, Project HOPE revealed that the 11 identified nsSNPs induced diverse conformational alterations arising from amino acid substitutions in the RET protein (Figure 5, Table 4 and Table S5; see Supporting Information file). Specifically, M918T (methionine → threonine), R897Q (arginine → glutamine), and R897G (arginine → glycine) disrupted polarity and charge distribution, leading to the loss of stabilizing electrostatic interactions and increased local flexibility. The Y791C (tyrosine → cysteine) and F893L (phenylalanine → leucine) mutations eliminated aromatic interactions, whereas R912Q (arginine → glutamine) and R912G (arginine → glycine) altered side‐chain chemistry, further destabilizing the region. Similarly, R721G (arginine → glycine) introduced conformational flexibility by replacing rigid or charged residues, whereas E734K (glutamic acid → lysine) and E805K (glutamic acid → lysine) caused charge reversal, potentially disturbing ionic networks. Finally, A756D (alanine → aspartic acid) introduced steric hindrance and polarity into a compact helical region.

**Table 4:** Amino acid size, charge, and hydrophobicity, among other physicochemical properties, were compared between wild‐type and mutant samples using Project HOPE server.

AA change	Wild‐type AA			Mutant‐type AA		
	Size	Charge	Hydrophobicity	Size	Charge	Hydrophobicity
M918T	Larger	–	More hydrophobic	Smaller	–	Less hydrophobic
R897Q	Larger	Positive	–	Smaller	Neutral	–
R897G	Larger	Positive	Less hydrophobic	Smaller	Neutral	More hydrophobic
Y791C	Larger	–	Less hydrophobic	Smaller	–	More hydrophobic
R912Q	Larger	Positive	–	Smaller	Neutral	–
R912G	Larger	Positive	–	Smaller	Neutral	–
F893L	Larger	–	–	Smaller	–	–
R721G	Larger	Positive	Less hydrophobic	Smaller	Neutral	More hydrophobic
E734K	Smaller	Negative	–	Larger	Positive	–
A756D	Smaller	Neutral	More hydrophobic	Larger	Negative	Less hydrophobic
E805K	Smaller	Negative	–	Larger	Positive	–

Based on structural impact prioritization and elimination of redundant mutational sites, eight high‐confidence variants were selected for further analysis such as M918T, R897Q, R897G, Y791C, F893L, E734K, A756D, and E805K. Although variants R912Q, R912G, and R721G were excluded due to positional redundancy or their comparatively lower structural impact, mainly limited to surface electrostatic alterations without significant disruption of key functional domains. In a nutshell, these amino acid substitutions through disruption of hydrogen bonding, loss of hydrophobic packing, altered electrostatics, and backbone flexibility are predicted to impair RET protein folding and functional stability, thereby contributing to its pathogenic potential.

### 3.10. GO Analysis

To functionally describe its primary targets, we looked into the biological properties of RET using a GO study. Molecular function (MF), CC, and BP were the three factors that were evaluated in the research conducted by the research group. This was accomplished by employing the geometries of squares, triangles, as well as circles to denote each group. A total of 48 GO keywords were produced, comprising 38 BP, five from CC, and five from MF (Figure S3; see Supporting Information file).

### 3.11. Validation of Potential RET Gene Variants

#### 3.11.1. Molecular Docking and Binding Interaction Analysis

Molecular docking analyses revealed that mutated RET variants generally exhibit enhanced binding affinities toward the tested ligands compared to the wild‐type RET, suggesting an increased sensitivity to reported compounds (Figures S4 and S5; see Supporting Information file). SNPs were selected based on Project HOPE and other screening processes, prioritizing mutations that induce significant changes in amino acid size, charge, and hydrophobicity. Variants such as E734K and E805K showed charge reversal, whereas R897Q/G involved loss of positive charge, potentially disrupting electrostatic interactions. Mutations like Y791C and M918T introduced notable structural and polarity changes, and A756D altered the local charge environment. These substitutions are likely to affect protein stability and ligand‐binding interactions, justifying their selection for molecular docking analysis.

E734K and R897G variants represent the significant reduction in binding interactions with two selected compounds, namely, larotrectinib and sorafenib that suggest may confer resistance. On the other hand, the wild‐type RET and its seven mutated proteins exhibited a high binding affinity for entrectinib drug (E734K: −9.7 kcal/mol, A756D: −10.4 kcal/mol, Y791C: −9.9 kcal/mol, F893L: −10.7 kcal/mol, R897Q: −10.0 kcal/mol, R897G: −9.9 kcal/mol, and M918T: −10.4 kcal/mol, compared to wild‐type (RET: −9.1 kcal/mol) as shown in Table 5.

**Table 5:** Docking results (kcal/mol) of RET mutations with four ligands.

Ligands	RET	E734K	A756D	Y791C	F893L	R897Q	R897G	M918T	E805K
Dabrafenib	−8.5	−9.1	−8.5	−9.6	−9.1	−8.4	−9.1	−10.0	−8.5
Entrectinib	−9.1	−9.7	−10.4	−9.9	−10.7	−10.0	−9.9	−10.4	−7.8
Larotrectinib	−10.3	−8.7	−8.7	−9.6	−9.8	−9.2	−8.7	−9.8	−8.1
Sorafenib	−9.0	−7.4	−8.6	−9.6	−9.4	−8.7	−7.8	−9.3	−8.4

The molecular docking analysis also revealed that entrectinib established stable interactions with both wild‐type and mutant RET proteins, primarily through hydrogen bonding, van der Waals forces, *π*–*π* interactions, and hydrophobic contacts (Figure S4; see Supporting Information file). In the E734K (Figure S4A; see Supporting Information file) and A756D (Figure S4B; see Supporting Information file) mutants, strong hydrogen bonds with GLU739 and ASP737, along with *π*–cation interactions, contributed to ligand stabilization. The Y791C (Figure S4C; see Supporting Information file) and F893L (Figure S4D; see Supporting Information file) mutants showed altered interaction profiles, with multiple hydrogen bonds (ASN879, ASP892, and SER891) and *π*–*π* stacking compensating for residue substitutions. Similarly, the M918T (Figure S4E; see Supporting Information file), R897Q (Figure S4F; see Supporting Information file), R897G (Figure S4G; see Supporting Information file), and E805K (Figure S4H; see Supporting Information file) mutants that maintained binding through robust hydrogen bonding with GLU739, ASN879, and VAL899 was supported by van der Waals and alkyl contacts. Finally, in the wild‐type RET protein (Figure S4I; see Supporting Information file), entrectinib interacted consistently with GLU739, ASN879, and ASP892, whereas hydrophobic residues such as LEU833 and VAL738 further stabilized the complex. Collectively, these results indicate that entrectinib retains strong binding affinity across wild‐type and mutant RET proteins, highlighting its potential as a robust inhibitor against RET‐associated alterations.

#### 3.11.2. Survival Analysis

**Figure 6:**
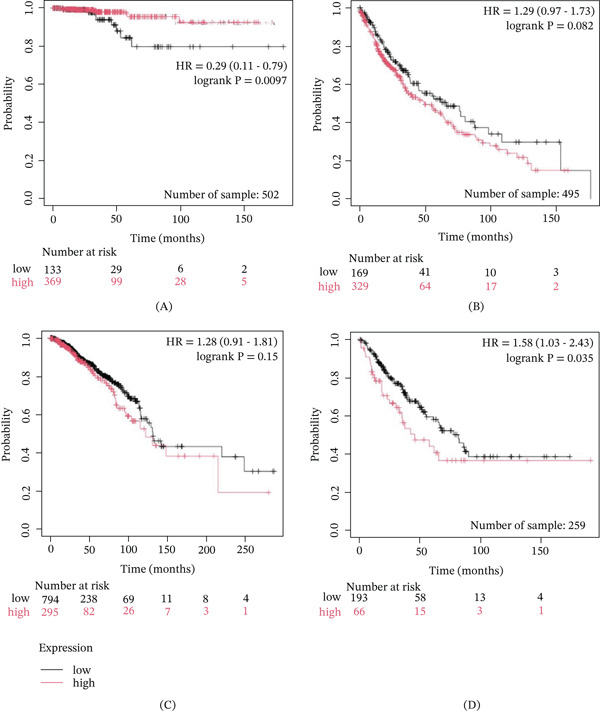
Microarray data generated from the Kaplan‐Meirer plotter were utilized to ascertain the levels of RET gene expression as well as the survival rates of individuals who were diagnosed with a variety of cancers. As shown in survival plot, (A) represents a plot of thyroid cancer, sample = 502; (B) represents a plot of squamous cell lung carcinoma, sample = 495; (C) represents a plot of breast cancer, sample = 1089; and (D) represents a plot of sarcoma, sample = 259.

The Kaplan‐Meier survival curves demonstrate the relationship between gene expression levels and overall patient survival. In Figure 6A, high gene expression is significantly associated with improved survival (HR = 0.29, 95% CI: 0.11–0.79, log‐rank *p* = 0.0097), indicating a strong protective effect and suggesting that the gene may serve as a favorable prognostic marker on thyroid cancer. Conversely, Figure 6B shows that high expression is linked to significantly poorer survival (HR = 1.58, 95% CI: 1.03–2.43, *p* = 0.035), pointing to a potential oncogenic role on squamous cell lung carcinoma. Figure 6C,D display a trend toward reduced survival with high expression (HR = 1.29 and 1.28, respectively), but these differences are not statistically significant (*p* > 0.05) on breast cancer and sarcoma Collectively, these findings suggest that the prognostic impact of this gene may vary across different clinical or molecular perspectives, highlighting its potential relevance in patient risk and guiding treatment decisions.

#### 3.11.3. Molecular Dynamics Simulation Studies

Based on binding affinity analysis, entrectinib and selected seven mutants (E734K, A756D, Y791C, F893L, R897Q, R897G and M918T) were selected for detailed molecular dynamics simulations to evaluate binding stability, structural compactness, and conformational fluctuations compared to wild‐type RET protein.

##### 3.11.3.1. Protein Ligand Stability Analysis

**Figure 7:**
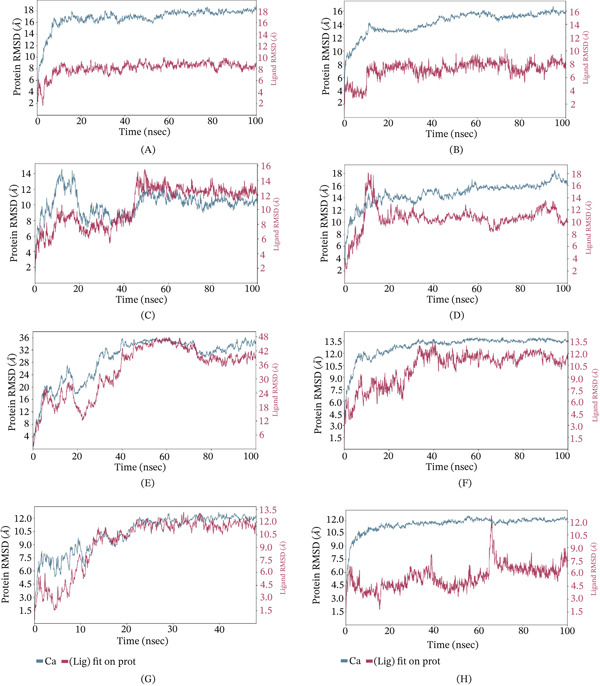
Root mean square deviation (RMSD) plots of RET receptor variants during 100 ns molecular dynamics simulations. (A) A756D, (B) E734K, (C) F893L, (D) M918T, (E) R897G, (F) R897Q, (G) Y791C, and (H) RET wild‐type. Protein backbone RMSD (blue, *y*‐axis) and ligand RMSD (red, *y*‐axis) are plotted against simulation time (*x*‐axis).

Molecular dynamics simulation analysis of the RET receptor variants revealed distinct differences in structural stability among the mutant and wild‐type complexes. The protein backbone RMSD and ligand RMSD trajectories (100 ns) demonstrated that A756D (Figure 7A), E734K (Figure 7B), and R897Q (Figure 7F) displayed moderate stability with gradual convergence after the initial equilibration phase, whereas F893L (Figure 7C) and M918T (Figure 7D) showed higher fluctuations, indicating reduced conformational stability. R897G (Figure 7E) and Y791C (Figure 7G) exhibited the greatest deviations throughout the simulation, suggesting that these mutations may significantly destabilize the protein–ligand complex. In comparison, the RET wild‐type complex (Figure 7H) remained relatively stable with minimal fluctuations. However, the comparative analysis indicates that specific RET mutations alter the dynamic behavior of the protein, potentially influencing ligand binding stability and functional outcomes.

##### 3.11.3.2. Protein Ligand Fluctuations Analysis

**Figure 8:**
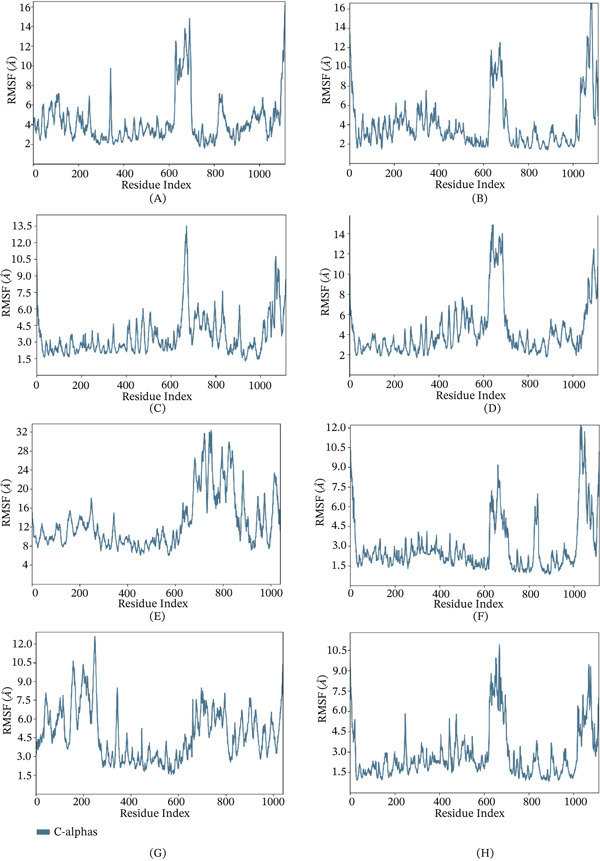
Root mean square fluctuation (RMSF) profiles of RET receptor variants obtained from 100 ns molecular dynamics simulations. Panels represent (A) A756D, (B) E734K, (C) F893L, (D) M918T, (E) R897G, (F) R897Q, (G) Y791C, and (H) RET wild‐type. RMSF values (*y*‐axis) are plotted against the residue index (*x*‐axis), highlighting residue‐level flexibility of C*α* atoms in wild‐type and mutant complexes.

The RMSF analysis was performed to investigate the residue‐wise flexibility of the RET receptor and its variants over the 100 ns simulation trajectory. Mutants A756D (Figure 8A), E734K (Figure 8B), and R897Q (Figure 8F) exhibited moderate fluctuations, mainly localized in loop regions, suggesting restricted but notable flexibility, with peaks at residues 650–675, 600–650, and 620–670, respectively, with peaks at residues 650–675, 600–650, and 620–670, respectively. F893L (Figure 8C) and M918T (Figure 8D) showed higher fluctuations across several domains (2–9 Å), indicating increased conformational mobility compared to other variants. The R897G mutant (Figure 8E) displayed the highest level of fluctuations, particularly in the C‐terminal regions (13–15 Å), reflecting considerable structural instability. Y791C (Figure 8G) also demonstrated elevated fluctuations in selected regions (10–12 Å), though less pronounced than R897G. In contrast, the wild‐type RET (Figure 8H) exhibited relatively lower fluctuations across most residues (2–6 Å), with peaks observed in loop regions 650–780 and the terminal region 1050–1070 residues (16 Å), indicating stable structural dynamics. In a nutshell, these findings suggest that specific RET mutations contribute to altered dynamic behavior and enhanced residue‐level flexibility, which may influence protein stability and ligand recognition.

##### 3.11.3.3. Rg Analysis

The Rg analysis was conducted to evaluate the compactness and overall stability of RET mutants A756D, E734K, F893L, M918T, R897G, R897Q and Y791C during a 100 ns molecular dynamics simulation. Most variants maintained mean Rg values in the range of 5.0–5.4 Å, indicating retention of a compact conformation throughout the trajectory (Figure S6; see Supporting Information file).

##### 3.11.3.4. Protein Ligand Interaction Analysis

Molecular interaction analysis of the docked complexes revealed distinct binding patterns of the studied ligand with RET mutants A756D, E734K, F893L, M918T, R897G, R897Q and Y791C compared with the RET wild‐type. In contrast, the RET wild‐type displayed the richest network of hydrogen bonds and hydrophobic contacts involving multiple residues within the ATP‐binding cleft, indicating a more extensive and potentially stronger ligand engagement than most mutant complexes (Figure S7; see Supporting Informaion file).

#### 3.11.4. Clinical and Cancer Databases Validation

To validate the predicted deleterious nsSNPs, the selected variants were cross‐referenced with curated databases, including ClinVar, LOVD and CKB (Table 6). Among them, p.Met918Thr (rs74799832), located in Exon 16, is well‐established as pathogenic/likely pathogenic and is functionally characterized as a gain‐of‐function mutation, with strong clinical associations with multiple endocrine neoplasia (MEN2A and MEN2B), pheochromocytoma, familial medullary thyroid carcinoma (FMTC), and various malignant carcinomas. In contrast, p.Arg897Gln (rs76087194) is classified as a risk factor and is associated with loss‐of‐function effects, contributing to susceptibility to Hirschsprung disease. The variant p.Phe893Leu (rs768188546) is also predicted to be deleterious and is annotated as a loss‐of‐function mutation, although it lacks detailed clinical classification data.

**Table 6:** Functional and clinical annotation of prioritized *RET* missense variants. Variants were curated from dbSNP and cross‐referenced with ClinVar, Leiden Open Variation Database (LOVD) and Cancer Knowledge Base (CKB) databases.

Location	Codon Change	Variant	DbSNP ID	Prediction	Classification (level)	Database	CKB Annotation (level)	Phenotype
Exon 16	ATG‐ACG	p.Met918Thr	Rs74799832	Deleterious	Pathogenic, likely pathogenic	LOVD, ClinVar	Gain of function	MEN2A, MEN2B, Pheochromocytom, and FMTC
Exon 15	CGA>CAA	p.Arg897Gln	rs76087194	Deleterious	Risk factor	ClinVar	Loss of function	HSCR, susceptibility to 1
Exon 15	CGA>GGA	p.Arg897Gly	rs1060500759	Deleterious	VUS	ClinVar	Unknown	MEN2
Exon 14	TAT>TGT	p.Tyr791Cys	rs77724903	Deleterious	VUS	ClinVar	Unknown	MEN2, HSCR
Exon 15	TTC>TTA	p.Phe893Leu	rs768188546	Deleterious	N/A	ClinVar	Loss of function	N/A
Exon 13	GAA>AAA	p.Glu734Lys	rs1838077120	Deleterious	VUS	ClinVar	Loss of function	MEN2
Exon 13	GCC>GAC	p.Ala756Asp	rs1838079858	Deleterious	VUS	ClinVar	Unknown	HSCR, MEN2
Exon 14	GAG>AAG	p.Glu805Lys	rs377767418	Deleterious	VUS	ClinVar	Unknown	MEN2

Abbeviations: Hirschsprung disease (HSCR); Multiple endocrine neoplasia Type 2 (MEN2); Multiple Endocrine Neoplasia Type 2A (MEN2A); Multiple Endocrine Neoplasia Type 2B (MEN2B); Familial medullary thyroid carcinoma (FMTC).

The remaining variants, including p.Arg897Gly (rs1060500759), p.Tyr791Cys (rs77724903), p.Glu734Lys (rs1838077120), p.Ala756Asp (rs1838079858), and p.Glu805Lys (rs377767418), are predominantly categorized as variants of uncertain significance (VUS) in ClinVar, with limited or unknown functional annotations. However, these variants have been reported in association with clinically relevant phenotypes such as MEN2 and Hirschsprung disease. Notably, the majority of the selected SNPs are located within Exons 14–16, corresponding to the RET kinase domain, a functionally critical region involved in catalytic activity and ligand binding. The presence of these variants in clinically curated databases, combined with their predicted deleterious effects, underscores their potential structural and functional impact.

## 4. Discussion

Multiple coding and noncoding variants have been recorded within the RET gene. Loss‐of‐function RET mutations are linked to Hirschsprung disease, whereas gain‐of‐function RET mutations are associated with cancers such as FMTC and MEN Types 2A and 2B [68, 69]. Understanding the functional impact of genetic variants is critical for accurate diagnosis and the development of targeted therapies. Although traditional experimental methods for analyzing SNPs yield valuable data, they are often resource‐intensive and time‐consuming. Computational approaches present a cost‐effective and efficient alternative for predicting the effects of SNPs on protein structure and function. Nevertheless, the reliability and biological relevance of these in silico predictions require thorough validation to ensure accurate identification of clinically significant variants [70, 71]. A comprehensive strategy employing 10 distinct prediction servers, including SIFT, PROVEAN, Predict‐SNP, SNAP2, PolyPhen‐1, SNP&GO, PhD‐SNP, PANTHER, PolyPhen‐2, and FATHMM, was utilized to find the most harmful nsSNPs. All 10 analyses revealed that the nsSNPs M918T, R897Q, R897G, C620Y, C620W, Y791C, R912Q, R912G, R817S, G885R, S922Y, F893L, R813W, R721G, P720H, P996R, E734K, and A756D consistently exhibited detrimental effects, suggesting potential disease consequences. In addition, research conducted with the MUpro and I‐Mutant 2.0 algorithms demonstrated that certain nsSNPs were associated with a reduction in the stability of proteins. All mutations, except S922Y, were recognized as enhancing stability based on RI and DDG, as alterations in protein stability influence its structural conformation and functions [72, 73] . Subsequent study utilizing the mutation 3D server identified a correlation between specific nsSNPs (P720H, R721G, A756D, Y791C, E734K, F893L, R897Q, R897G, R912Q, R912G, and M918T) and cancer.

Alterations in protein stability influence structural conformation, hence regulating protein function [74, 75]. In this study, possible chemical changes brought on by mutations which might impact the RET protein′s nature or function were investigated using the MutPred2 tool. All detected harmful SNPs were categorized as “pathogenic” based on their g and *p* scores. Five RET functional domains were found by InterPro, including one cysteine‐rich protein‐kinase domain and 4 cadherin‐like domains. The RET‐CLD1 domain is essential for ligand–coreceptor binding and supports CLD2 folding within their distinct clamshell‐like structure. CLD1 also contains two binding sites for GDNF receptor *α*1. Calcium‐binding motifs, characteristic of classical cadherins, are found between CLD2 and CLD3, whereas CLD4 is crucial for proper CRD folding [76, 77]. The results of this study on the reliability of GPS‐MSP 1.0 as well as NetPhos 3.1 are interesting. In disagreement with the predictions made by NetPhos 3.1that the wild‐type protein would have phosphorylation sites at 752Y and 904S, and the mutant form at 918TGPS‐MSP 1.0 discovered a methylation site at 3 K. Previous research demonstrated that phosphorylation sites are often exploited in drug design to target dysregulated pathways, especially in cancer and neurodegenerative disorders. Accurate identification of these sites is crucial for the development of selective and effective therapeutics [78].

PPI analysis indicates that the RET protein is integral to multiple essential activities. Glial cell‐derived neurotrophic factor (GDNF) is the source of the RET protein and other extracellular signaling molecules [79]. RET is bound to the dimeric development factor protein GDNF. According to the transforming growth factor‐beta (TGF‐*β*) superfamily, it is a member of the superfamily [80], along with neurturin (NRTN) [81], persephin (PSPN) [82], and artemin (ARTN) [83]. Interactions between ligands and the GDNF receptor alpha proteins (GFR*α*1, GFR*α*2, GFR*α*3, and GFR*α*4) activate the RET protein. Specifically, these receptors are coreceptors that are anchored by glycosylphosphatidylinositol (GPI) [84]. The exterior domains of RET, the co‐receptor, and the ligand participate in the assembly of ternary complexes with the following partners:(i) GFR*α*1 and RET in GDNF; (ii) RET and GFR*α*2 in NRTN; and (iii) RET and GFR*α*3 in ARTN. These ternary compounds facilitate the dimerization of the RET protein [85]. Notably, BRCA1, RB1, and E2F1 are well‐documented regulators of cell cycle control and tumor suppression [86–88]. Additionally, HDAC1 and WDR5 are implicated in epigenetic regulation and transcriptional modulation [89, 90]. The identification of VCP further highlights involvement in protein quality control and ER‐associated degradation pathways [91].

The characteristics of variant residues, including their physical, chemical, and molecular properties, may occasionally differ from those of the original structure. According to the results of our research, wild‐type and mutant kinds are very different from one another in terms of charge, size, and hydrophobic resistance. The balance of positive (Arg, Lys) and negative (Asp, Glu) charges in the RET protein is essential for maintaining structural stability and enabling key functions like dimerization, ligand binding, and kinase activity. Mutations that disrupt this charge distribution can impair signaling, contributing to disorders such as Hirschsprung disease and multiple endocrine neoplasia [70, 92]. When compared to the wild‐type, the amino acid sizes that are produced by the mutations P720H, E734K, and A756D are significantly larger. Research concluded that mutations in protein which increased amino acid size can introduce steric hindrance, disrupting local folding and structural integrity [93]. Additionally, the R897Q, R897G, R912Q, R912G, R721G, E734K, and A756D mutations demonstrate a modified amino acid composition compared to the mutant version may also impair native interactions, on the other hand, the R897G, Y791C, and R721G mutations exhibit enhanced hydrophobicity in the mutant form, which may influence hydrophobic interactions or membrane association. Consequently, onring aggregation and folding, these unforeseen alterations in proteins may lead to a loss of thermodynamic stability, alter its signaling, and finally contributing to disease pathogenesis [94, 95]. Variants near the glycine‐rich loop (E734K), hinge region (E805K), or hydrophobic contact surfaces (A756D, F893L) can alter inhibitor binding geometry by disrupting hydrophobic packing or key hydrogen‐bond interactions [96]. Mutations in the activation loop or adjacent segments (such as Y791C and R897Q/G) may potentially influence local kinase dynamics or flexibility, which could indirectly affect inhibitor engagement; however, experimental validation is needed.

Molecular docking of eight RET inhibitors against eight RET variants revealed mutation‐specific alterations in binding affinities. Among the tested ligands, entrectinib showed consistently strong binding across all RET variants (−9.7 to −10.7 kcal/mol), indicating broad‐spectrum inhibitory potential. In contrast, larotrectinib bound most strongly to wild‐type RET (−10.3 kcal/mol) but less effectively to E734K, A756D, and R897G (−8.7 kcal/mol). Dabrafenib and sorafenib also displayed variable affinities, with sorafenib weakest against E734K (−7.4 kcal/mol) and R897G (−7.8 kcal/mol), reflecting potential mutation‐driven resistance. Mutations in the RET kinase domain influence drug response through direct disruptions of inhibitor binding sites or long‐range conformational effects. Preclinical mutagenesis screens have identified several RET substitutions within the ATP‐binding pocket and adjacent structural elements that confer resistance to multikinase inhibitors [97]. Mutations in the glycine‐rich loop (such as E732K) and gatekeeper or hinge region residues (e.g., V804L/M, Y806N, and G810S) cause resistance by altering the shape and dynamics of the drug binding cavity, reducing access or contacts for inhibitors like cabozantinib, lenvatinib, and vandetanib. These changes often disrupt key hydrophobic interactions or hydrogen bonds essential for stable inhibitor engagement [98]. Previous research also demonstrated that the M918T substitution, although outside the binding site, has been repeatedly shown to increase the IC_50_ values of several MKIs (e.g., cabozantinib and vandetanib) relative to wild‐type RET. This suggests that distal mutations can alter the dynamic conformational equilibrium of the kinase domain, subtly shifting the overall structure into conformations less favorable for inhibitor binding and confer to resistance [98–100]. Our docking analysis shows that RET SNPs E734K, A756D, F893L, and E805K have lower binding affinities than the wild‐type, likely due to altered local interactions or pocket dynamics, which may reduce therapeutic efficacy. In contrast, entrectinib maintains strong binding across all variants, highlighting the importance of SNP‐specific considerations in RET‐targeted drug design and supporting further experimental validation [101].

Oncogenic RET ligand‐independent phosphorylation can be triggered by mutations, which in turn activate downstream signaling pathways. This sustained activation causes morphological alterations and tumor development [102]. Kaplan‐Meier plotter analyses demonstrate that dysregulation of the RET gene has predictive relevance and affects overall survival rates among individuals with thyroid cancer, squamous cell lung carcinoma, breast cancer, and sarcoma [103]. Increased RET expression levels correlated with lower survival rates within squamous cell lung carcinoma, breast cancer, and sarcoma. These data suggest that any dysregulation of RET expression can profoundly affect patient survival in these malignancies.

Molecular dynamics simulations of RET–entrectinib complexes reveal that specific RET mutations markedly affect ligand binding stability, compactness, and flexibility [104]. Mutants A756D, E734K, and R897Q stabilize after 20 ns with backbone RMSD 2.5 Å and ligand RMSD 1.8 Å. On the other hand, R897G and Y791C exhibit the largest deviations, indicating pronounced destabilization. In molecular dynamics simulations, backbone RMSD values of 1–3 Å indicate stable protein structures, whereas ligand RMSD below 2–5 Å reflects stable binding; higher values suggest weak interactions [105]. RMSF analysis indicated that A756D, E734K, and R897Q exhibited lower fluctuations (3–9 Å), reflecting localized flexibility, whereas R897G and Y791C showed markedly higher fluctuations (10–15 Å), suggesting significant structural instability. The wild‐type remained comparatively stable (2–6 Å) with minor peaks in loop and terminal regions. Research demonstrated that RMSF evaluates the flexibility of individual residues, where lower RMSF values (typically 1–3 Å) indicate stable, rigid regions, and higher values (> 5 Å) reflect flexible loops or binding‐site mobility [106]. The Rg analysis demonstrated that most RET mutants retained compact conformations (5.0–5.4 Å), comparable to the wild‐type. Mutants such as A756D, F893L, and R897G remained highly stable, whereas M918T and Y791C exhibited convergence toward stable conformations after initial deviations. By contrast, E734K and R897Q showed larger fluctuations approaching 6.2 Å, suggesting reduced stability. Scientists demonstrated that lower Rg values indicates a more compact and stable protein structure, whereas higher Rg values suggest increased flexibility and reduced structural stability [107]. In our study, stabilizing mutations such as A756D and Y791C supported multiple hydrogen bonds and hydrophobic interactions, whereas E734K, M918T, and R897G exhibited limited polar contacts. Besides, F893L displayed an extended hydrophobic engagement, and R897Q showed minimal polar interactions, indicating weaker ligand accommodation. In comparison, the wild‐type formed the most extensive hydrogen‐bonding and hydrophobic network within the ATP‐binding cleft, reinforcing its superior binding stability. Molecular dynamics simulations revealed that RET mutations differentially affect protein stability, with A756D, E734K, and R897Q showing lower RMSD, RMSF, and Rg values indicative of stable, compact structures, whereas R897G and Y791C exhibit higher values reflecting increased flexibility and structural destabilization. In addition, protein–ligand interaction analysis, including hydrogen bonds, hydrophobic contacts, and salt bridges, quantifies the stability and specificity of ligand binding, with higher interaction fractions or persistent contacts indicating stronger binding and potential efficacy [108, 109].

Mutations in the RET gene are key drivers of MEN2 and MTC. Well‐established variants such as C634 and M918T are linked to aggressive disease and early metastasis in MEN2A and MEN2B, whereas A883F is associated with a comparatively less aggressive MTC phenotype [110, 111]. Beyond these, polymorphisms at codon 836 and several synonymous variants (p.Cys609Cys, p.Ile788Ile, p.Ser891Ser, and p.Tyr806Tyr) have been reported in MTC, suggesting potential roles in disease susceptibility [112]. Moreover, variants including V202M, E480K, P973L, D771N, and splice‐site mutations (IVS10‐2A/G and IVS19‐9C/T) are associated with Hirschsprung′s disease, highlighting the diverse functional impact of RET alterations [113]. In line with these observations, our analysis identified several *RET* SNPs within the kinase domain (Exons 15–16) that are predicted to be deleterious but remain largely classified as VUS. This supports previous findings that not all clinically relevant RET variants are fully characterized, particularly those outside well‐known hotspots. Notably, although earlier studies primarily emphasize highly recurrent mutations such as M918T, our findings highlight additional less‐characterized variants (e.g., E734K, A756D, F893L, and E805K) that may also have functional importance. This expands the spectrum of potentially relevant *RET* alterations beyond traditionally studied mutations.

This study demonstrates the utility of in silico analyses in identifying potentially deleterious *RET* nsSNPs, particularly within the kinase domain (Exons 15–16), that may influence protein stability and drug interactions. Notably, variants such as E734K, A756D, F893L, and E805K showed reduced binding affinities compared to the wild‐type, suggesting possible impacts on inhibitor engagement, whereas entrectinib consistently maintained strong interactions across all variants, highlighting its potential as a robust *RET*‐targeted therapeutic. However, these findings are limited by reliance on computational predictions and available database annotations, with most identified variants lacking sufficient clinical or experimental validation. Additionally, this study focused only on coding SNPs and did not consider noncoding regions, inheritance patterns, or regulatory mechanisms that may also contribute to disease susceptibility. Therefore, further validation through in vitro and in vivo studies, including site‐specific mutagenesis, PCR‐based genotyping, and functional assays, is essential to confirm the clinical significance of these variants and to assess their potential as a therapeutic target. In a nutshell, integrating computational approaches with experimental and clinical data will be crucial for improving the understanding of RET‐associated diseases and advancing precision oncology strategies.

## 5. Conclusion

This study presents the first comprehensive in silico characterization of deleterious *RET* nsSNPs, particularly within the kinase domain (Exons 15‐16), elucidating their potential effects on protein stability, structural integrity, and drug response. Eleven deleterious variants including M918T, R897Q, R897G, Y791C, R912Q, R912G, F893L, R721G, P720H, E734K, and A756D, which were identified in highly conserved regions, suggesting functional relevance. Notably, M918T is associated with Type 2B multiple endocrine neoplasia. Variants such as E734K, A756D, F893L, and E805K were predicted to alter key physicochemical properties and reduce inhibitor binding, potentially modulating therapeutic sensitivity. In contrast, entrectinib consistently exhibited strong binding and enhanced stability across both wild‐type and mutant *RET* proteins, highlighting its potential as a mutation‐tolerant inhibitor. Although findings provide a foundation for genetic screening and personalized therapy, they are limited by computational predictions, necessitating further in vitro and in vivo validation to confirm clinical relevance and inform *RET*‐targeted precision oncology strategies.

## Author Contributions

N.A. and M.A.H.: Conceptualization, Formal analysis, Methodology, Writing – original draft, Validation, Visualization, and Investigation; M.T.H.: Data curation, Software, and Resources; M.M.U.: Software, Writing – original draft, and Writing – review and editing; M.K.: Writing – original draft and Writing – review and editing; M.S.I.: Writing – review and editing; S.A.A.: Writing – review and editing; M.H.R.: Conceptualization, Project administration, Supervision, Writing – original draft, and Writing – review and editing; N.A. and M.A.H. contributed equally to this work.

## Funding

No funding was received for this manuscript.

## Ethics Statement

The authors have nothing to report.

## Consent

The authors have nothing to report.

## Conflicts of Interest

The authors declare no conflicts of interest.

## Supporting information


**Supporting Information** Additional supporting information can be found online in the Supporting Information section. Table S1: Functional and structural modifications of *RET* predicted by MutPred2. Table S2: Prediction of phosphorylation sites in wild‐type and mutant‐type RET using NetPhos 3.1. Table S3: Functions of proteins connected with *RET* from STRING database. Table S4: RET interaction network (IntAct‐derived Proteins). Table S5: Prediction of 3D model structure of *RET* protein with ribbon‐presentation by using Project HOPE server. Figure S1: Protein–protein interaction network of RET protein examined by STRING database. RET is the most connected node (highlighted in red), indicating it is a hub protein in this network. The different colored lines (edges) illustrate various types of connection of the interactions. Figure S2: Protein–protein interaction network analysis from BioGrid database. Circular shaped indicates interacted proteins and solid line indicates interaction between respective proteins. Figure S3: Gene Ontology (GO) analysis utilizing biological process (BP), cellular component (CC), and molecular function (MF) based on gene number and −log_10_ (*p* value) via SRplot. Figure S4: Top molecular binding 2D interaction analysis of entrectinib drug against seven mutant structures including (A) E734K, (B) A756D, (C) Y791C, (D) F893L, (E) R897Q, (F) M918T, (G) R897G, (H) E805K, and (I) wild‐type RET protein. Figure S5: Binding interaction analysis of four drug compounds (dabrafenib, entrectinib, larotrectinib, and sorafenib) against eight mutant structures (A756D, E734K, F893L, M918T, R897G, R897Q, Y791C, and E805K) and wild‐type RET protein. Left side indicates the 2D interaction and right side indicates the 3D interaction. Figure S6: Radius of gyration (Rg) plots of RET mutants and wild‐type over a 100 ns molecular dynamics simulation. (A) A756D, (B) E734K, (C) F893L, (D) M918T, (E) R897G, (F) R897Q, (G) Y791C, and (H) RET wild‐type. The *x*‐axis represents simulation time (ns), and the *y*‐axis represents the Rg (Å). Figure S7:Protein ligand interaction analysis via 2D diagram of RET mutants and wild‐type over a 100 ns molecular dynamics simulation. (A) A756D, (B) E734K, (C) F893L, (D) M918T, (E) R897G, (F) R897Q, (G) Y791C, and (H) RET wild‐type.

## Data Availability

The datasets generated and analyzed in this study are available upon request from the corresponding author (Md. Habibur Rahman). The nsSNP data for the RET gene were retrieved from the dbSNP database (https://www.ncbi.nlm.nih.gov/snp), and UniProt (https://www.uniprot.org ) provided the sequence acquisition. Functional predictions of nsSNPs were performed using bioinformatics tools, including SIFT (https://sift.bii.a‐star.edu.sg), PolyPhen‐2 (http://genetics.bwh.harvard.edu/pph2), and PROVEAN (http://provean.jcvi.org). Additional stability and conservation analyses utilized I‐Mutant 2.0 (https://folding.biofold.org/i‐mutant/i‐mutant2.0.html) and Consurf (https://consurf.tau.ac.il). Protein–protein interaction network and pathway analysis was done by STRING (https://string‐db.org/) and Enrichr (https://maayanlab.cloud/Enrichr/) databases, respectively. Finally, survival analysis was performed using the KM Plotter database (https://kmplot.com/analysis/), which provides survival data for cancer patients across various malignancies. These publicly available resources and tools ensure the reproducibility of the study findings.
