# Supplementary material for: Modeling the Effects of Single Nucleotide Polymorphisms (SNPs) on the Structure and Function of the Human RET Gene: An In Silico Study
Source: Hum Mutat. 2026 Jun 27;2026:8848146. doi: 10.1155/humu/8848146 (PMC13309746; doi:10.1155/humu/8848146)
Supplement: Supplementary file 1 — Supporting Information Additional supporting information can be found online in the Supporting Information section. Table S1: Functional and structural modifications of RET predicted by MutPred2. Table S2: Prediction of phosphorylation sites in wild‐type and mutant‐type RET using NetPhos 3.1. Table S3: Functions of proteins connected with RET from STRING database. Table S4: RET interaction network (IntAct‐derived Proteins). Table S5: Prediction of 3D model structure of RET protein with ribbon‐presentation by using Project HOPE server. Figure S1: Protein–protein interaction network of RET protein examined by STRING database. RET is the most connected node (highlighted in red), indicating it is a hub protein in this network. The different colored lines (edges) illustrate various types of connection of the interactions. Figure S2: Protein–protein interaction network analysis from BioGrid database. Circular shaped indicates interacted proteins and solid line indicates interaction between respective proteins. Figure S3: Gene Ontology (GO) analysis utilizing biological process (BP), cellular component (CC), and molecular function (MF) based on gene number and −log10 (p value) via SRplot. Figure S4: Top molecular binding 2D interaction analysis of entrectinib drug against seven mutant structures including (A) E734K, (B) A756D, (C) Y791C, (D) F893L, (E) R897Q, (F) M918T, (G) R897G, (H) E805K, and (I) wild‐type RET protein. Figure S5: Binding interaction analysis of four drug compounds (dabrafenib, entrectinib, larotrectinib, and sorafenib) against eight mutant structures (A756D, E734K, F893L, M918T, R897G, R897Q, Y791C, and E805K) and wild‐type RET protein. Left side indicates the 2D interaction and right side indicates the 3D interaction. Figure S6: Radius of gyration (Rg) plots of RET mutants and wild‐type over a 100 ns molecular dynamics simulation. (A) A756D, (B) E734K, (C) F893L, (D) M918T, (E) R897G, (F) R897Q, (G) Y791C, and (H) RET wild‐type. The x‐axis represents s [file HUMU-2026-8848146-s001.docx]

**Modelling the Effects of Single Nucleotide Polymorphisms (nsSNPs) on the Structure and Function of the Human *RET* Gene: An *In-Silico* Study**

Nabilah Anzoom^1,2^, Md. Arju Hossain^3^, Md. Tanvir Hossain^4^, Md. Moin Uddin^4^, Mahfuj Khan^1^, Md Shofiqul Islam^5^, Siddique Akber Ansari^6^ and Md Habibur Rahman^1,7,*^

**Table S1 |** Functional and structural modifications of *RET* predicted by MutPred2.

| Mutation | Actionable/confident hypothesis | g-value | p-value | Probability | Affected PROSITE and ELM Motifs |
| --- | --- | --- | --- | --- | --- |
| M918T | Altered ordered interface | 0.925 | 0.02 | 0.30 | ELME000053, ELME000064, ELME000220, ELME000301, PS00006 |
|  | Altered DNA binding |  | 0.02 | 0.19 |  |
| R897Q | Loss of Allosteric site at R897Q | 0.901 | 2.9e-04 | 0.44 | ELME000202, PS00007 |
|  | Altered metal binding |  | 7.4e-03 | 0.42 |  |
|  | Gain of Relative solvent accessibility |  | 8.0e-03 | 0.30 |  |
|  | Gain of Catalytic site at D892 |  | 3.5e-03 | 0.26 |  |
|  | Altered DNA binding |  | 0.03 | 0.18 |  |
|  | Altered Transmembrane protein |  | 0.01 | 0.16 |  |
|  | Loss of Sulfation at Y900 |  | 9.7e-03 | 0.04 |  |
| R897G | Loss of Allosteric site at R897Q | 0.950 | 4.9e-06 | 0.64 | ELME000085, PS00007 |
|  | Altered metal binding |  | 8.3e-04 | 0.34 |  |
|  | Gain of Relative solvent accessibility |  | 2.5e-03 | 0.33 |  |
|  | Loss of Catalytic site at D892 |  | 6.7e-03 | 0.24 |  |
|  | Altered DNA binding |  | 0.03 | 0.19 |  |
|  | Altered Transmembrane protein |  | 0.01 | 0.16 |  |
|  | Loss of Sulfation at Y900 |  | 9.5e-03 | 0.05 |  |
|  | Loss of Disulfide linkage at C618 |  | 0.05 | 0.11 |  |
| Y791C | Altered metal binding | 0.835 | 4.9e-03 | 0.50 | None |
|  | Loss of Strand |  | 6.5e-03 | 0.28 |  |
|  | Altered Transmembrane protein |  | 1.1e-03 | 0.25 |  |
|  | Loss of Allosteric site at Y791 |  | 0.02 | 0.24 |  |
|  | Gain of Catalytic site at C794 |  | 9.1e-03 | 0.22 |  |
| R912Q | Gain of Loop | 0.766 | 1.8e-03 | 0.32 | ELME000202, ELME000233 |
|  | Altered DNA binding |  | 2.4e-03 | 0.31 |  |
|  | Altered ordered interface |  | 0.02 | 0.26 |  |
|  | Gain of Strand |  | 0.05 | 0.26 |  |
|  | Gain of Relative solvent accessibility |  | 0.04 | 0.24 |  |
|  | Loss of Allosteric site at R912 |  | 0.05 | 0.19 |  |
|  | Altered Transmembrane protein |  | 0.04 | 0.11 |  |
|  | Loss of Methylation at K907 |  | 0.04 | 0.11 |  |
|  | Gain of Catalytic site at R912 |  | 0.04 | 0.09 |  |
| R912G | Altered ordered interface | 0.884 | 1.5e-03 | 0.39 | ELME000202, ELME000233 |
|  | Altered DNA binding |  | 1.3e-03 | 0.33 |  |
|  | Gain of Loop |  | 2.6e-03 | 0.32 |  |
|  | Loss of Allosteric site at R912 |  | 0.04 | 0.21 |  |
|  | Gain of Methylation at K907 |  | 0.03 | 0.12 |  |
|  | Altered Transmembrane protein |  | 0.04 | 0.11 |  |
|  | Loss of Catalytic site at R912 |  | 0.04 | 0.09 |  |
|  | Loss of Relative solvent accessibility |  | 7.6e-03 | 0.31 |  |
|  | Altered metal binding |  | 0.02 | 0.29 |  |
|  | Gain of Acetylation at K889 |  | 0.03 | 0.21 |  |
|  | Altered Disordered interface |  | 0.04 | 0.17 |  |
|  | Gain of Catalytic site at K889 |  | 0.04 | 0.10 |  |
|  | Loss of Methylation at K889 |  | 0.05 | 0.10 |  |
|  | Altered DNA binding |  | 0.03 | 0.19 |  |
|  | Altered metal binding |  | 0.05 | 0.16 |  |
| F893L | Altered ordered interface | 0.922 | 1.0e-02 | 0.33 | ELME000233 |
|  | Loss of Allosteric site at L895 |  | 5.6e-03 | 0.29 |  |
|  | Altered metal binding |  | 6.3e-03 | 0.28 |  |
|  | Loss of Relative solvent accessibility |  | 0.02 | 0.28 |  |
|  | Loss of Catalytic site at D892 |  | 7.1e-03 | 0.23 |  |
|  | Loss of Acetylation at K889 |  | 0.04 | 0.21 |  |
|  | Altered DNA binding |  | 0.03 | 0.18 |  |
|  | Gain of Methylation at K889 |  | 0.03 | 0.11 |  |
|  | Altered Transmembrane protein |  | 0.04 | 0.10 |  |
|  | Altered ordered interface |  | 0.03 | 0.24 |  |
| R721G | Loss of Helix | 0.930 | 0.02 | 0.29 | ELME000102 |
|  | Gain of Acetylation at K722 |  | 6.6e-03 | 0.27 |  |
| E805K | Gain of Acetylation at K808 | 0.914 | 0.02 | 0.29 | ELME000047, ELME000146, ELME000333 |
|  |  |  | 4.5e-03 |  |  |
| E734K | Gain of Acetylation at E734 | 0.942 | 7.5e-04 | 0.45 | PS00107 |
|  | Loss of Catalytic site at E732 |  | 1.8e-03 | 0.30 |  |
|  | Gain of Relative solvent accessibility |  | 0.02 | 0.28 |  |
|  | Altered DNA binding |  | 0.03 | 0.18 |  |
|  | Loss of Methylation at K737 |  | 0.02 | 0.13 |  |
| A756D | Gain of Relative solvent accessibility | 0.933 | 2.2e-03 | 0.34 | ELME000052, ELME000220, PS00006, PS00107 |
|  | Altered ordered interface |  | 0.02 | 0.29 |  |
|  | Gain of Strand |  | 0.02 | 0.27 |  |
|  | Gain of Allosteric site at K758 |  | 6.4e-03 | 0.27 |  |
|  | Gain of Acetylation at K758 |  | 7.3e-03 | 0.27 |  |
|  | Loss of Methylation at K761 |  | 1.7e-03 | 0.26 |  |
|  | Altered DNA binding |  | 7.7e-03 | 0.24 |  |
|  | Altered metal binding |  | 0.04 | 0.11 |  |

**Table S2 |** Prediction of Phosphorylation sites in wild-type and mutant-type RET using NetPhos 3.1.

| Netphos 3.1 | Wild type residues | | | Netphos 3.1 | Mutant type residues | | |
| --- | --- | --- | --- | --- | --- | --- | --- |
| Serine (S) | **Position** | **Score** | **Kinase** | **Serine (S)** | **Position** | **Score** | **Kinase** |
|  | 6 S | 0.738 | unsp |  | 6 S | 0.738 | unsp |
|  | 6 S | 0.519 | cdc2 |  | 6 S | 0.519 | cdc2 |
|  | 32 S | 0.579 | CKI |  | 32 S | 0.579 | CKI |
|  | 104 S | 0.976 | unsp |  | 104 S | 0.976 | unsp |
|  | 104 S | 0.532 | CKII |  | 104 S | 0.532 | CKII |
|  | 105 S | 0.960 | unsp |  | 105 S | 0.960 | unsp |
|  | 110 S | 0.812 | unsp |  | 110 S | 0.812 | unsp |
|  | 110 S | 0.807 | PKC |  | 110 S | 0.807 | PKC |
|  | 128 S | 0.757 | unsp |  | 128 S | 0.757 | unsp |
|  | 128 S | 0.546 | p38MAPK |  | 128 S | 0.546 | p38MAPK |
|  | 131 S | 0.997 | unsp |  | 131 S | 0.997 | unsp |
|  | 148 S | 0.663 | PKC |  | 148 S | 0.663 | PKC |
|  | 159 S | 0.862 | PKC |  | 159 S | 0.862 | PKC |
|  | 173 S | 0.962 | unsp |  | 173 S | 0.962 | unsp |
|  | 220 S | 0.974 | unsp |  | 220 S | 0.974 | unsp |
|  | 224 S | 0.951 | unsp |  | 224 S | 0.951 | unsp |
|  | 307 S | 0.883 | unsp |  | 307 S | 0.883 | unsp |
|  | 316 S | 0.635 | PKA |  | 316 S | 0.635 | PKA |
|  | 316 S | 0.529 | RSK |  | 316 S | 0.529 | RSK |
|  | 345 S | 0.684 | PKC |  | 345 S | 0.684 | PKC |
|  | 345 S | 0.519 | cdc2 |  | 345 S | 0.519 | cdc2 |
|  | 363 S | 0.996 | unsp |  | 363 S | 0.996 | unsp |
|  | 363 S | 0.641 | PKA |  | 363 S | 0.641 | PKA |
|  | 363 S | 0.528 | cdc2 |  | 363 S | 0.528 | cdc2 |
|  | 365 S | 0.954 | unsp |  | 365 S | 0.954 | unsp |
|  | 396 S | 0.593 | PKC |  | 396 S | 0.593 | PKC |
|  | 406 S | 0.529 | cdc2 |  | 406 S | 0.529 | cdc2 |
|  | 409 S | 0.544 | cdc2 |  | 409 S | 0.544 | cdc2 |
|  | 411 S | 0.556 | PKC |  | 411 S | 0.556 | PKC |
|  | 411 S | 0.540 | cdc2 |  | 411 S | 0.540 | cdc2 |
|  | 413 S | 0.913 | unsp |  | 413 S | 0.913 | unsp |
|  | 413 S | 0.666 | PKC |  | 413 S | 0.666 | PKC |
|  | 434 S | 0.580 | PKC |  | 434 S | 0.580 | PKC |
|  | 444 S | 0.695 | unsp |  | 444 S | 0.695 | unsp |
|  | 444 S | 0.642 | PKC |  | 444 S | 0.642 | PKC |
|  | 445 S | 0.548 | PKA |  | 445 S | 0.548 | PKA |
|  | 457 S | 0.993 | unsp |  | 457 S | 0.993 | unsp |
|  | 457 S | 0.581 | CKI |  | 457 S | 0.581 | CKI |
|  | 493 S | 0.822 | unsp |  | 493 S | 0.822 | unsp |
|  | 507 S | 0.536 | CKII |  | 507 S | 0.536 | CKII |
|  | 518 S | 0.646 | PKC |  | 518 S | 0.646 | PKC |
|  | 522 S | 0.975 | unsp |  | 522 S | 0.975 | unsp |
|  | 522 S | 0.720 | PKC |  | 522 S | 0.720 | PKC |
|  | 536 S | 0.574 | p38MAPK |  | 536 S | 0.574 | p38MAPK |
|  | 536 S | 0.572 | CKI |  | 536 S | 0.572 | CKI |
|  | 556 S | 0.989 | unsp |  | 556 S | 0.989 | unsp |
|  | 559 S | 0.535 | cdk5 |  | 559 S | 0.535 | cdk5 |
|  | 561 S | 0.887 | unsp |  | 561 S | 0.887 | unsp |
|  | 561 S | 0.503 | PKC |  | 561 S | 0.503 | PKC |
|  | 589 S | 0.804 | unsp |  | 589 S | 0.804 | unsp |
|  | 589 S | 0.509 | PKA |  | 589 S | 0.509 | PKA |
|  | 645 S | 0.680 | PKC |  | 645 S | 0.680 | PKC |
|  | 653 S | 0.556 | PKC |  | 653 S | 0.556 | PKC |
|  | 670 S | 0.557 | CKII |  | 670 S | 0.557 | CKII |
|  | 670 S | 0.505 | PKA |  | 670 S | 0.505 | PKA |
|  | 671 S | 0.931 | unsp |  | 671 S | 0.931 | unsp |
|  | 686 S | 0.544 | cdc2 |  | 686 S | 0.544 | cdc2 |
|  | 688 S | 0.734 | unsp |  | 688 S | 0.734 | unsp |
|  | 688 S | 0.533 | cdc2 |  | 688 S | 0.533 | cdc2 |
|  | 689 S | 0.794 | unsp |  | 689 S | 0.794 | unsp |
|  | 690 S | 0.811 | PKC |  | 690 S | 0.811 | PKC |
|  | 696 S | 0.995 | unsp |  | 696 S | 0.995 | unsp |
|  | 696 S | 0.872 | PKA |  | 696 S | 0.872 | PKA |
|  | 696 S | 0.541 | RSK |  | 696 S | 0.541 | RSK |
|  | 696 S | 0.514 | cdc2 |  | 696 S | 0.514 | cdc2 |
|  | 699 S | 0.996 | unsp |  | 699 S | 0.996 | unsp |
|  | 705 S | 0.551 | PKC |  | 705 S | 0.551 | PKC |
|  | 705 S | 0.543 | unsp |  | 705 S | 0.543 | unsp |
|  | 765 S | 0.995 | unsp |  | 765 S | 0.995 | unsp |
|  | 767 S | 0.729 | unsp |  | 767 S | 0.729 | unsp |
|  | 774 S | 0.549 | CKI |  | 774 S | 0.549 | CKI |
|  | 795 S | 0.579 | DNAPK |  | 795 S | 0.579 | DNAPK |
|  | 811 S | 0.638 | PKC |  | 811 S | 0.638 | PKC |
|  | 819 S | 0.995 | unsp |  | 819 S | 0.995 | unsp |
|  | 819 S | 0.611 | PKA |  | 819 S | 0.611 | PKA |
|  | 819 S | 0.537 | cdc2 |  | 819 S | 0.537 | cdc2 |
|  | 819 S | 0.514 | PKG |  | 819 S | 0.514 | PKG |
|  | 829 S | 0.645 | PKC |  | 829 S | 0.645 | PKC |
|  | 832 S | 0.528 | PKC |  | 832 S | 0.528 | PKC |
|  | 835 S | 0.907 | unsp |  | 835 S | 0.907 | unsp |
|  | 835 S | 0.540 | PKA |  | 835 S | 0.540 | PKA |
|  | 835 S | 0.519 | cdc2 |  | 835 S | 0.519 | cdc2 |
|  | 836 S | 0.997 | unsp |  | 836 S | 0.997 | unsp |
|  | 836 S | 0.511 | RSK |  | 836 S | 0.511 | RSK |
|  | 837 S | 0.683 | unsp |  | 837 S | 0.683 | unsp |
|  | 837 S | 0.508 | DNAPK |  | 837 S | 0.508 | DNAPK |
|  | 837 S | 0.507 | cdc2 |  | 837 S | 0.507 | cdc2 |
|  | 853 S | 0.557 | CKI |  | 853 S | 0.557 | CKI |
|  | 859 S | 0.550 | ATM |  | 859 S | 0.550 | ATM |
|  | 859 S | 0.510 | DNAPK |  | 859 S | 0.510 | DNAPK |
|  | 891 S | 0.584 | PKA |  | 891 S | 0.584 | PKA |
|  | 896 S | 0.559 | CKII |  | 896 S | 0.568 | ATM |
|  | **904 S** | **0.519** | **cdc2** |  | 896 S | 0.559 | DNAPK |
|  | 909 S | 0.987 | unsp |  | 896 S | 0.540 | CKII |
|  | 909 S | 0.596 | ATM |  | 909 S | 0.987 | unsp |
|  | 909 S | 0.579 | PKC |  | 909 S | 0.596 | ATM |
|  | 909 S | 0.507 | DNAPK |  | 909 S | 0.579 | PKC |
|  | 977 S | 0.945 | unsp |  | 909 S | 0.507 | DNAPK |
|  | 977 S | 0.533 | CKII |  | 977 S | 0.945 | unsp |
|  | 1002 S | 0.529 | CKII |  | 977 S | 0.533 | CKII |
|  | 1021 S | 0.521 | cdc2 |  | 1002 S | 0.529 | CKII |
|  | 1026 S | 0.869 | unsp |  | 1021 S | 0.521 | cdc2 |
|  | 1034 S | 0.997 | unsp |  | 1026 S | 0.869 | unsp |
|  | 1034 S | 0.670 | CKII |  | 1034 S | 0.997 | unsp |
|  | 1065 S | 0.764 | PKA |  | 1034 S | 0.670 | CKII |
|  | 1094 S | 0.834 | unsp |  | 1065 S | 0.764 | PKA |
|  | 1102 S | 0.928 | unsp |  | 1094 S | 0.834 | unsp |
|  |  | | |  | 1102 S | 0.928 | unsp |
|  |  |  |  | **Threonine (T)** |  |  |  |
| Threonine (T) | 5 T | 0.707 | PKC |  | 5 T | 0.707 | PKC |
|  | 48 T | 0.579 | p38MAPK |  | 48 T | 0.579 | p38MAPK |
|  | 75 T | 0.866 | PKC |  | 75 T | 0.866 | PKC |
|  | 120 T | 0.576 | PKC |  | 120 T | 0.576 | PKC |
|  | 120 T | 0.539 | cdc2 |  | 120 T | 0.539 | cdc2 |
|  | 152 T | 0.783 | unsp |  | 152 T | 0.783 | unsp |
|  | 184 T | 0.716 | PKC |  | 184 T | 0.716 | PKC |
|  | 261 T | 0.739 | unsp |  | 261 T | 0.739 | unsp |
|  | 291 T | 0.952 | unsp |  | 291 T | 0.952 | unsp |
|  | 295 T | 0.770 | PKC |  | 295 T | 0.770 | PKC |
|  | 295 T | 0.757 | unsp |  | 295 T | 0.757 | unsp |
|  | 315 T | 0.880 | unsp |  | 315 T | 0.880 | unsp |
|  | 315 T | 0.855 | PKA |  | 315 T | 0.855 | PKA |
|  | 315 T | 0.534 | RSK |  | 315 T | 0.534 | RSK |
|  | 328 T | 0.984 | unsp |  | 328 T | 0.984 | unsp |
|  | 328T | 0.861 | PKC |  | 328T | 0.861 | PKC |
|  | 338 T | 0.677 | unsp |  | 338 T | 0.677 | unsp |
|  | 350 T | 0.604 | unsp |  | 350 T | 0.604 | unsp |
|  | 350 T | 0.529 | PKC |  | 350 T | 0.529 | PKC |
|  | 407 T | 0.589 | PKC |  | 407 T | 0.589 | PKC |
|  | 456 T | 0.655 | CKII |  | 456 T | 0.655 | CKII |
|  | 456 T | 0.558 | unsp |  | 456 T | 0.558 | unsp |
|  | 461 T | 0.784 | unsp |  | 461 T | 0.784 | unsp |
|  | 492 T | 0.930 | unsp |  | 492 T | 0.930 | unsp |
|  | 492 T | 0.858 | PKC |  | 492 T | 0.858 | PKC |
|  | 552 T | 0.524 | cdc2 |  | 552 T | 0.524 | cdc2 |
|  | 562 T | 0.524 | PKC |  | 562 T | 0.524 | PKC |
|  | 575 T | 0.581 | DNAPK |  | 575 T | 0.581 | DNAPK |
|  | 575 T | 0.572 | ATM |  | 575 T | 0.572 | ATM |
|  | 575 T | 0.508 | CKII |  | 575 T | 0.508 | CKII |
|  | 608 T | 0.509 | PKC |  | 608 T | 0.509 | PKC |
|  | 675 T | 0.961 | unsp |  | 675 T | 0.961 | unsp |
|  | 675 T | 0.640 | PKC |  | 675 T | 0.640 | PKC |
|  | 753 T | 0.655 | PKC |  | 753 T | 0.655 | PKC |
|  | 754 T | 0.688 | PKC |  | 754 T | 0.696 | unsp |
|  | 847 T | 0.878 | unsp |  | 754 T | 0.616 | PKC |
|  | 930 T | 0.772 | unsp |  | 847 T | 0.878 | unsp |
|  | 930 T | 0.512 | DNAPK |  | **918 T** | **0.644** | **PKG** |
|  | 946 T | 0.541 | CKI |  | 930 T | 0.772 | unsp |
|  | 966 T | 0.571 | PKC |  | 930 T | 0.512 | DNAPK |
|  | 1022 T | 0.907 | unsp |  | 946 T | 0.541 | CKI |
|  | 1038 T | 0.917 | unsp |  | 966 T | 0.571 | PKC |
|  | 1055 T | 0.650 | PKC |  | 1022 T | 0.907 | unsp |
|  | 1055 T | 0.510 | CKII |  | 1038 T | 0.917 | unsp |
|  | 1083 T | 0.669 | PKC |  | 1055 T | 0.650 | PKC |
|  | 1085 T | 0.779 | unsp |  | 1055 T | 0.510 | CKII |
|  |  | | |  | 1083 T | 0.669 | PKC |
|  |  |  |  |  | 1085 T | 0.779 | unsp |
|  |  |  |  | **Tyrosine (Y)** | 36 Y | 0.910 | unsp |
| Tyrosine (Y) | 36 Y | 0.910 | unsp |  | 41 Y | 0.972 | unsp |
|  | 41 Y | 0.972 | unsp |  | 237 Y | 0.937 | unsp |
|  | 237 Y | 0.937 | unsp |  | 314 Y | 0.500 | INSR |
|  | 314 Y | 0.500 | INSR |  | 483 Y | 0.905 | unsp |
|  | 483 Y | 0.905 | unsp |  | 508 Y | 0.882 | unsp |
|  | 508 Y | 0.882 | unsp |  | 508 Y | 0.512 | SRC |
|  | 508 Y | 0.512 | SRC |  | 606 Y | 0.512 | EGFR |
|  | 606 Y | 0.512 | EGFR |  | 687 Y | 0.732 | unsp |
|  | 687 Y | 0.732 | unsp |  | 826 Y | 0.563 | unsp |
|  | **752 Y** | **0.500** | **EGFR** |  | 900 Y | 0.891 | unsp |
|  | 826 Y | 0.563 | unsp |  | 905 Y | 0.989 | unsp |
|  | 900 Y | 0.891 | unsp |  | 905 Y | 0.531 | INSR |
|  | 905 Y | 0.989 | unsp |  | 905 Y | 0.525 | SRC |
|  | 905 Y | 0.531 | INSR |  | 905 Y | 0.514 | EGFR |
|  | 905 Y | 0.525 | SRC |  | 928 Y | 0.595 | EGFR |
|  | 905 Y | 0.514 | EGFR |  | 981 Y | 0.910 | unsp |
|  | 928 Y | 0.595 | EGFR |  | 1015 Y | 0.777 | unsp |
|  | 981 Y | 0.910 | unsp |  | 1029 Y | 0.723 | unsp |
|  | 1015 Y | 0.777 | unsp |  | 1062 Y | 0.680 | unsp |
|  | 1029 Y | 0.723 | unsp |  | 1062 Y | 0.506 | SRC |
|  | 1062 Y | 0.680 | unsp |  | 1096 Y | 0.821 | unsp |
|  | 1062 Y | 0.506 | SRC |  | 1096 Y | 0.507 | SRC |
|  | 1096 Y | 0.821 | unsp |  |  | | |
|  | 1096 Y | 0.507 | SRC |  |  |  |  |

**Table S3 |** Functions of proteins connected with *RET* from STRING database*.*

| Serial no | Protein name | Protein function |
| --- | --- | --- |
| 01 | GDNF family receptor alpha-3 (GFRA3) | - Mediates the artemin-induced autophosphorylation and activation of the RET receptor tyrosine kinase. |
| 02 | Neurturin (NRTN) | - Supports the survival of sympathetic neurons in culture. - May regulate the development and maintenance of the CNS. - Might control the size of non-neuronal cell population such as haemopoietic cells. |
| 03 | GDNF family receptor alpha-1 (GFRA1) | - Mediates the GDNF-induced autophosphorylation and activation of the RET receptor. |
| 04 | Artemin (ARTN) | - Supports the survival of sensory and sympathetic peripheral neurons in culture. - Supports the survival of dopaminergic neurons of the ventral mid-brain. |
| 05 | Glial cell line-derived neurotrophic factor (GDNF) | - Enhances survival and morphological differentiation of dopaminergic neurons and increases their high- affinity dopamine uptake. |
| 06 | GDNF family receptor alpha-2 (GFRA2) | - Mediates the NRTN-induced autophosphorylation and activation of the RET receptor. - Able to mediate GDNF signaling through the RET tyrosine kinase receptor. |
| 07 | GDNF family receptor alpha-like (GFRAL) | - Regulates food intake, energy expenditure and body weight in response to metabolic and toxin-induced stresses. - Interacts with RET and induces cellular signaling through activation of MAPK- and AKT- signaling pathways. |
| 08 | Persephin (PSPN) | - Exhibits neurotrophic activity on mesencephalic dopaminergic and motor neurons. |
| 09 | Nuclear receptor coactivator 4 (NCOA4) | - Enhances the androgen receptor transcriptional activity in prostate cancer cells. |
| 10 | Coiled-coil domain containing 6 (CCDC6) | No information in server |

**Table S4 |** RET Interaction Network (IntAct-derived Proteins).

| **Accession** | **Gene Name** | **UniProt ID** | **Species** | **Description** |
| --- | --- | --- | --- | --- |
| EBI-355164 | VCP | P55072 | Homo sapiens | Transitional endoplasmic reticulum ATPase |
| EBI-491274 | RB1 | P06400 | Homo sapiens | Retinoblastoma-associated protein |
| EBI-354921 | HSPA5 | P11021 | Homo sapiens | Endoplasmic reticulum chaperone BiP |
| EBI-739467 | GORASP2 | Q9H8Y8 | Homo sapiens | Golgi reassembly-stacking protein 2 |
| EBI-368690 | APBA1 | Q02410 | Homo sapiens | Amyloid-beta A4 precursor protein-binding family A member 1 |
| EBI-6115839 | APBA3 | O96018 | Homo sapiens | Amyloid-beta A4 precursor protein-binding family A member 3 |
| EBI-80830 | NCOR2 | Q9Y618 | Homo sapiens | Nuclear receptor corepressor 2 |
| EBI-455189 | NCOA1 | Q15788 | Homo sapiens | Nuclear receptor coactivator 1 |
| EBI-1049597 | CALR | P27797 | Homo sapiens | Calreticulin |
| EBI-356710 | RCN2 | Q14257 | Homo sapiens | Reticulocalbin-2 |
| EBI-993903 | ERBIN | Q96RT1 | Homo sapiens | Erbin |
| EBI-948278 | RCN1 | Q15293 | Homo sapiens | Reticulocalbin-1 |
| EBI-356576 | SLC1A5 | Q15758 | Homo sapiens | Neutral amino acid transporter B(0) |
| EBI-448924 | E2F1 | Q01094 | Homo sapiens | Transcription factor E2F1 |
| EBI-9640524 | NR1H4 | Q96RI1-2 | Homo sapiens | Bile acid receptor |
| EBI-592823 | RBBP5 | Q15291 | Homo sapiens | Retinoblastoma-binding protein 5 |
| EBI-413374 | RARA | P10276 | Homo sapiens | Retinoic acid receptor alpha |
| EBI-3923320 | SLC1A7 | O00341 | Homo sapiens | Excitatory amino acid transporter 5 |
| EBI-66908608 | ATP2A2 | P16615-3 | Homo sapiens | SERCA2 isoform 3 |
| EBI-301834 | HDAC1 | Q13547 | Homo sapiens | Histone deacetylase 1 |
| EBI-1994109 | NPLOC4 | Q8TAT6 | Homo sapiens | Nuclear protein localization protein 4 |
| EBI-1994090 | UFD1 | Q92890 | Homo sapiens | ER-associated degradation protein |
| EBI-16130425 | ASH2L | Q9UBL3-3 | Homo sapiens | Histone methyltransferase complex subunit |
| EBI-16364752 | RAET1L | Q5VY80 | Homo sapiens | UL16-binding protein 6 |
| EBI-15930546 | FAF1 | Q9UNN5-1 | Homo sapiens | FAS-associated factor 1 |
| EBI-620823 | RBBP4 | Q09028 | Homo sapiens | Histone-binding protein RBBP4 |
| EBI-302023 | H4C16 | P62805 | Homo sapiens | Histone H4 |
| EBI-712001 | GABARAP | O95166 | Homo sapiens | GABA receptor-associated protein |
| EBI-540834 | WDR5 | P61964 | Homo sapiens | WD repeat-containing protein 5 |
| EBI-21776319 | HTRA4 | P83105 | Homo sapiens | Serine protease HTRA4 |
| EBI-354158 | VDAC1 | P21796 | Homo sapiens | Voltage-dependent anion channel 1 |
| EBI-80168 | UBE2I | P63279 | Homo sapiens | SUMO-conjugating enzyme UBC9 |
| EBI-349905 | BRCA1 | P38398 | Homo sapiens | Breast cancer type 1 susceptibility protein |
| EBI-352227 | RBBP7 | Q16576 | Homo sapiens | Histone-binding protein RBBP7 |
| EBI-715849 | NDC80 | O14777 | Homo sapiens | Kinetochore protein |
| EBI-347233 | NCOR1 | O75376 | Homo sapiens | Nuclear receptor corepressor 1 |
| EBI-711260 | UNC119 | Q13432 | Homo sapiens | Protein unc-119 homolog A |
| EBI-310873 | LRP4 | O75096 | Homo sapiens | LDL receptor-related protein 4 |
| EBI-396684 | EPS15 | P42566 | Homo sapiens | EGFR substrate 15 |
| EBI-2480756 | RET | P07949 | Homo sapiens | Proto-oncogene tyrosine-protein kinase receptor Ret |
| EBI-1046367 | AMFR | Q9UKV5 | Homo sapiens | E3 ubiquitin-protein ligase AMFR |
| EBI-821440 | ARHGEF12 | Q9NZN5 | Homo sapiens | Rho guanine nucleotide exchange factor 12 |
| EBI-78598 | RXRA | P19793 | Homo sapiens | Retinoic acid receptor RXR-alpha |
| EBI-5746563 | SOST | Q9BQB4 | Homo sapiens | Sclerostin |
| EBI-2462036 | TLN1 | Q9Y490 | Homo sapiens | Talin-1 |
| EBI-358919 | SEC61A1 | P61619 | Homo sapiens | Protein transport protein Sec61 |
| EBI-5325353 | MBL2 | P11226 | Homo sapiens | Mannose-binding protein C |
| EBI-712685 | PDE6D | O43924 | Homo sapiens | cGMP phosphodiesterase subunit delta |
| EBI-765739 | SPEN | Q96T58 | Homo sapiens | Msx2-interacting protein |
| EBI-721577 | NSFL1C | Q9UNZ2 | Homo sapiens | NSFL1 cofactor p47 |
| EBI-6558417 | RPGR | Q92834 | Homo sapiens | Retinitis pigmentosa GTPase regulator |
| EBI-745715 | RBBP8 | Q99708 | Homo sapiens | DNA endonuclease RBBP8 |
| EBI-308302 | TOPBP1 | Q92547 | Homo sapiens | DNA topoisomerase binding protein |
| EBI-946068 | ATXN3 | P54252-1 | Homo sapiens | Ataxin-3 |
| EBI-2556173 | MPO | P05164 | Homo sapiens | Myeloperoxidase |
| EBI-458344 | KLRK1 | P26718 | Homo sapiens | NKG2-D receptor |
| EBI-2823702 | RGP1 | Q92546 | Homo sapiens | RAB6A-GEF partner protein |
| EBI-718476 | E2F2 | Q14209 | Homo sapiens | Transcription factor E2F2 |
| EBI-10697720 | RTL10 | Q7L3V2 | Homo sapiens | Protein Bop |
| EBI-2824467 | RIC1 | Q4ADV7 | Homo sapiens | GEF subunit RIC1 |
| EBI-3942619 | ZFPM1 | Q8IX07 | Homo sapiens | Zinc finger protein |
| EBI-15986834 | TTK | P33981-1 | Homo sapiens | Dual specificity protein kinase |
| EBI-2562736 | GNAT1 | P11488 | Homo sapiens | G protein subunit alpha-1 |
| EBI-26568205 | Ficolin-2 | Q15485 | Homo sapiens | Ficolin-2 |
| EBI-27105453 | Ficolin-3 | O75636 | Homo sapiens | Ficolin-3 |
| EBI-3955784 | THRB | P10828-1 | Homo sapiens | Thyroid hormone receptor beta |
| EBI-15869194 | UBE4B | O95155-1 | Homo sapiens | Ubiquitin conjugation factor |
| EBI-1264387 | AML1-ETO | — | Homo sapiens | Fusion protein AML1-MTG8 |
| EBI-6860739 | Rheb | Q15382 | Homo sapiens | GTP-binding protein Rheb |
| EBI-20888295 | ABCA4 | P78363 | Homo sapiens | Retinal ATP-binding cassette transporter |
| EBI-16182918 | ZNF451 | Q9Y4E5-1 | Homo sapiens | SUMO E3 ligase |
| EBI-25593641 | Surface protein | Q9UQF0 | Homo sapiens | Surface protein |

**Table S5 |** Prediction of 3D model structure of *RET* protein with ribbon-presentation by using Project HOPE server.

| SNP_ID | Mutation | Modeled ribbon structure of mutated RET | Close-up view of the mutated site of RET |
| --- | --- | --- | --- |
| rs74799832 | M918T | 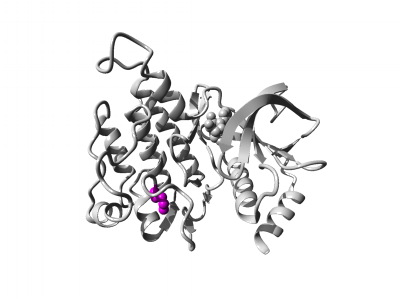 | 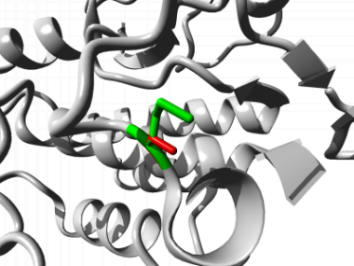 |
| rs76087194 | R897Q | 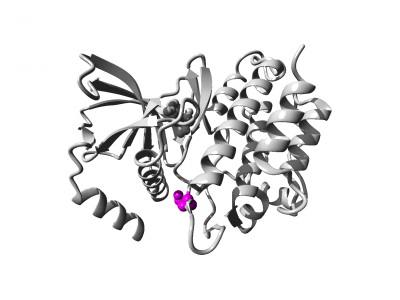 | 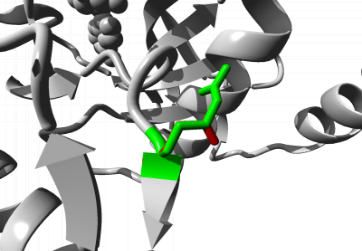 |
| rs1060500759 | R897G | 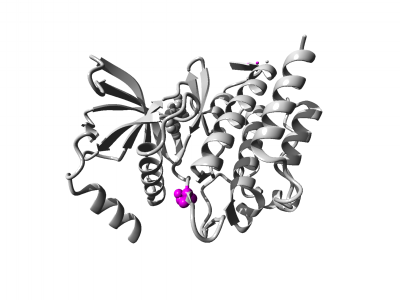 | 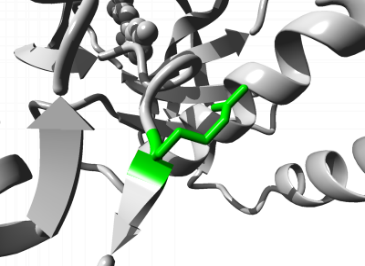 |
| rs77724903 | Y791C | 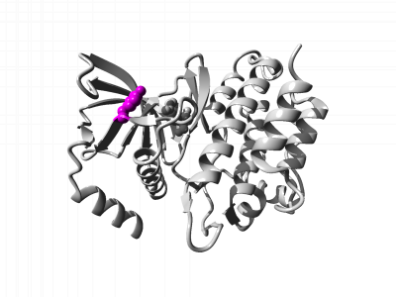 | 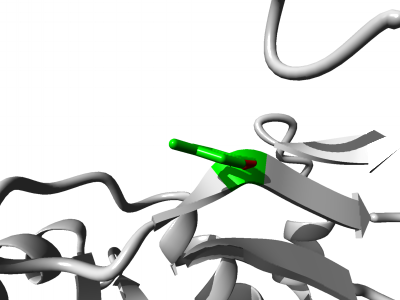 |
| rs78347871 | R912Q | 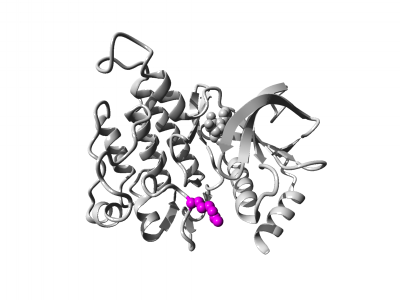 | 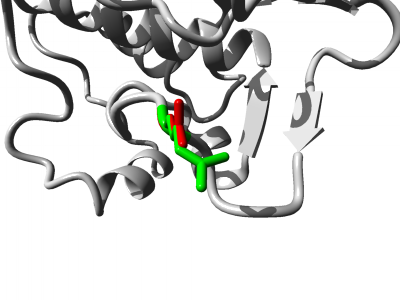 |
| rs1838227061 | R912G | **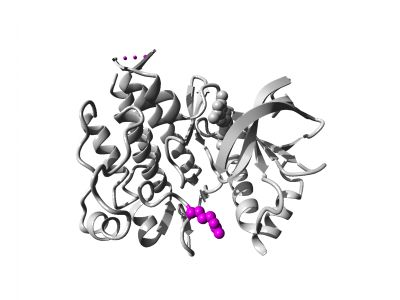** | **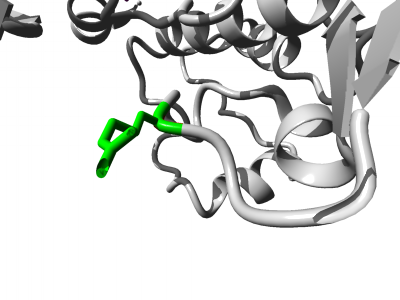** |
| rs768188546 | F893L | **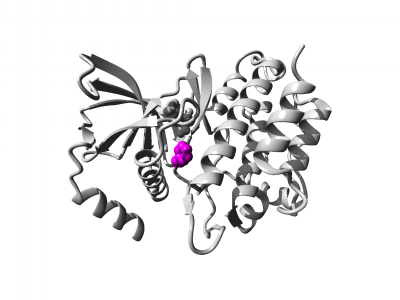** | **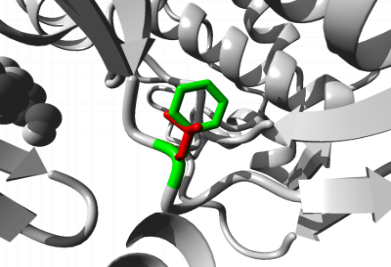** |
| rs1554819146 | R721G | **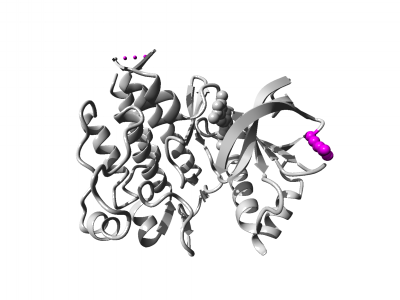** | **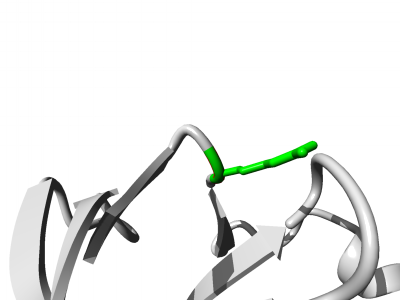** |
| rs377767418 | E805K | **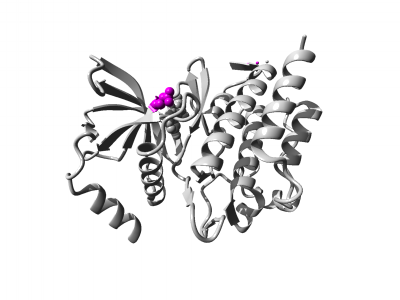** | **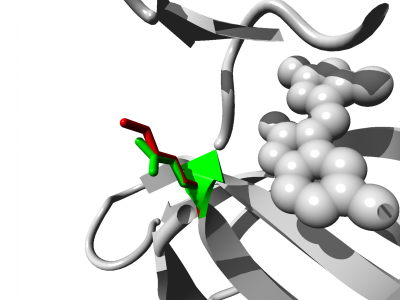** |
| rs1838077120 | E734K | **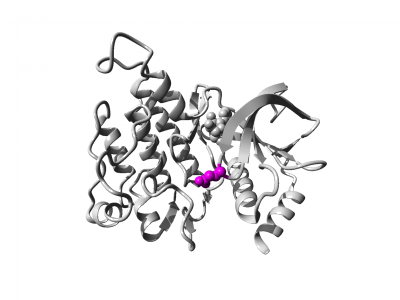** | **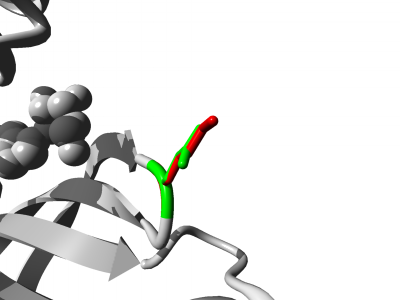** |
| rs1838079858 | A756D | **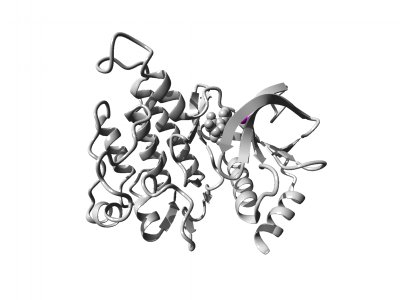** | **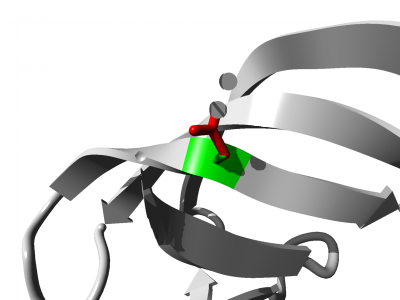** |

**3.8 Protein-Protein Interaction (PPI)** **Prediction**

The RET protein has been found to interact with a total of ten different proteins, which are as follows: GFRA3, NRTN, GFRA1, ARTN, GDNF, GFRA2, GFRAL, PSPN, NCOA4, and CCDC6. These proteins were identified by the STRING service. In addition, AIP, GRB10, CBL, SHC1, GRB2 EGFR, CBLC, STAT3, GFRA1, NRTN and GDNF were identified from BioGRID database with minimum evidence 1. Among them GFRA1, NRTN and GDNF were common between both databases (**Supplementary Figures S1, S2, Tables S3 and S4**).

The RET protein demonstrates significant connection within this network, engaging ten other proteins to create a densely interlinked structure including 11 nodes and 37 edges. This network, characterized by a PPI enrichment value of 1.05e-10 and an average node degree of 6.73, indicates significant functional linkages and potential regulatory interactions among the proteins. In addition, AIP, GRB10, CBL, SHC1, GRB2 EGFR, CBLC, STAT3, GFRA1, NRTN and GDNF were identified from BioGRID database with minimum evidence 1. Among them GFRA1, NRTN and GDNF were also identified from STRING database. After applying stringent filtering criteria, a total of 72 protein-protein interactions associated with the RET protein were identified from the IntAct database. Among these, several key interactors (BRCA1, RB1, E2F1, VCP, HDAC1 and WDR5) with established roles in cancer biology, signal transduction, cellular and transcriptional regulation were prioritized. In a nutshell, these interactions indicate that *RET* is functionally connected with key oncogenic and regulatory networks, supporting its critical role in cancer-related signaling pathways.


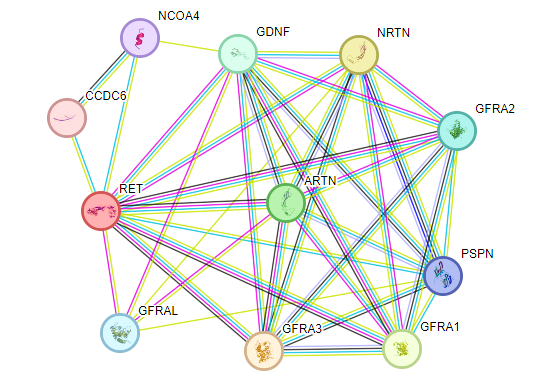


**FIGURE S1 |** Protein-protein interaction network of RET protein examined by STRING database. RET is the most connected node (highlighted in red), indicating it is a hub protein in this network. The different colored lines (edges) illustrate various types of connection of the interactions.


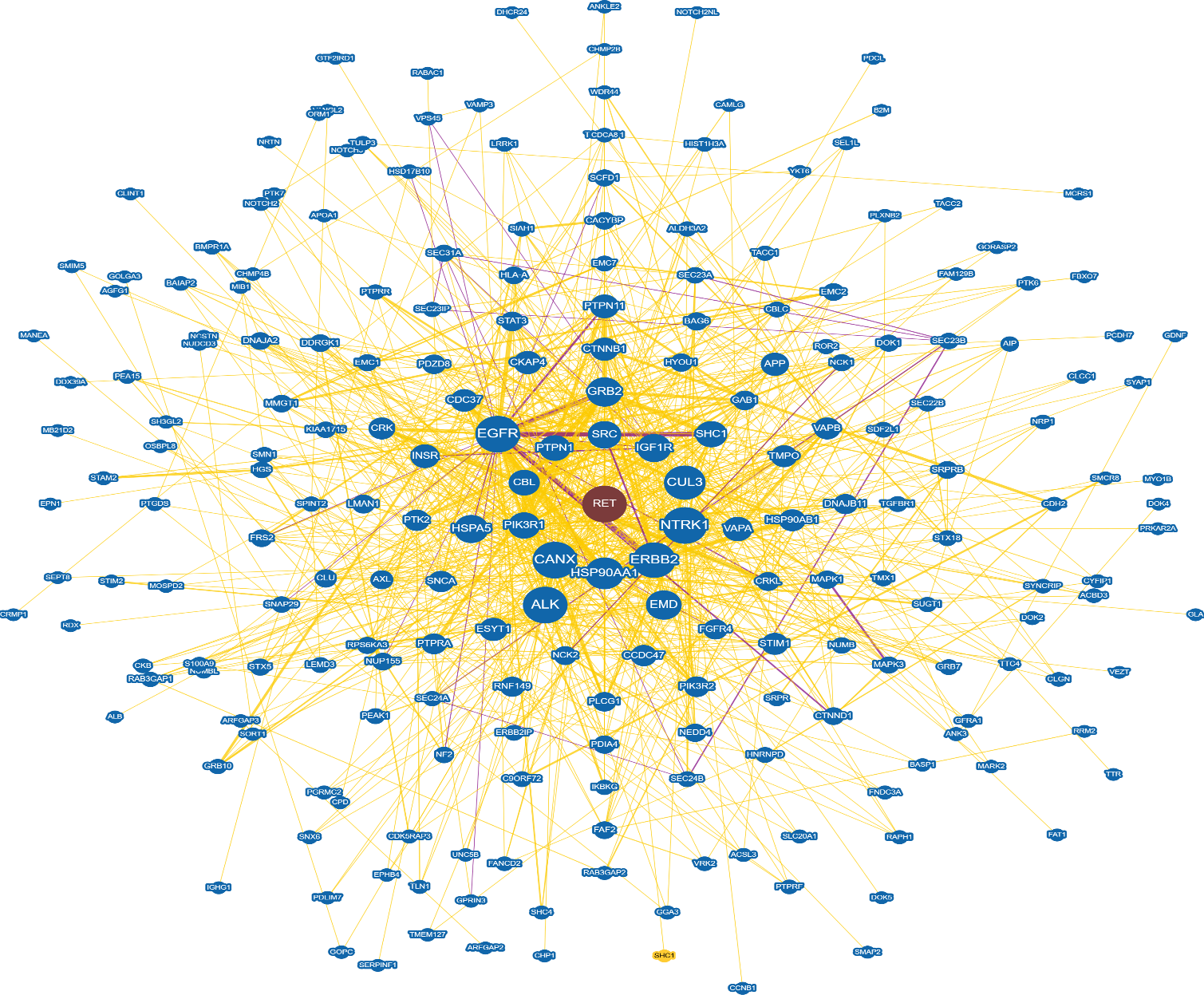


**FIGURE S2 |** Protein-protein interaction network analysis from BioGrid database. Circular shaped indicates interacted proteins and solid line indicates interaction between respective proteins.


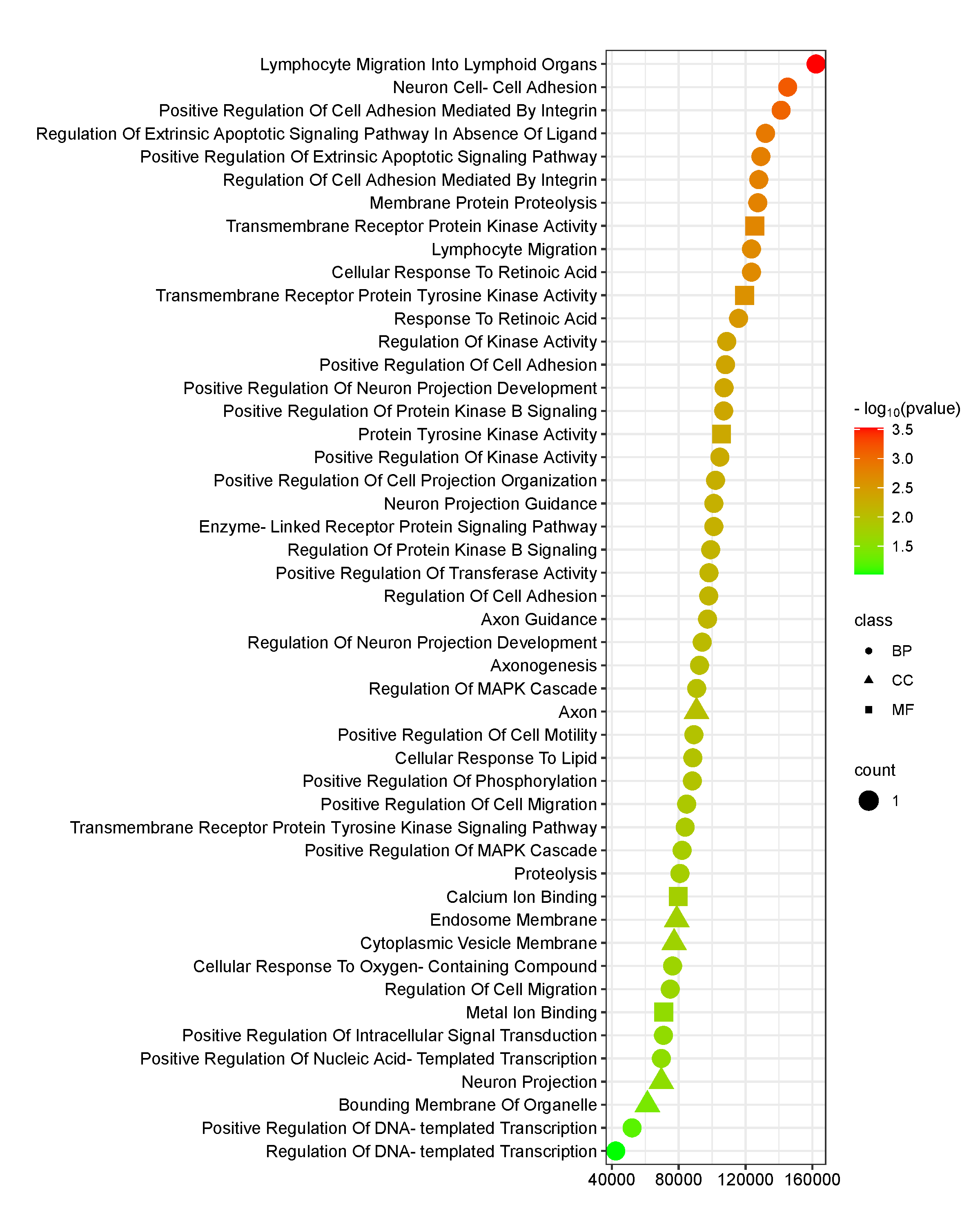


**FIGURE S3 |** Gene Ontology (GO) analysis utilizing biological process (BP), cellular component (CC), and molecular function (MF) based on gene number and -log_10_(p-value) via SRplot.

**
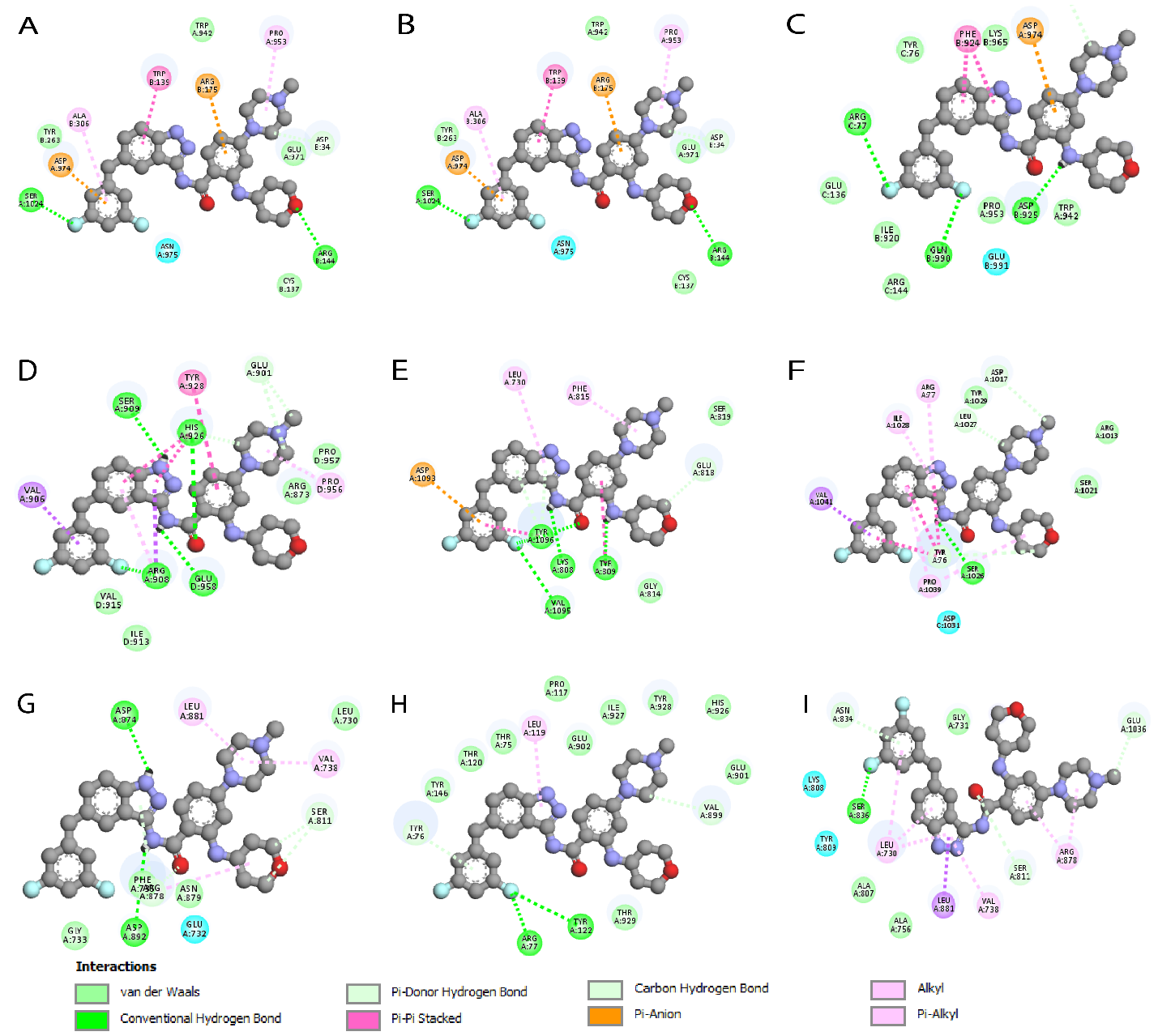
**

**FIGURE S4 |** Top molecular binding 2D interaction analysis of Entrectinib drug against seven mutant structures including (A) E734K, (B) A756D, (C) Y791C, (D) F893L, (E) R897Q, (F) M918T, (G) R897G, (H) E805K and (I) Wild type RET protein.


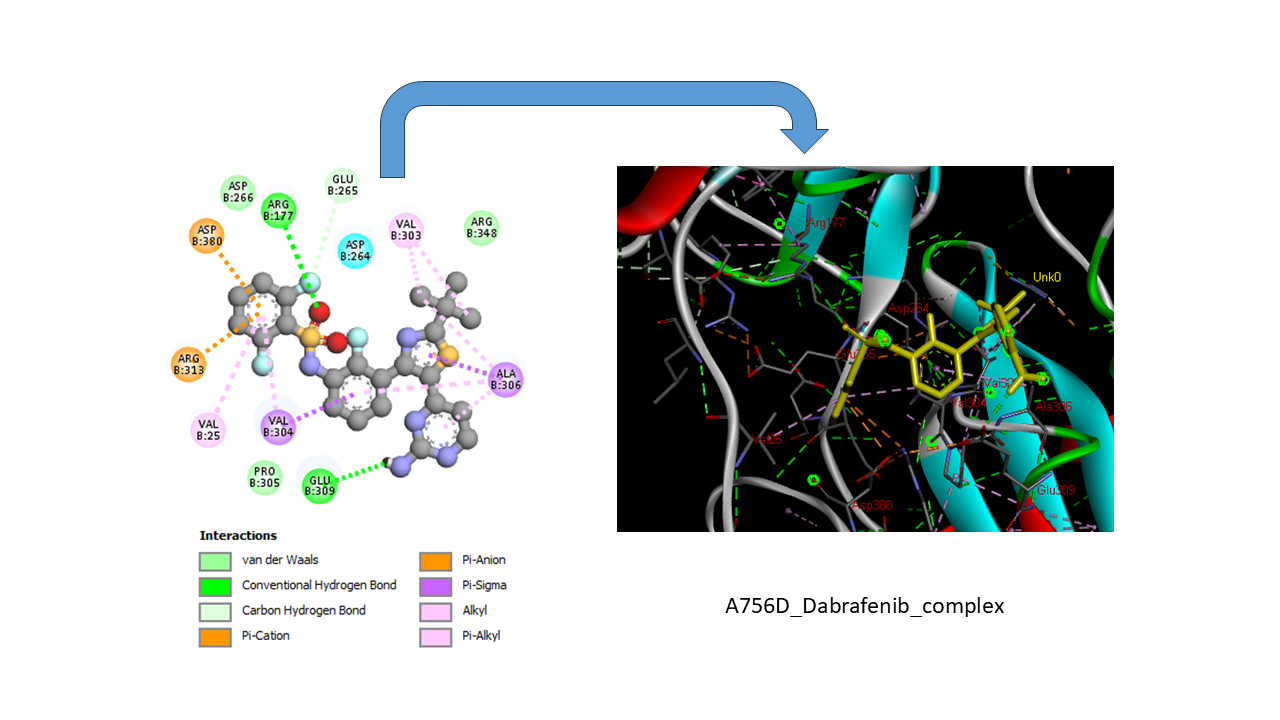


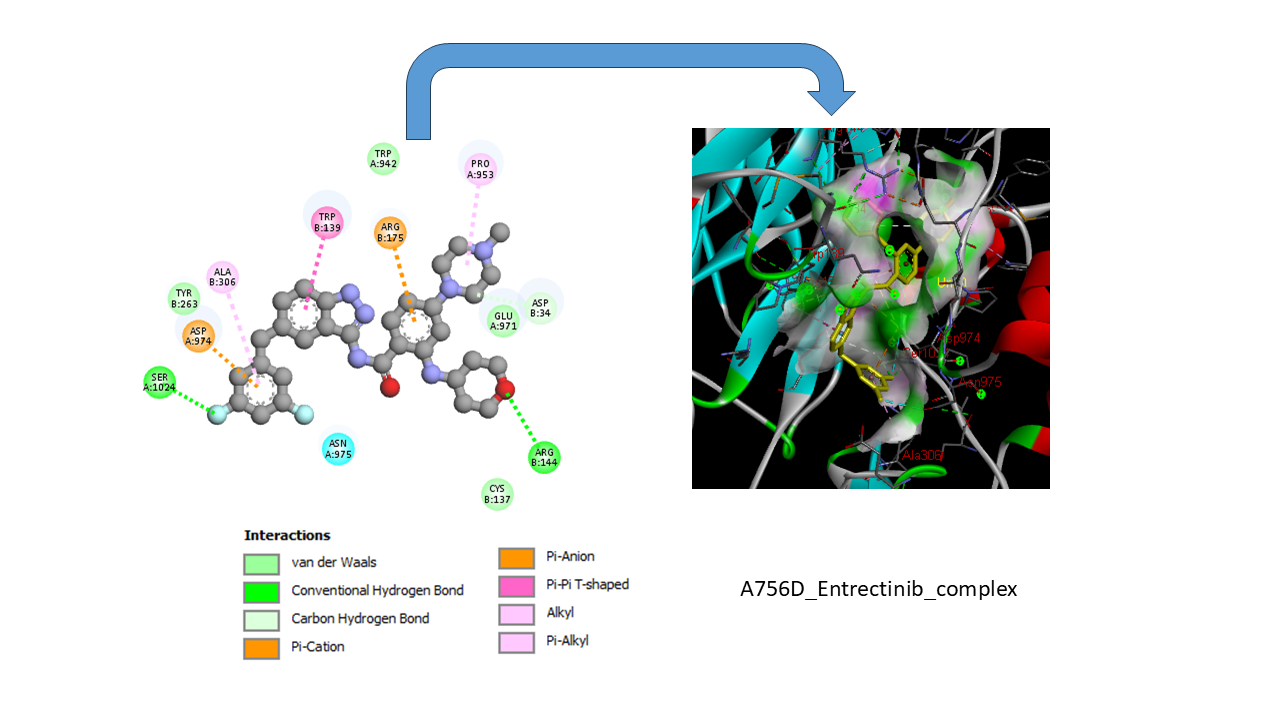


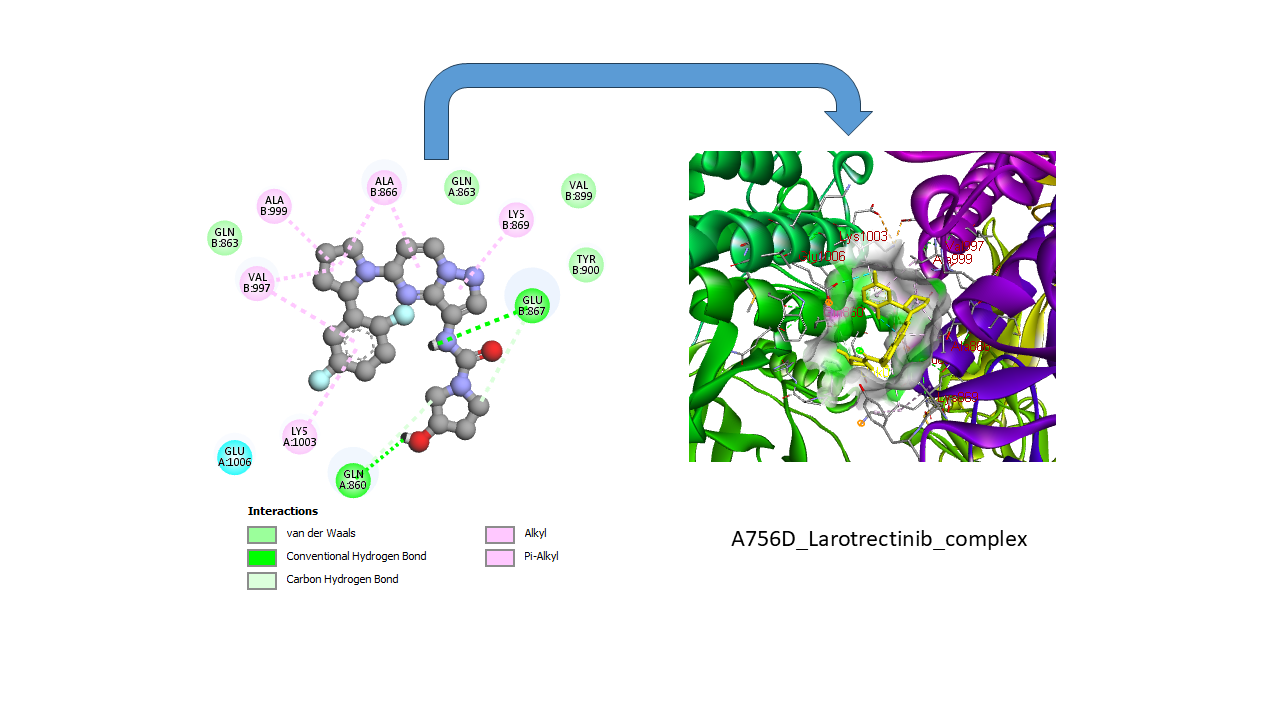


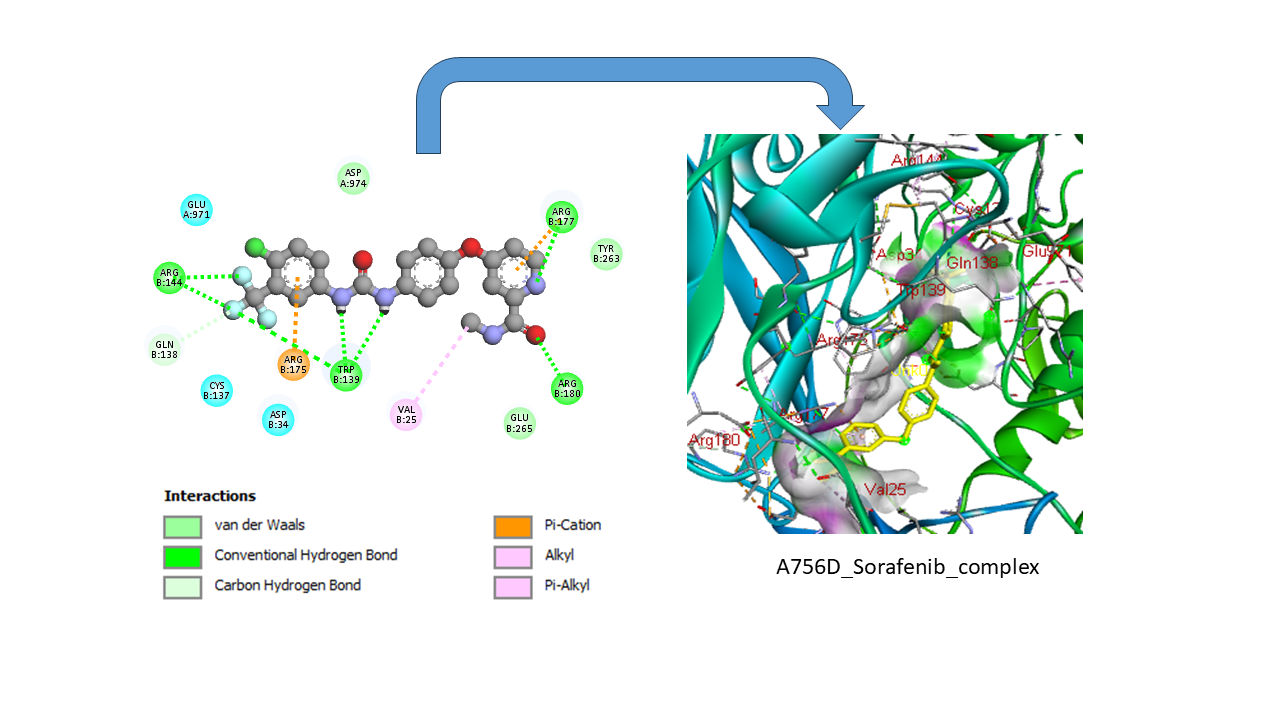


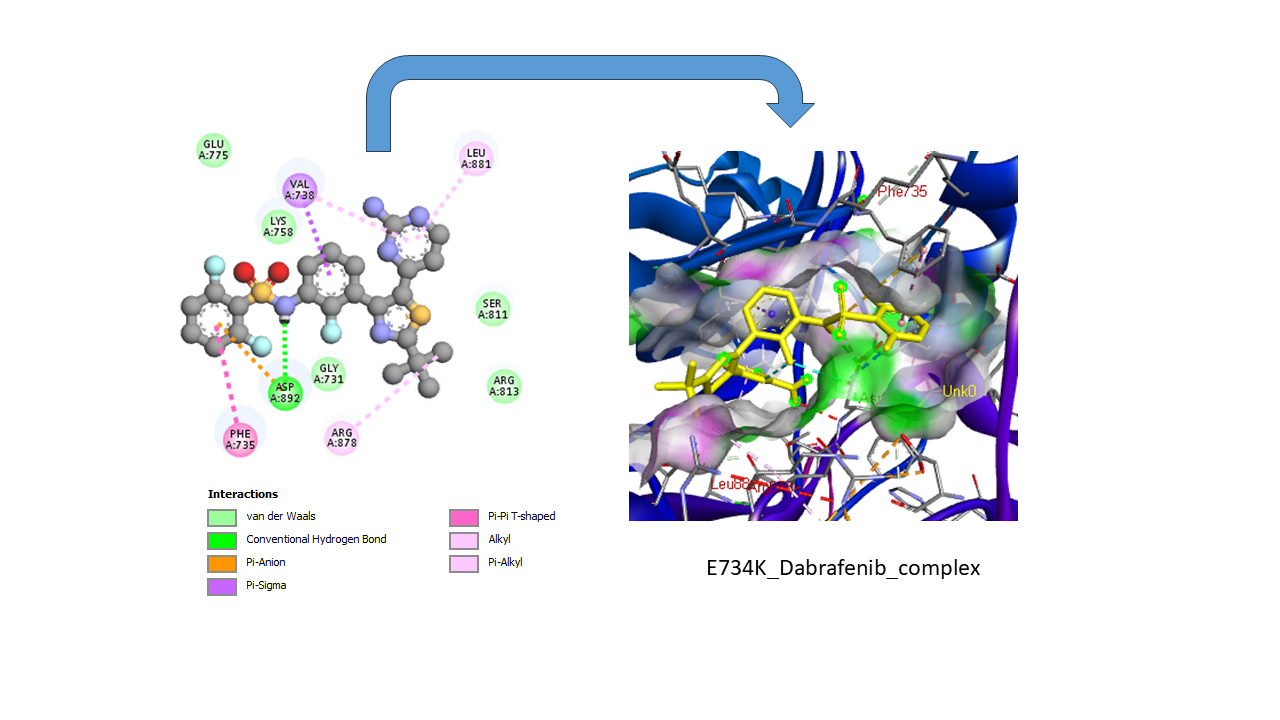


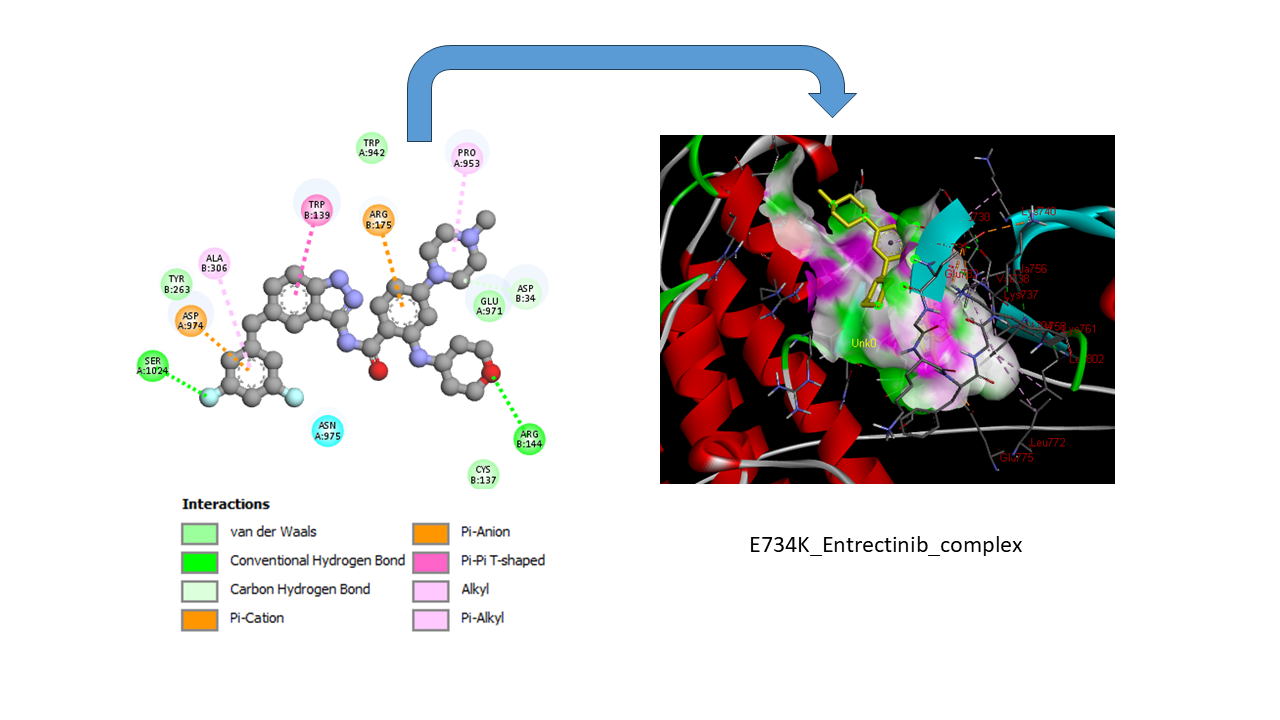


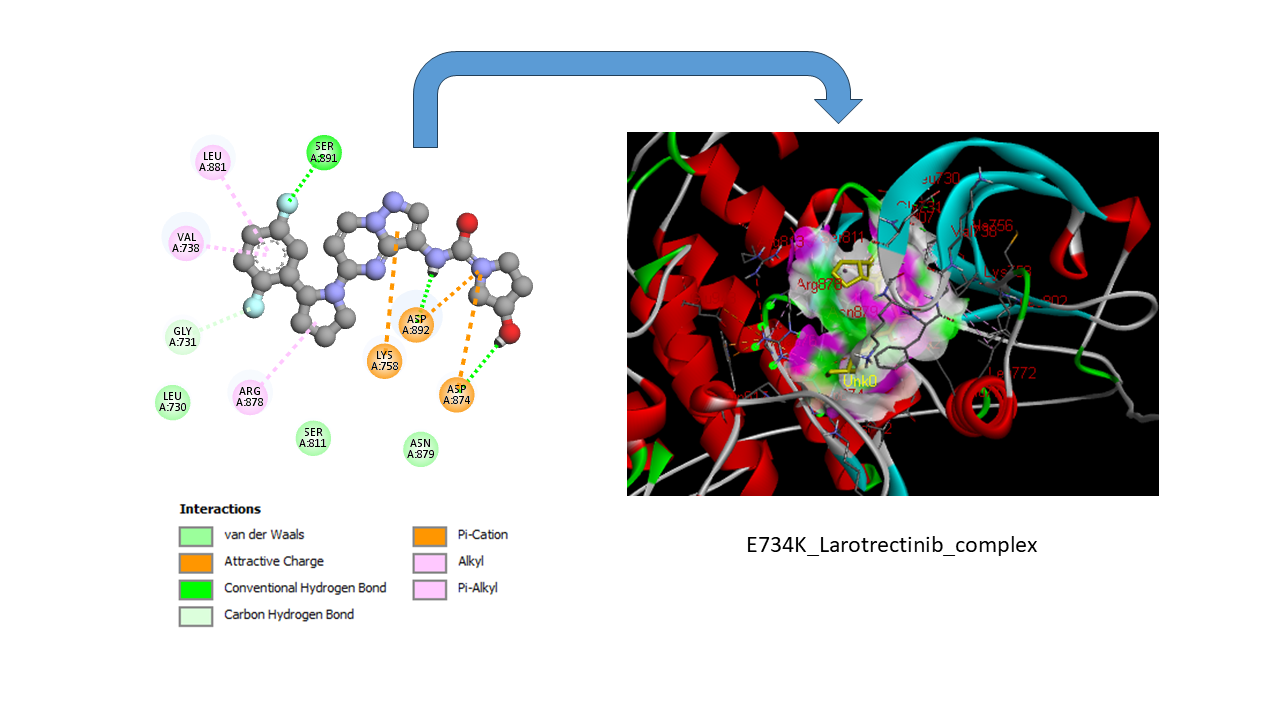


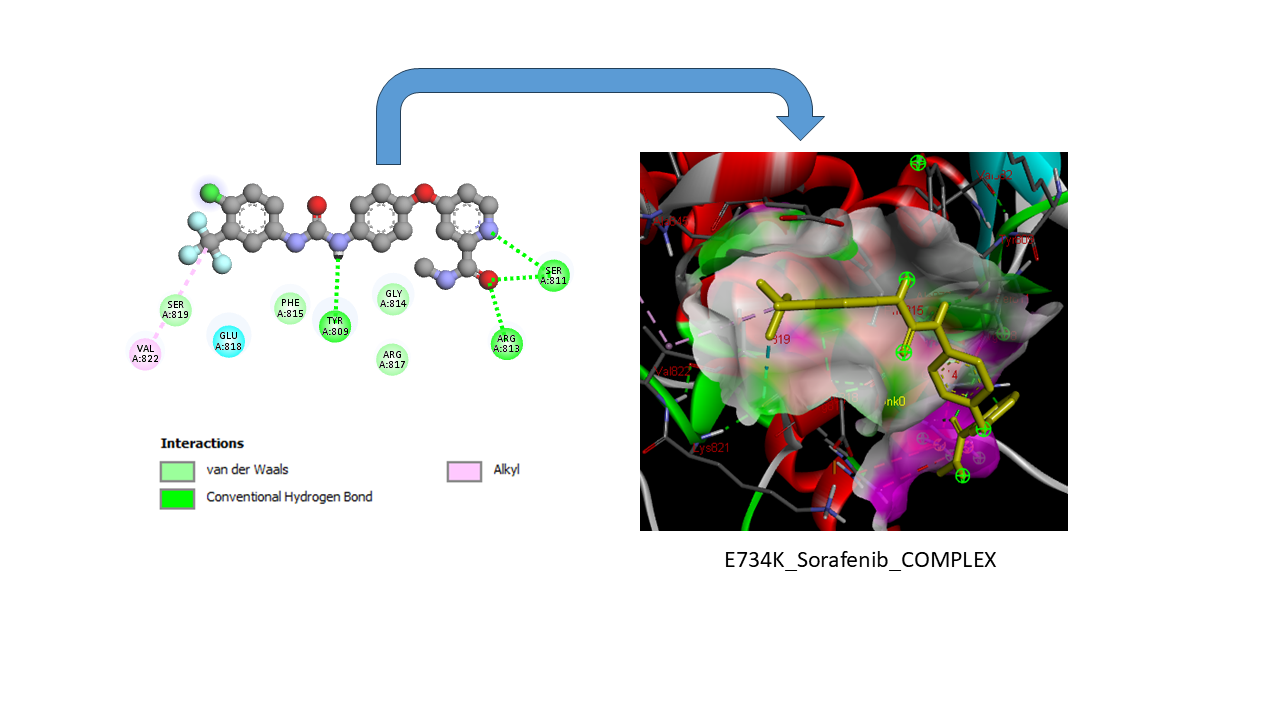


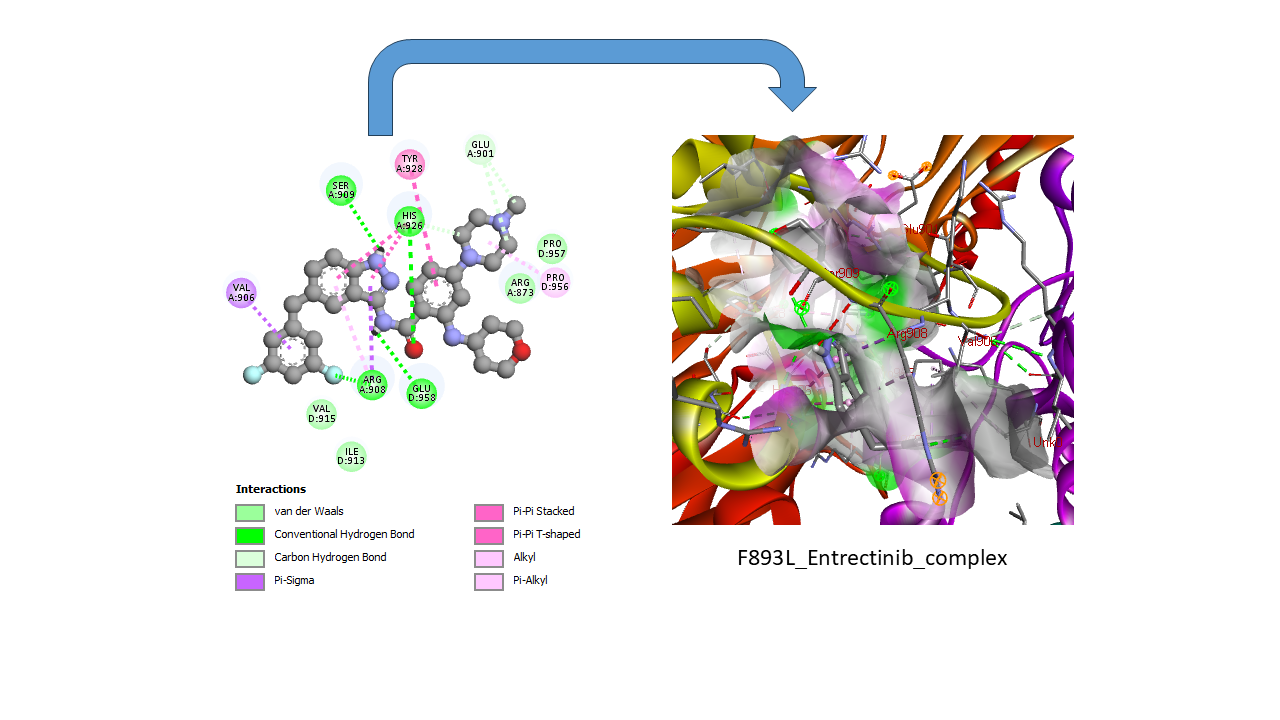

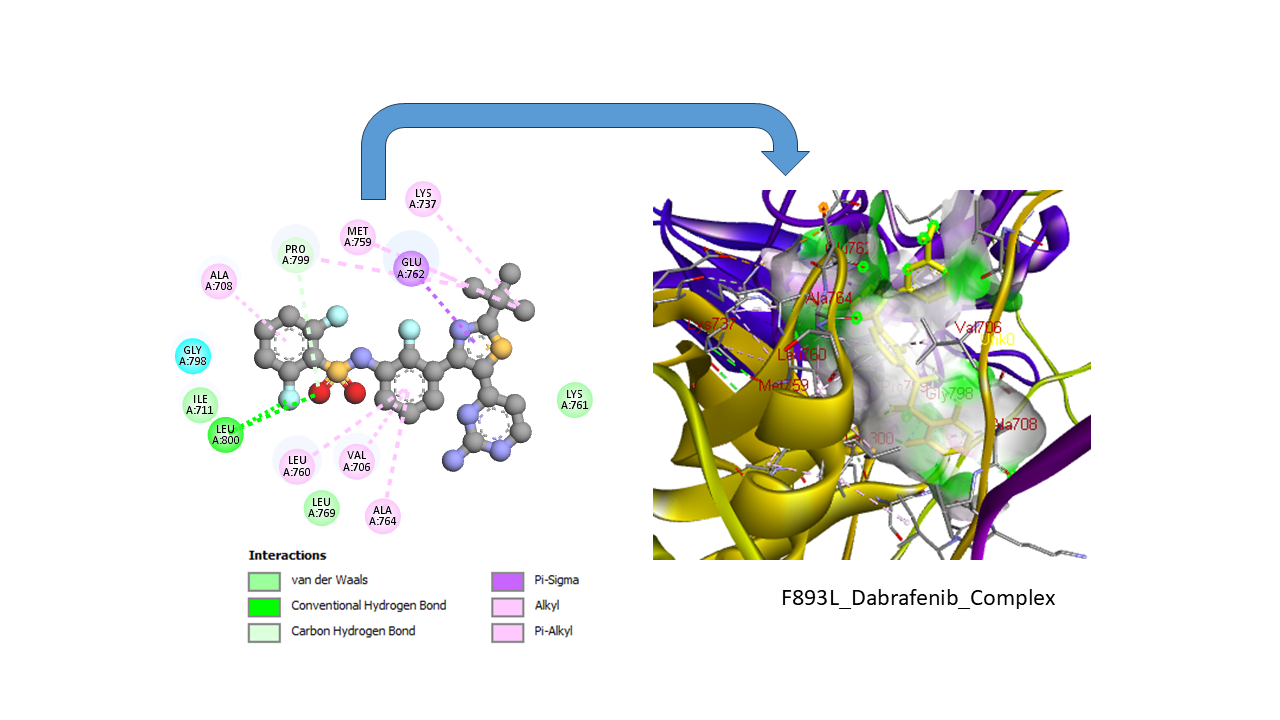


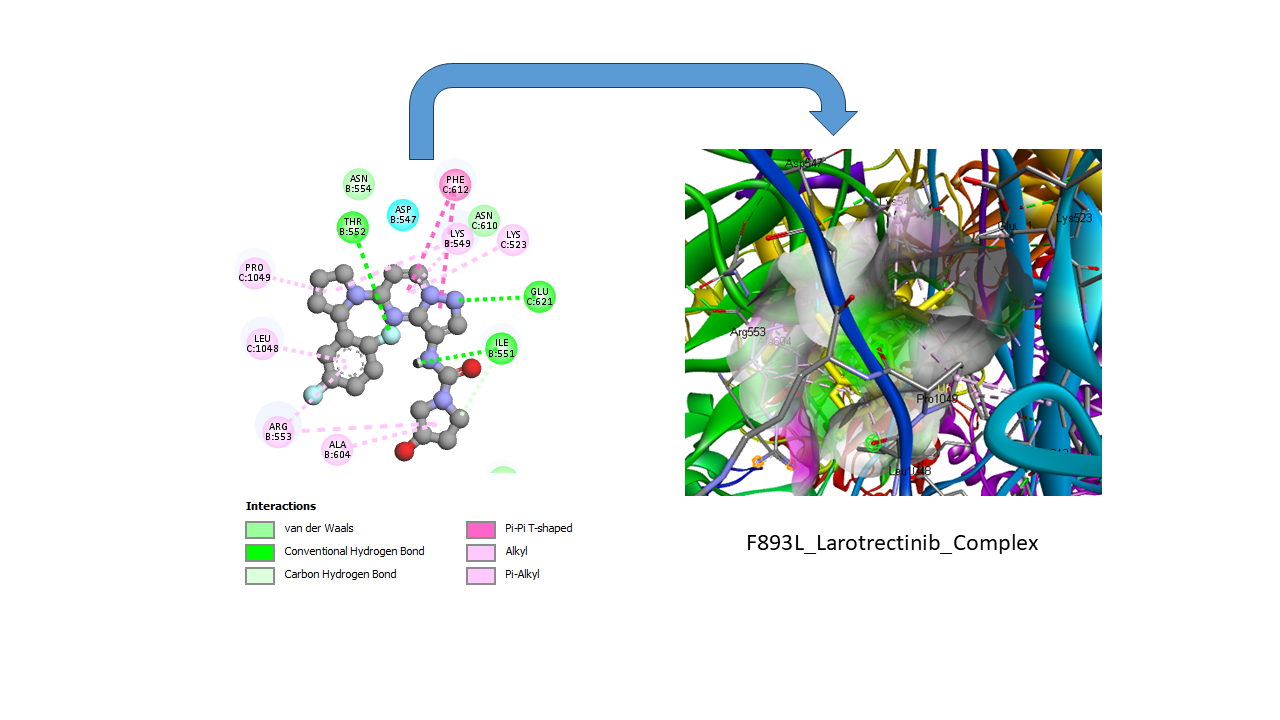


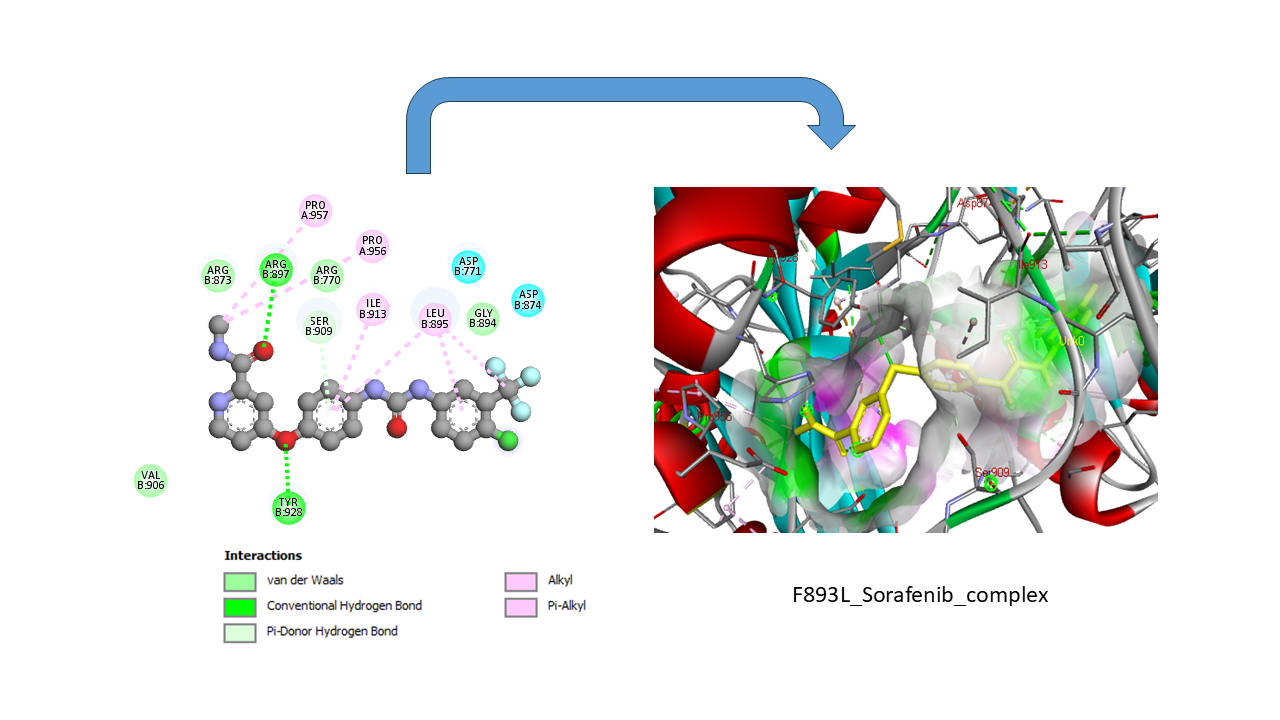


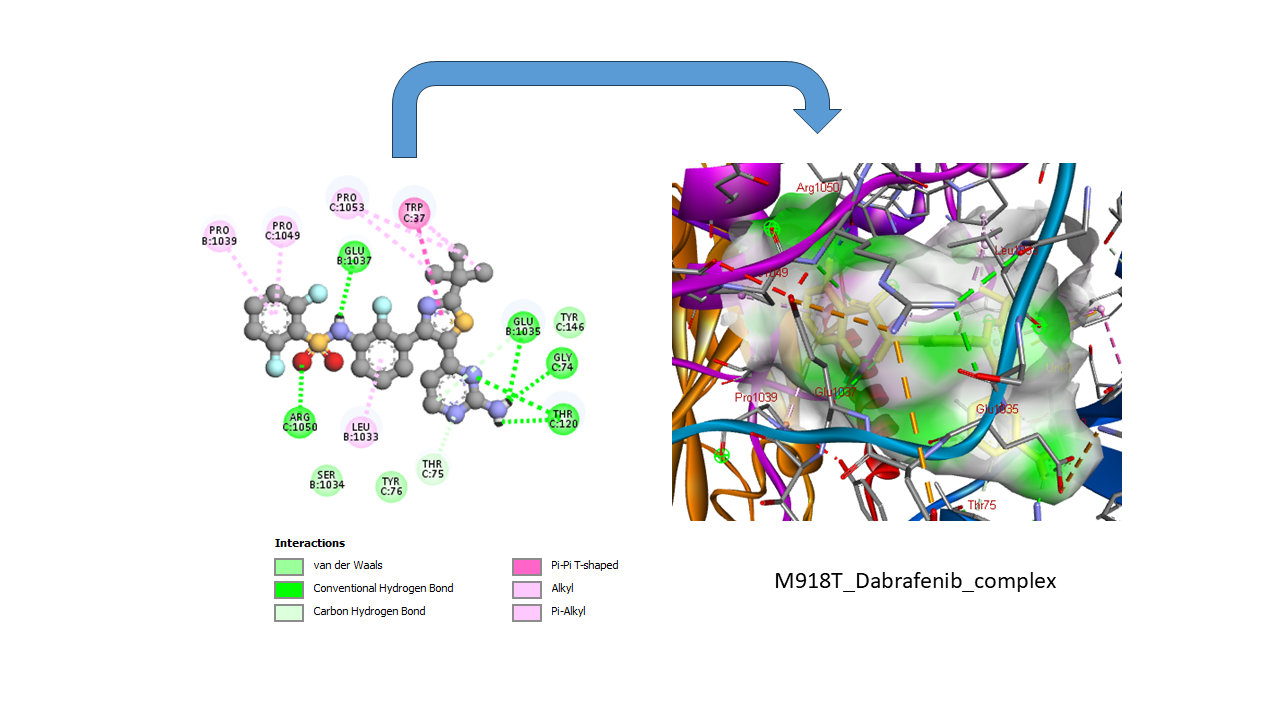


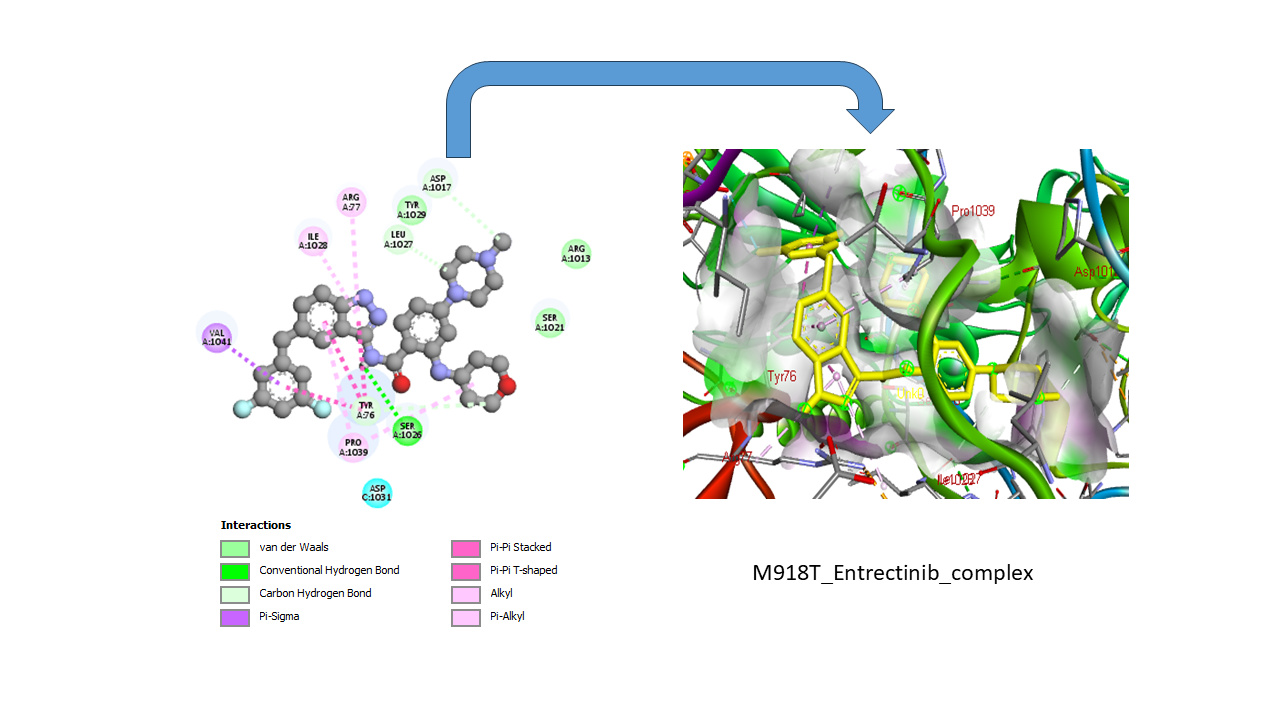


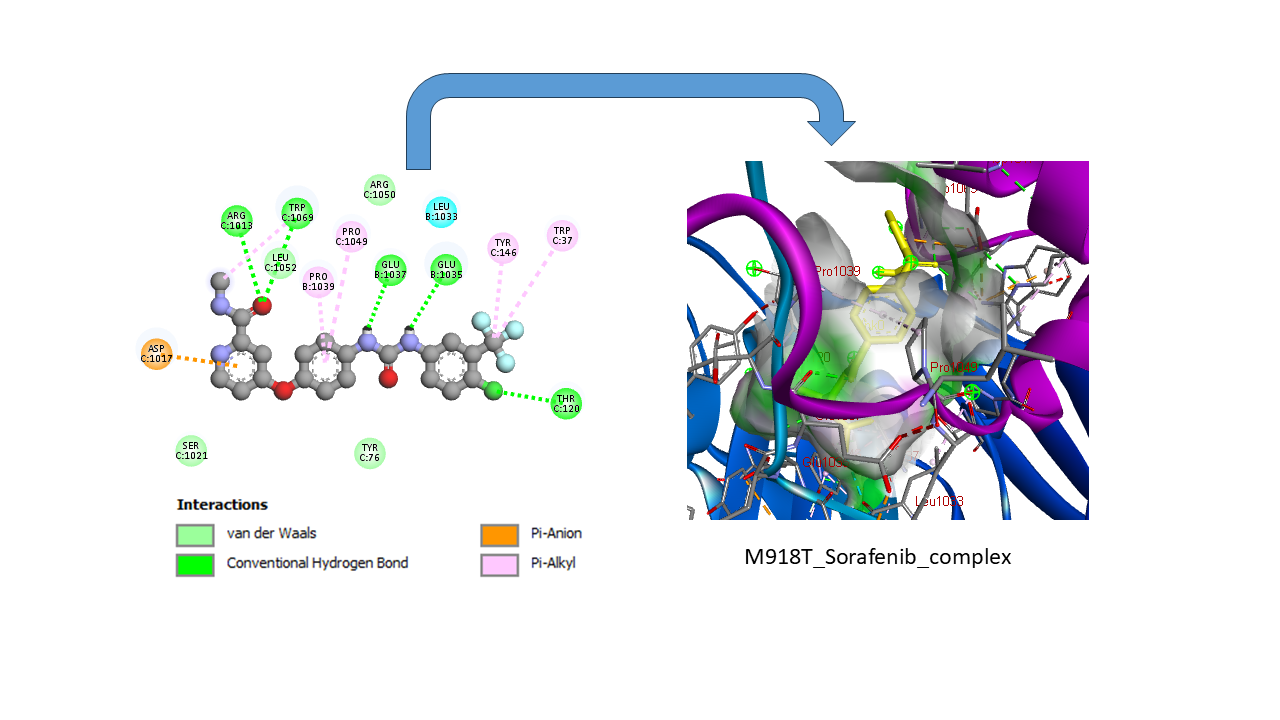


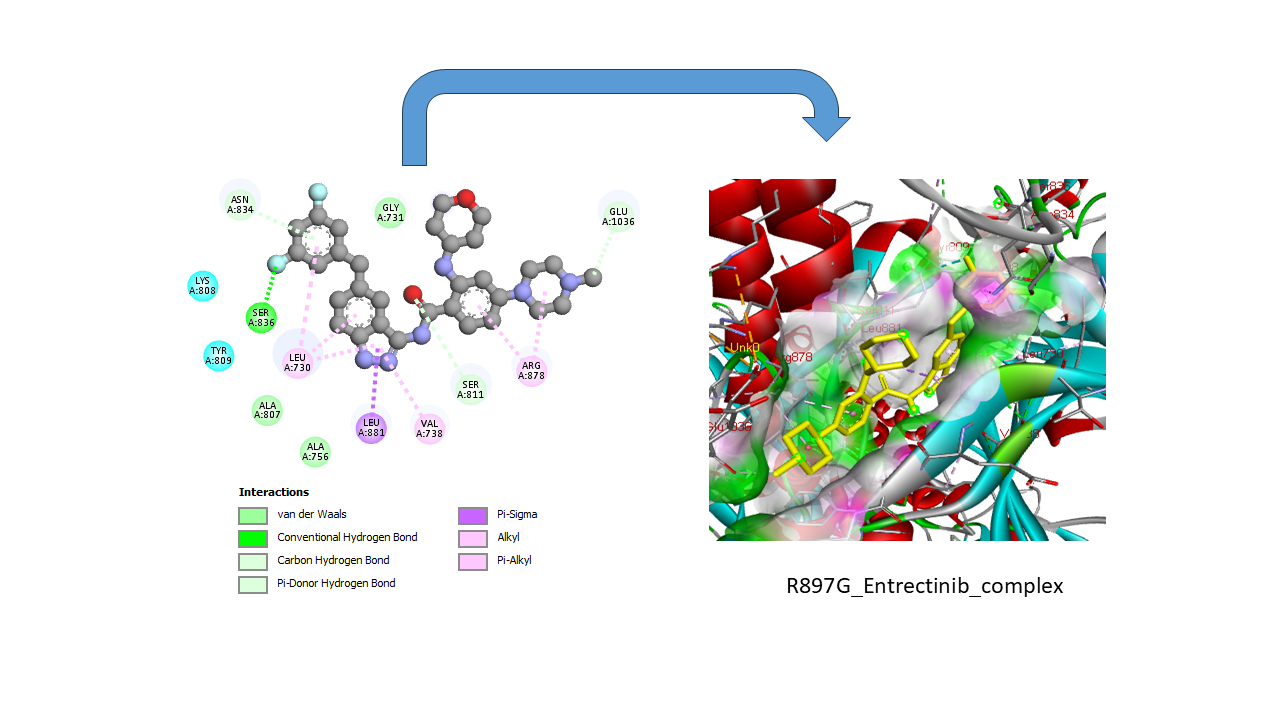


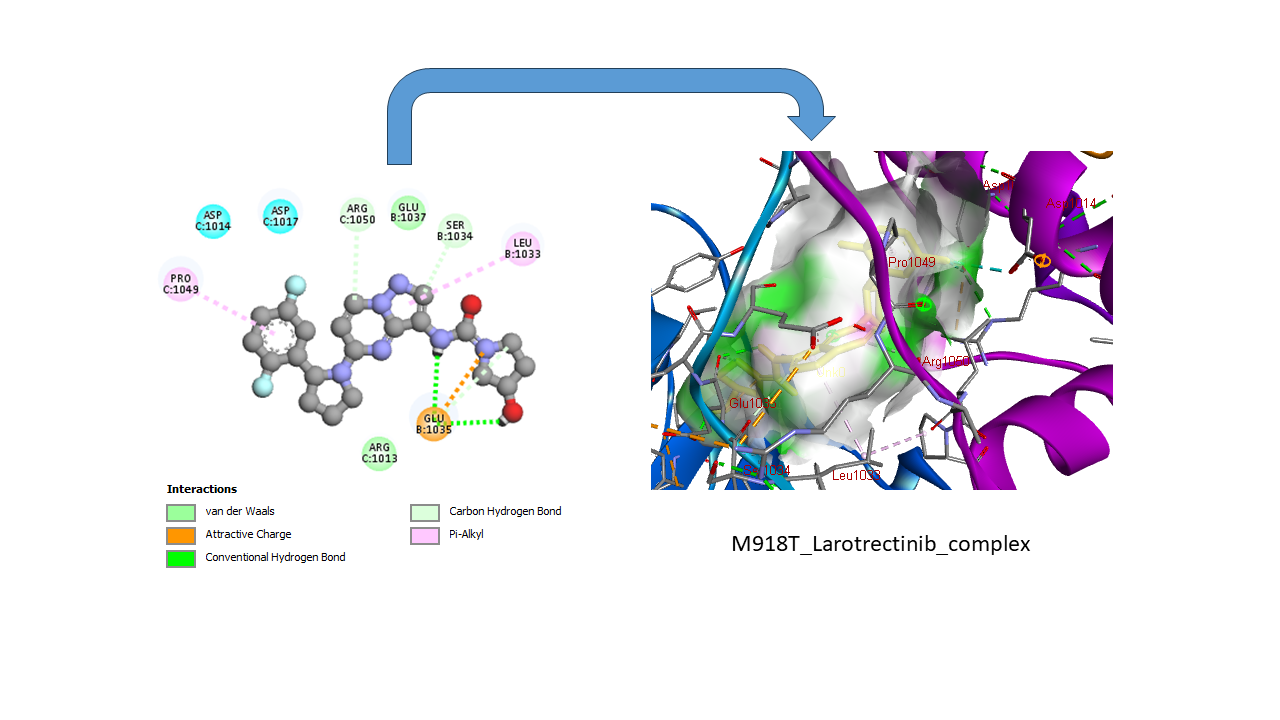


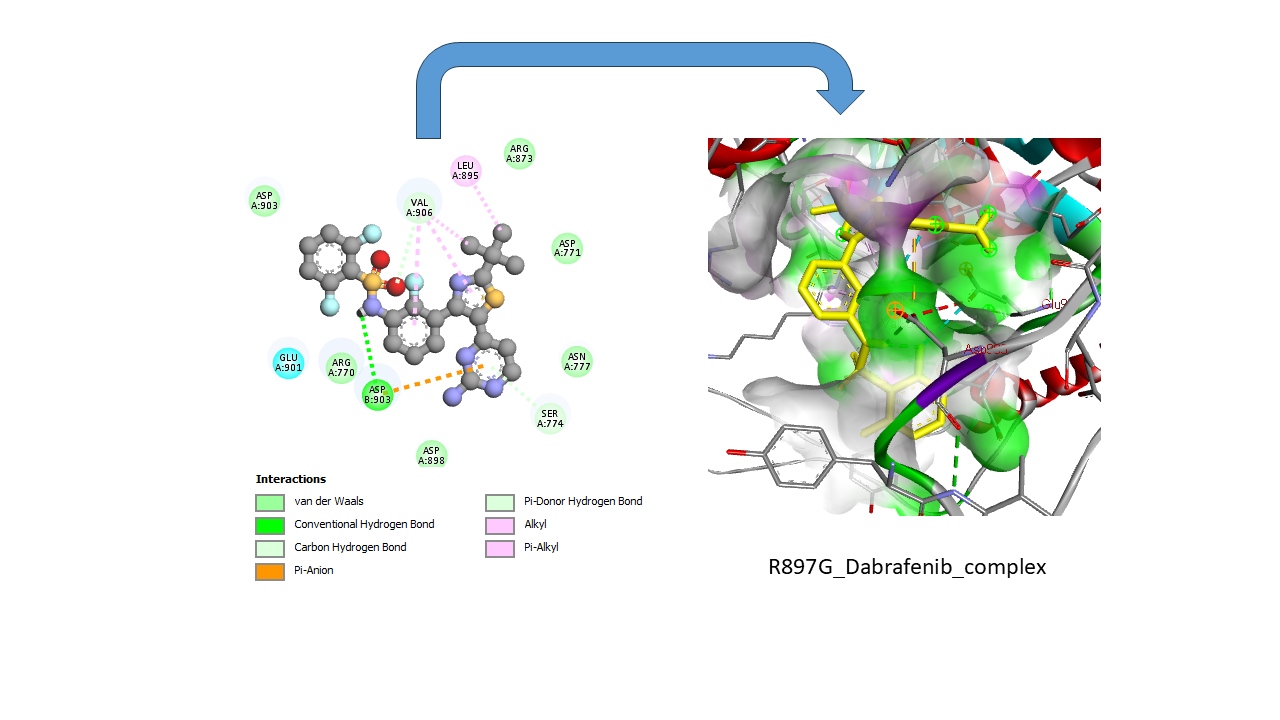


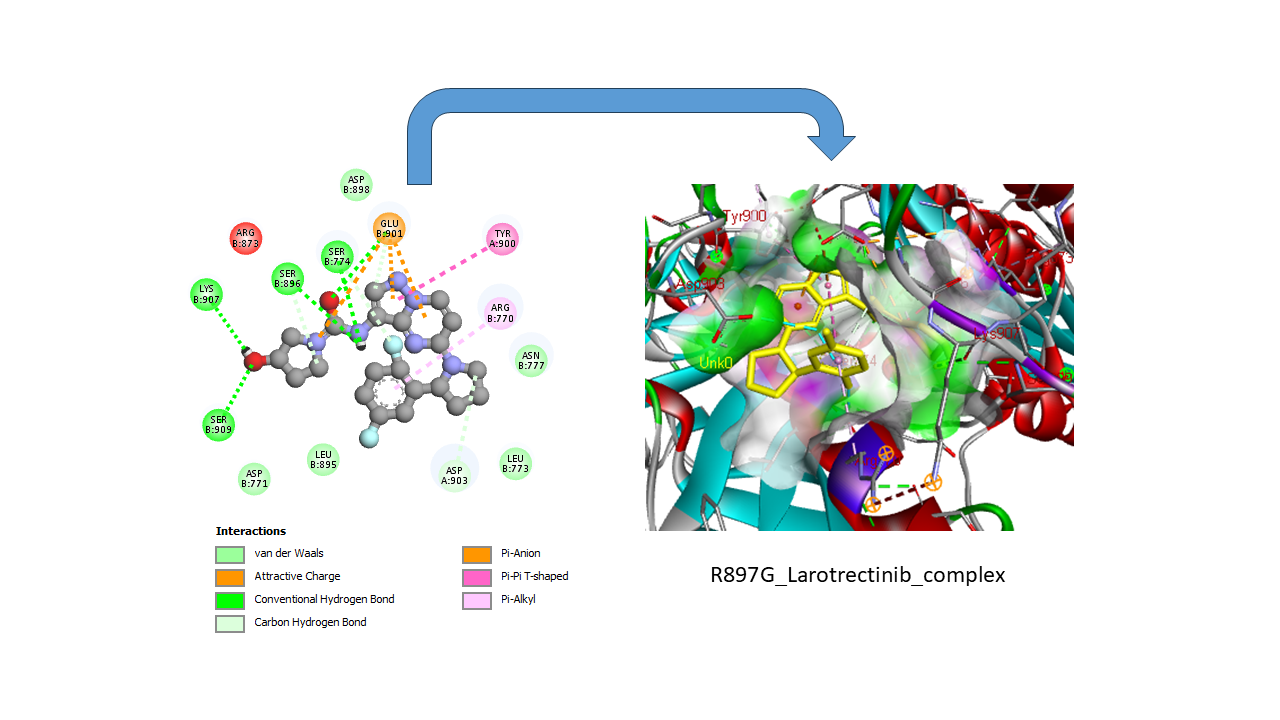

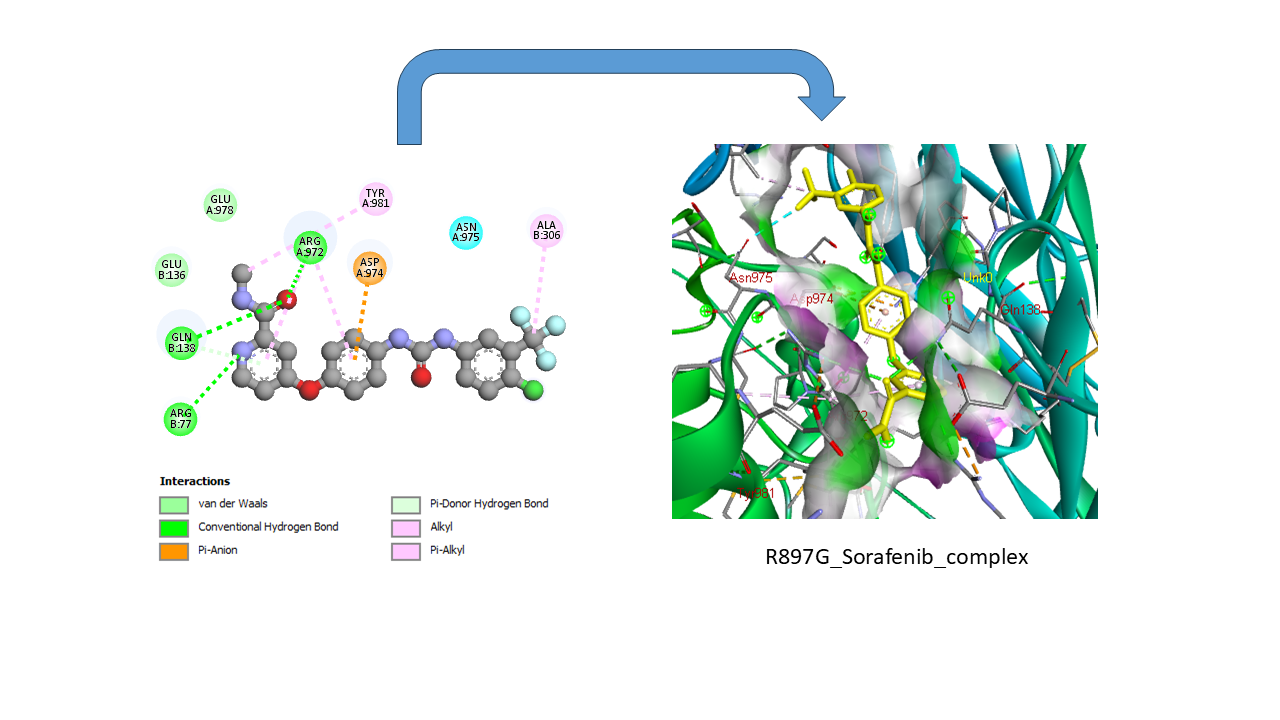

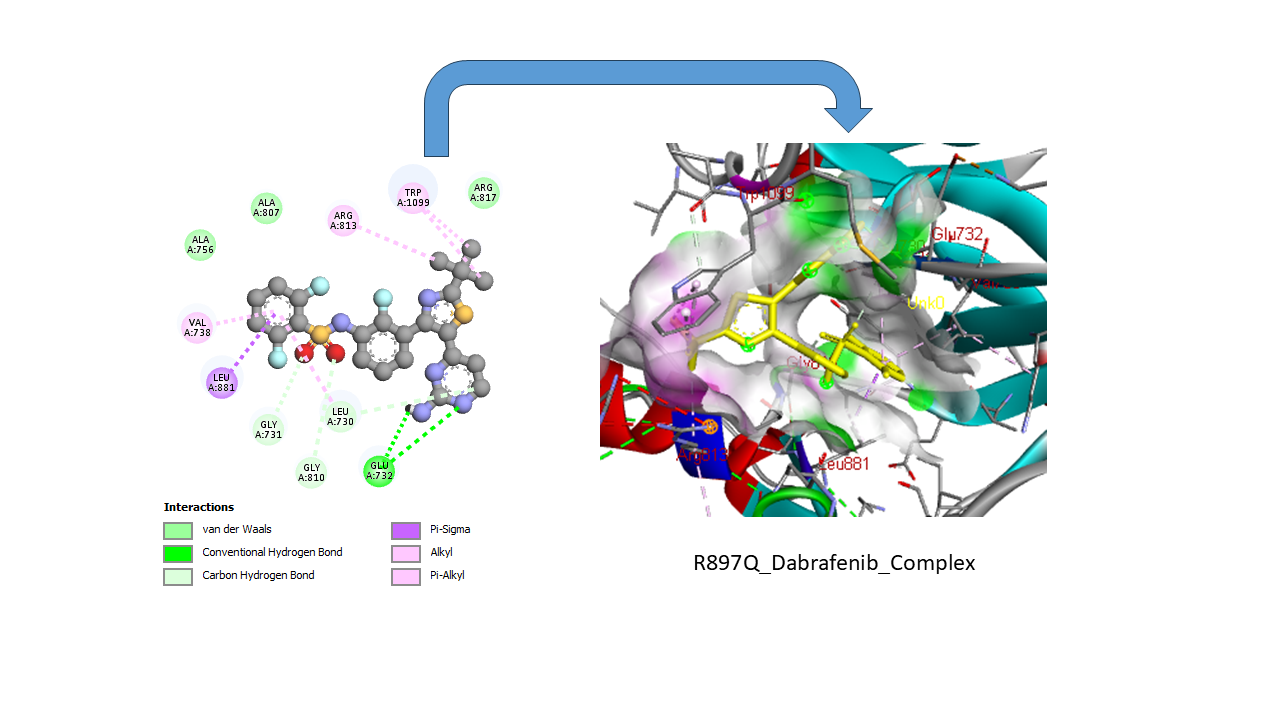

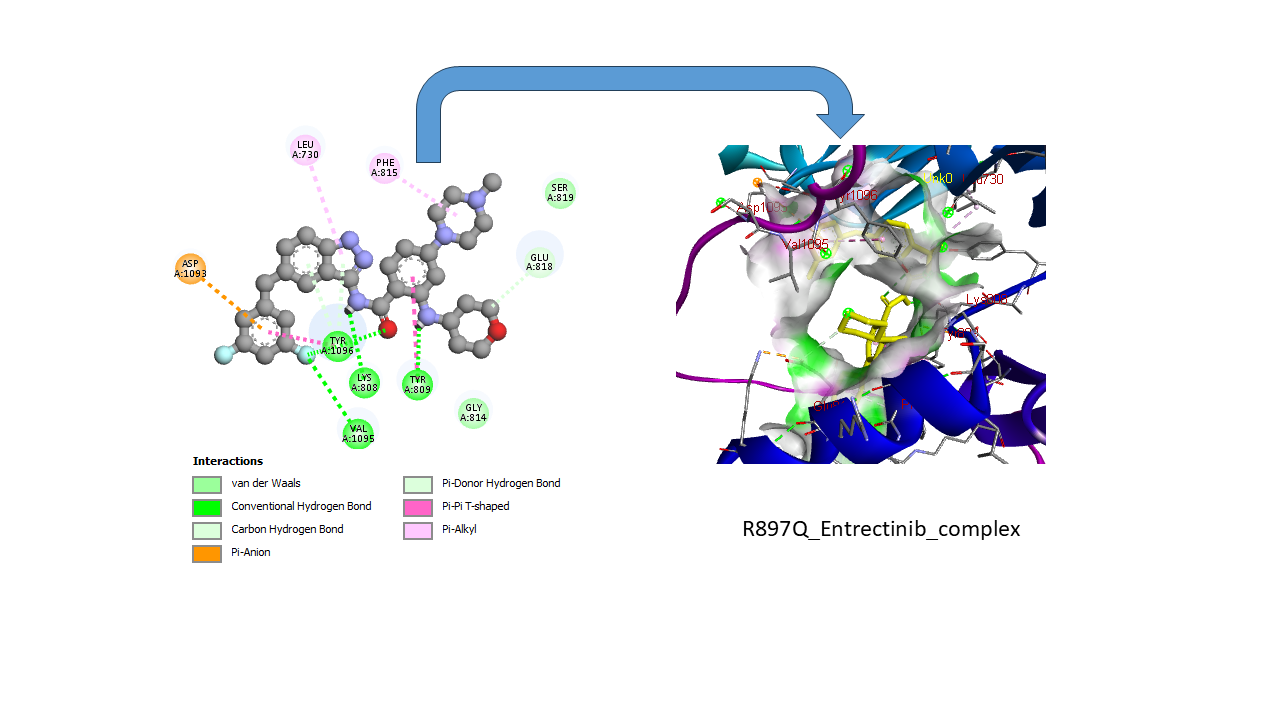

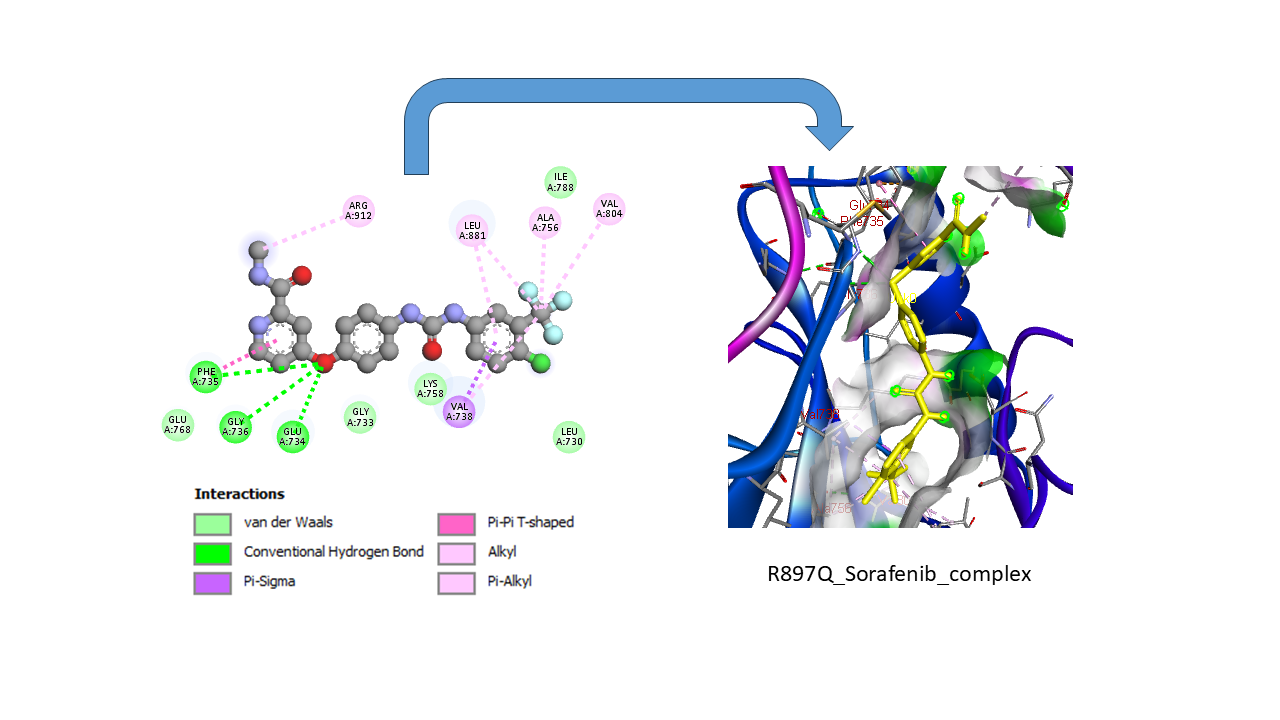


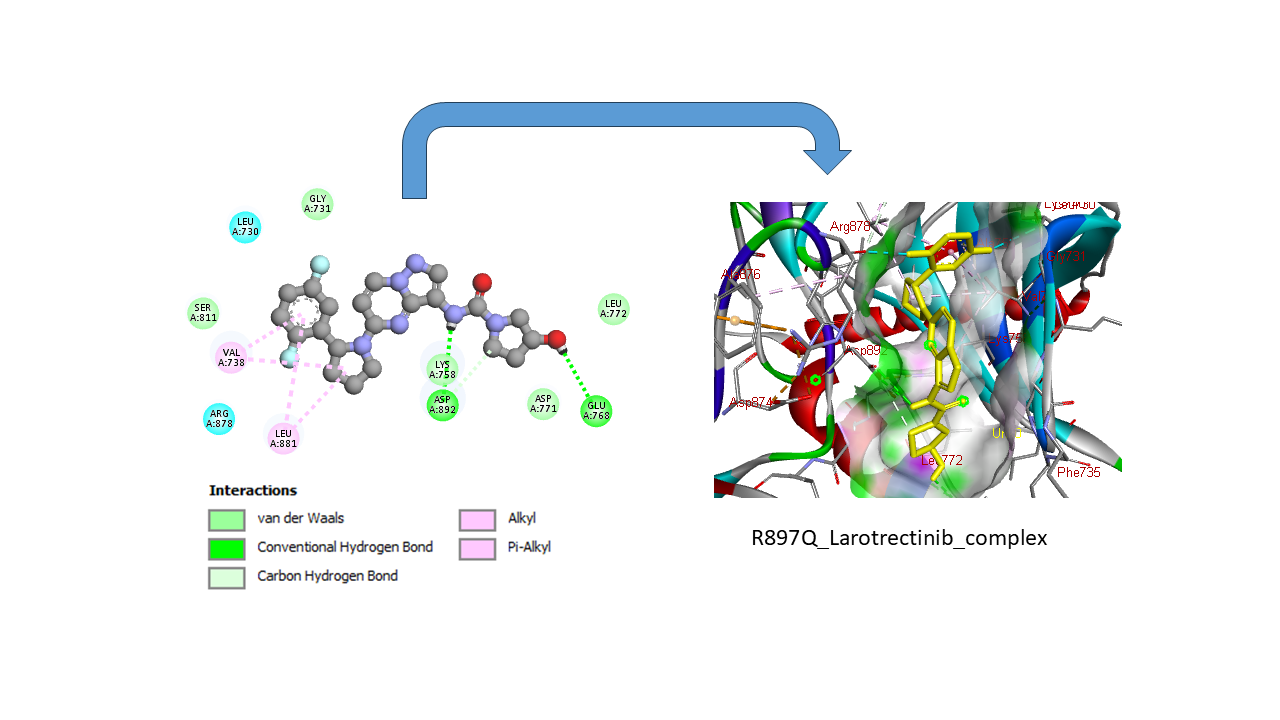


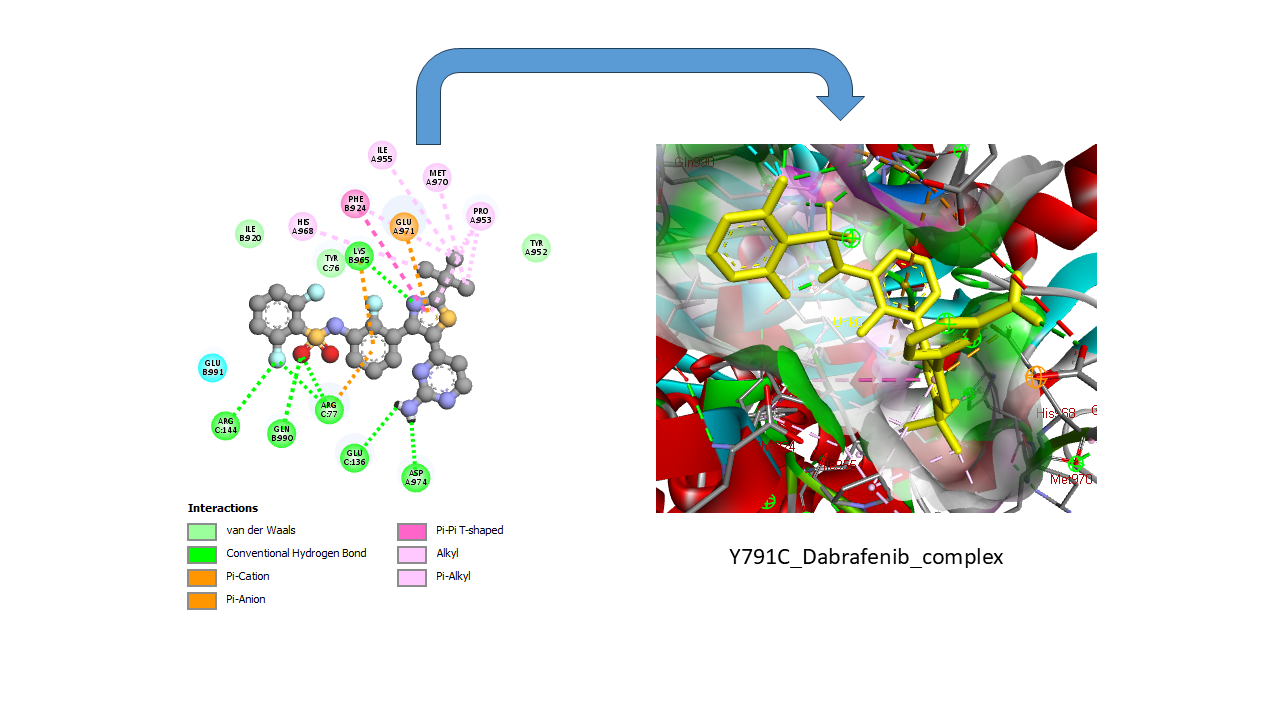

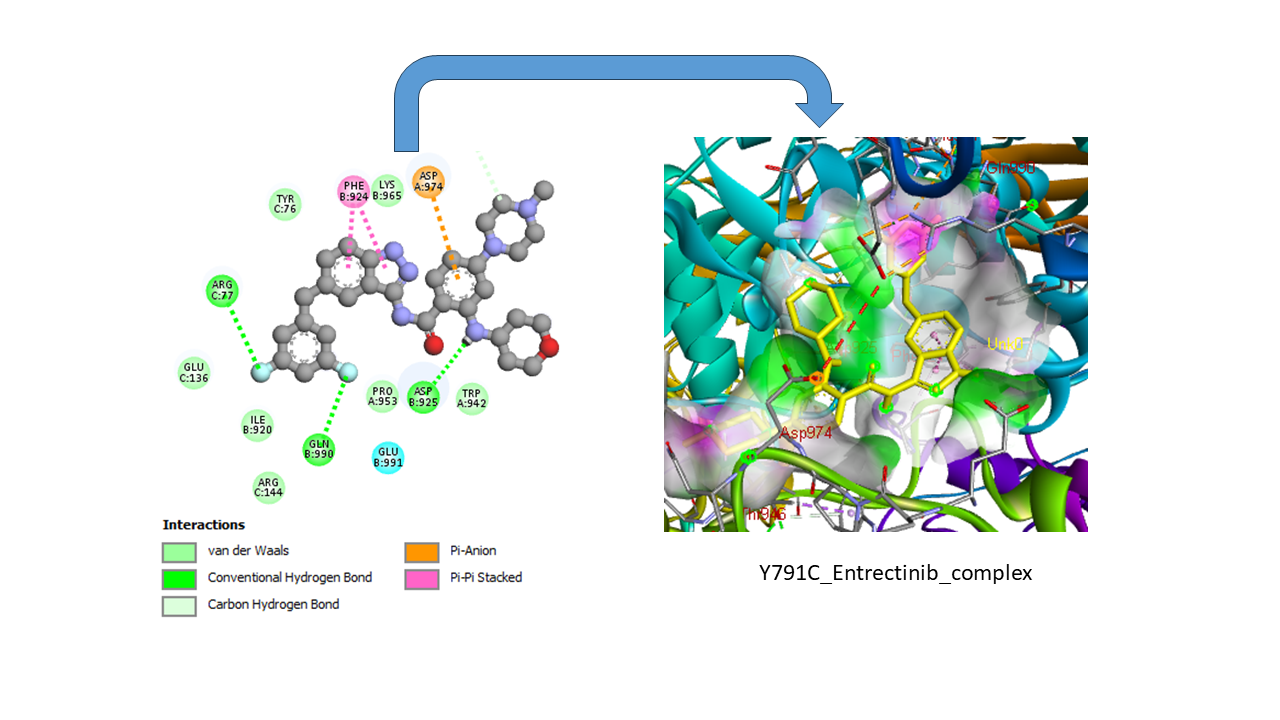

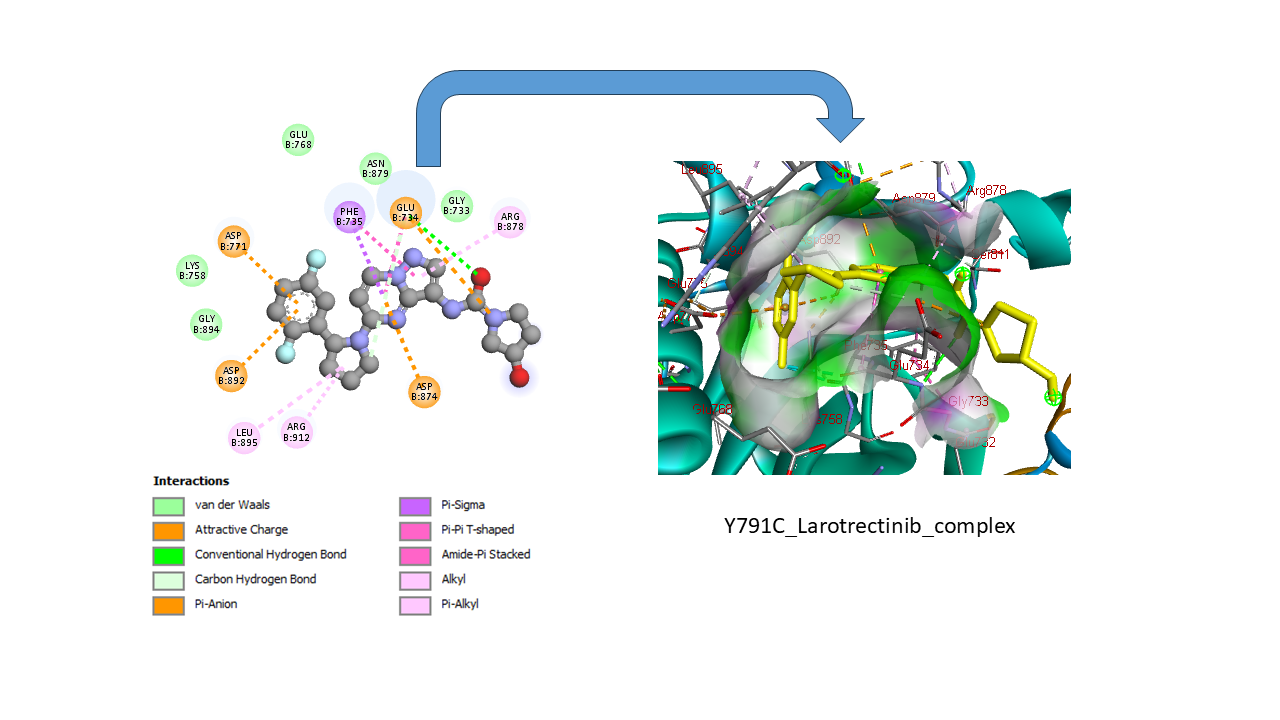


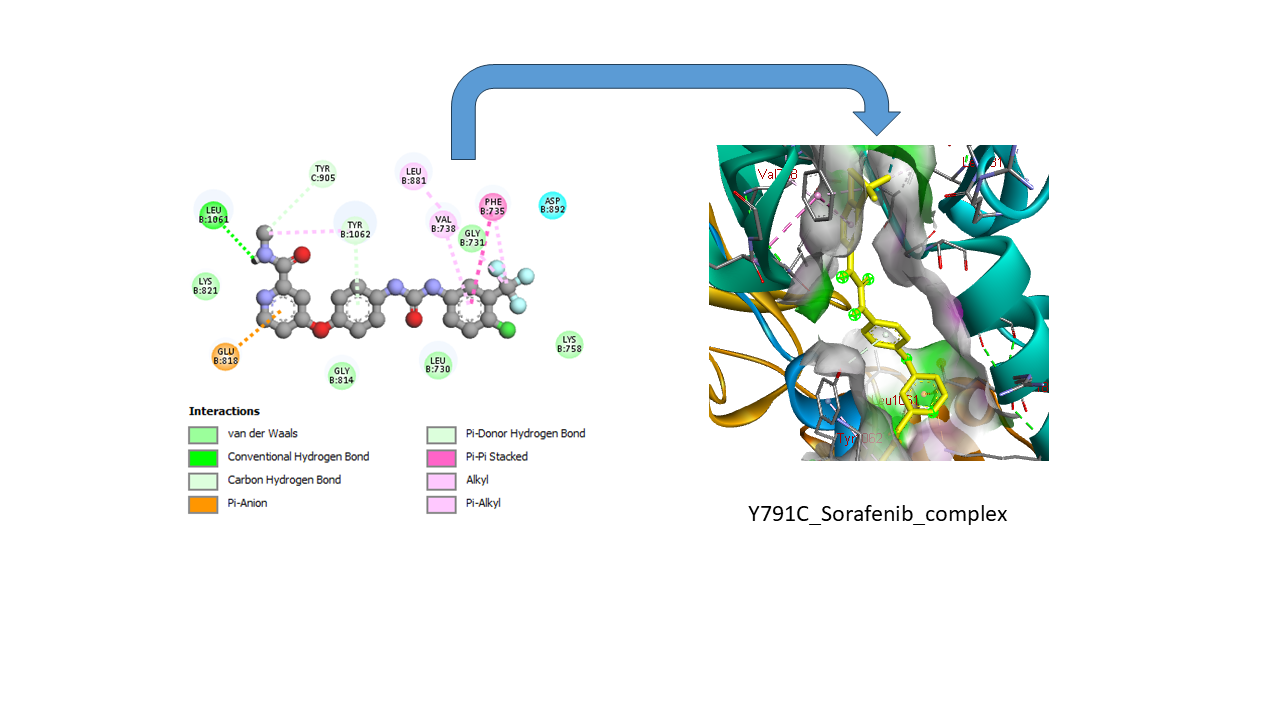


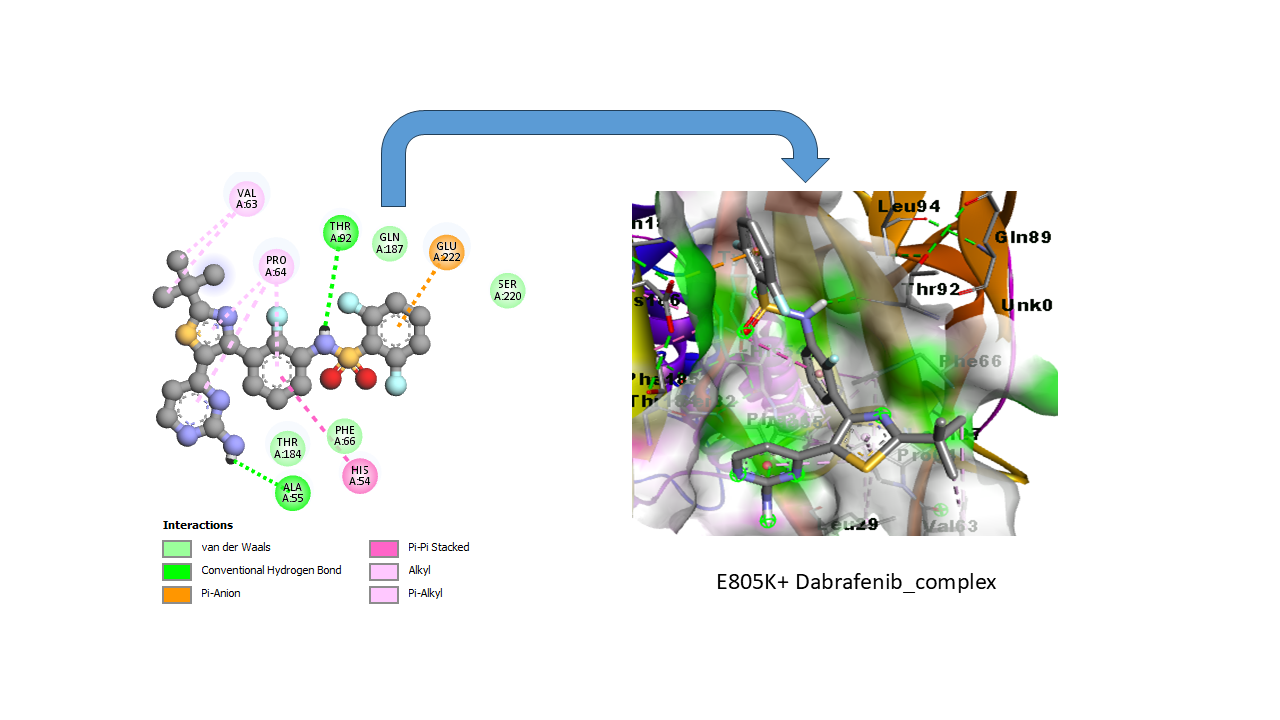


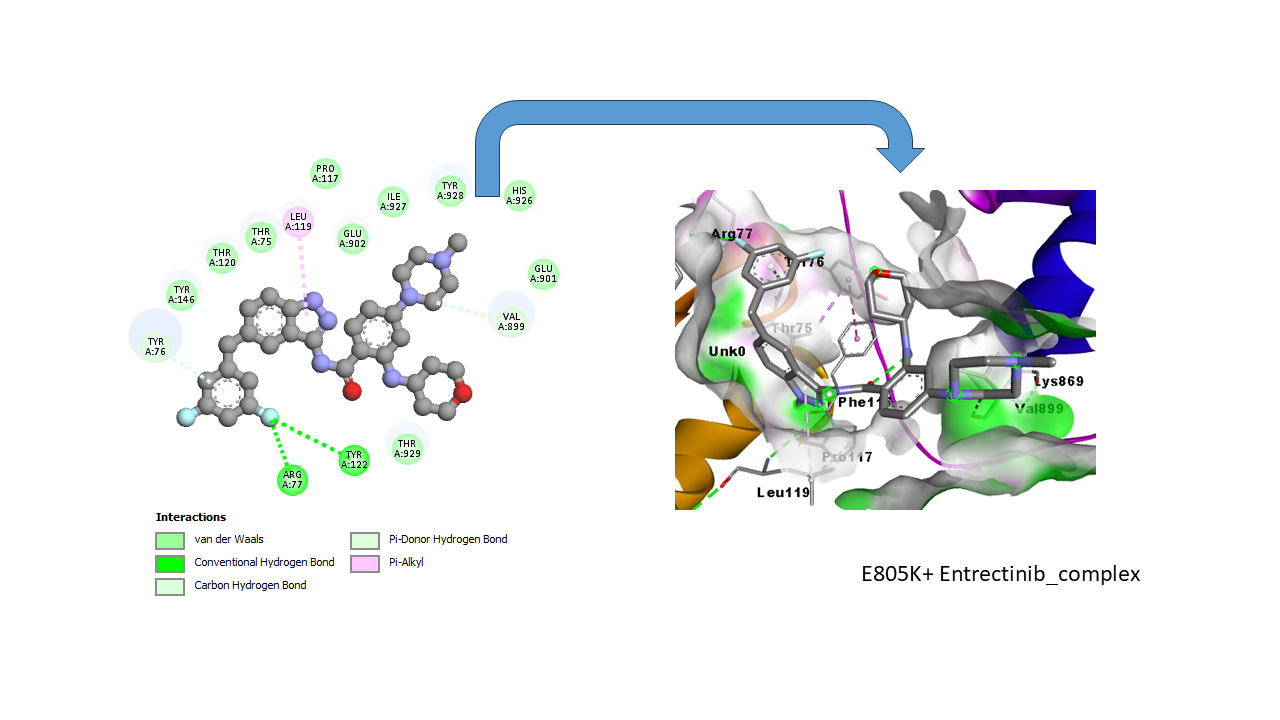


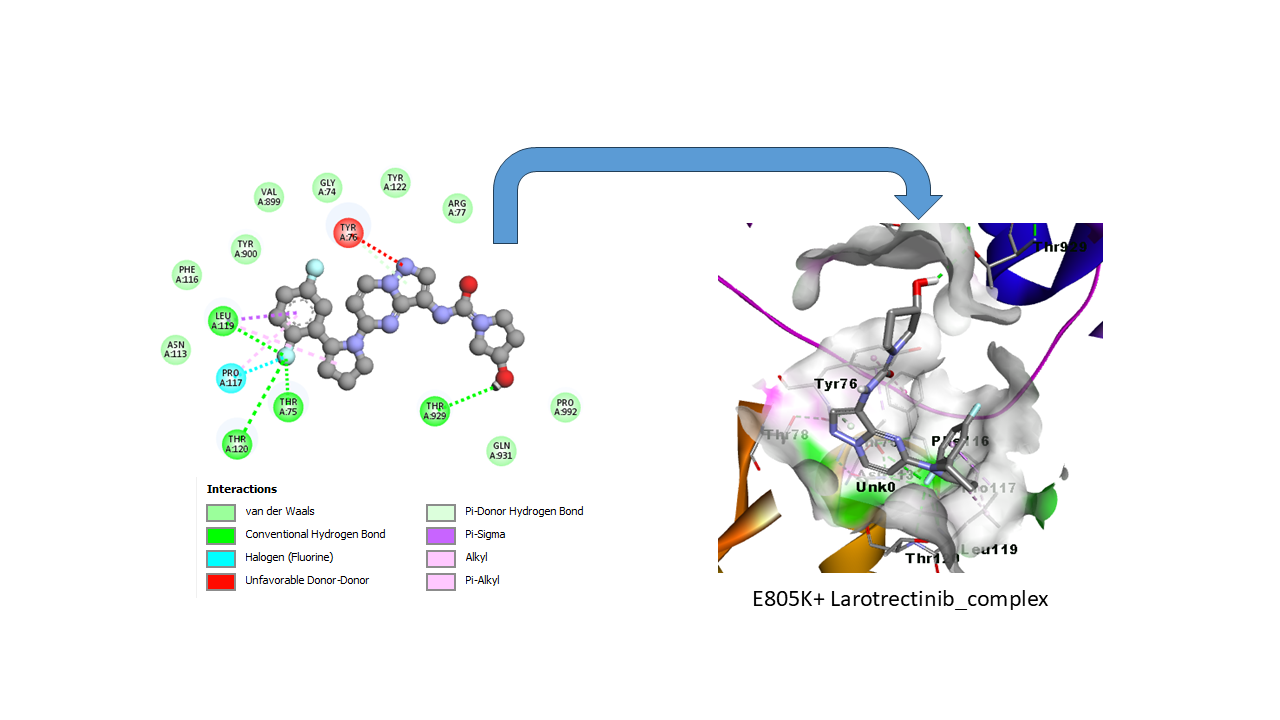


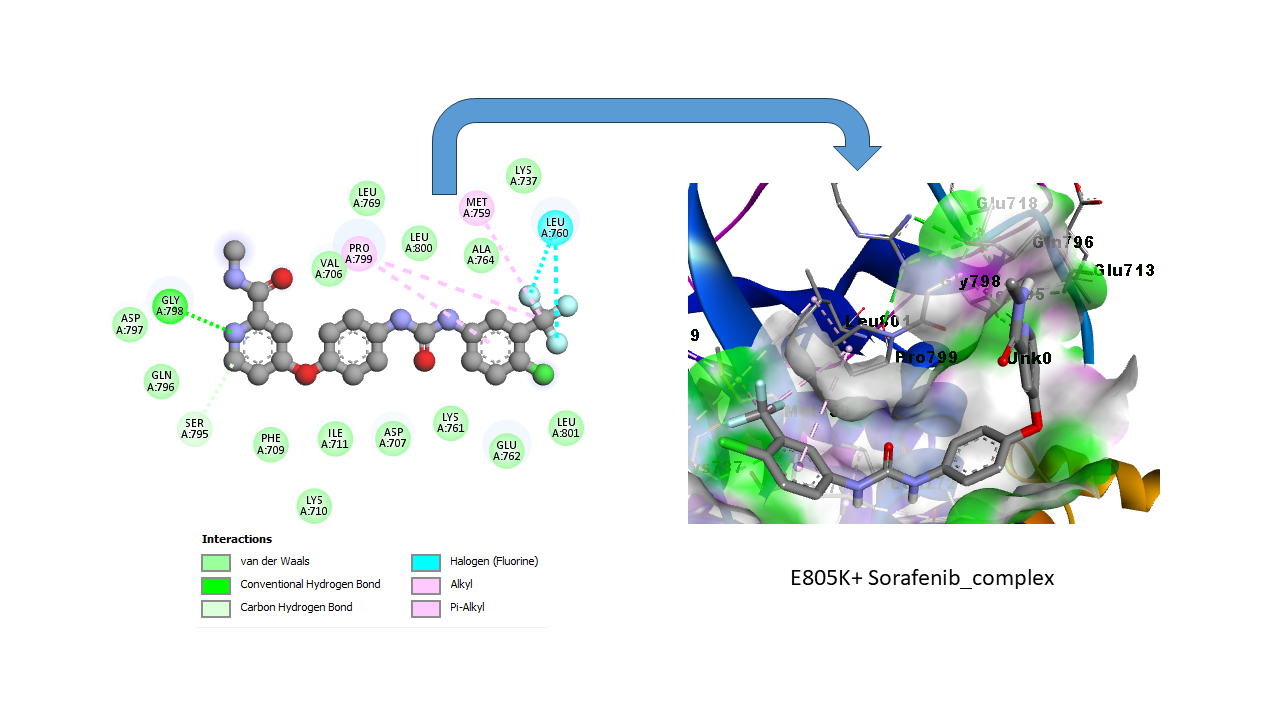


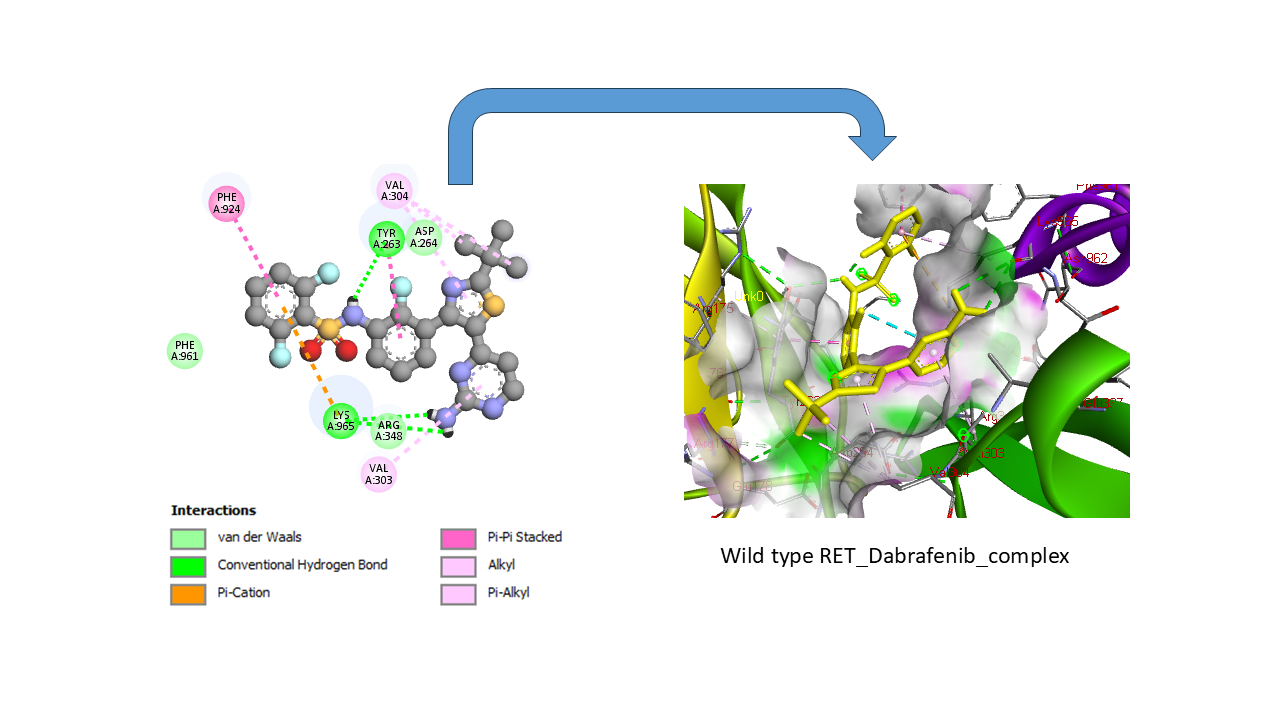


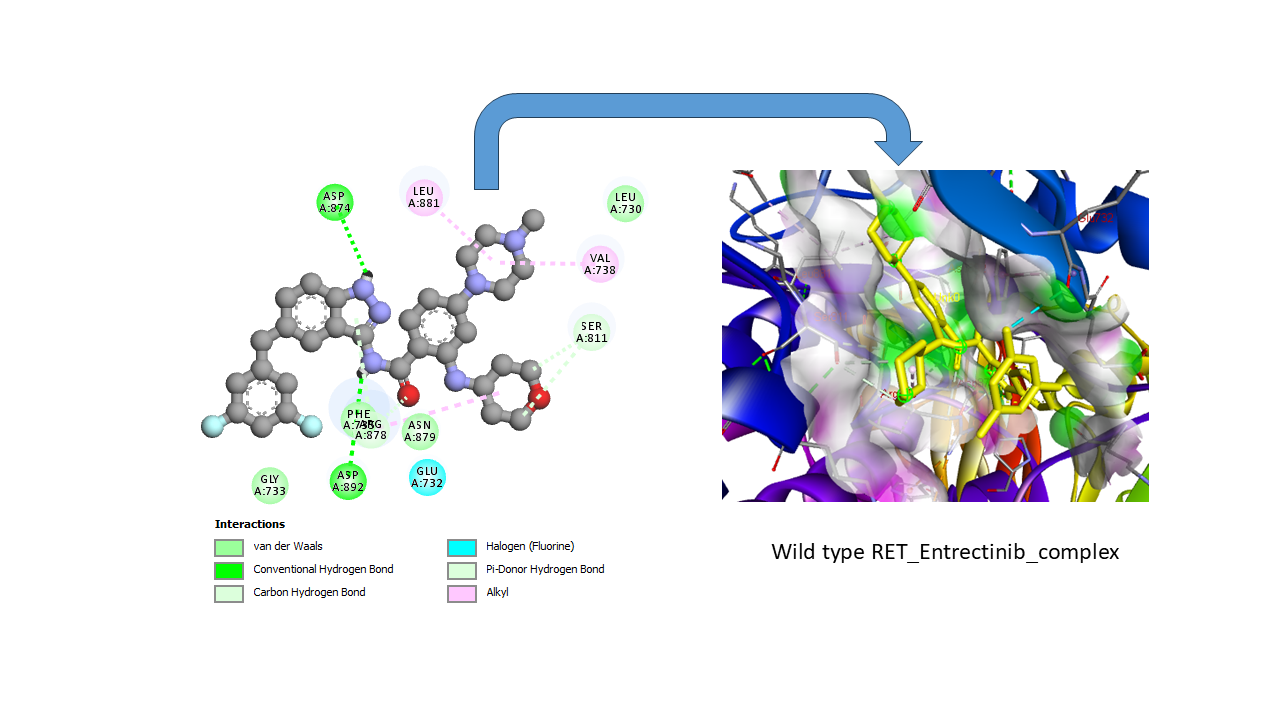


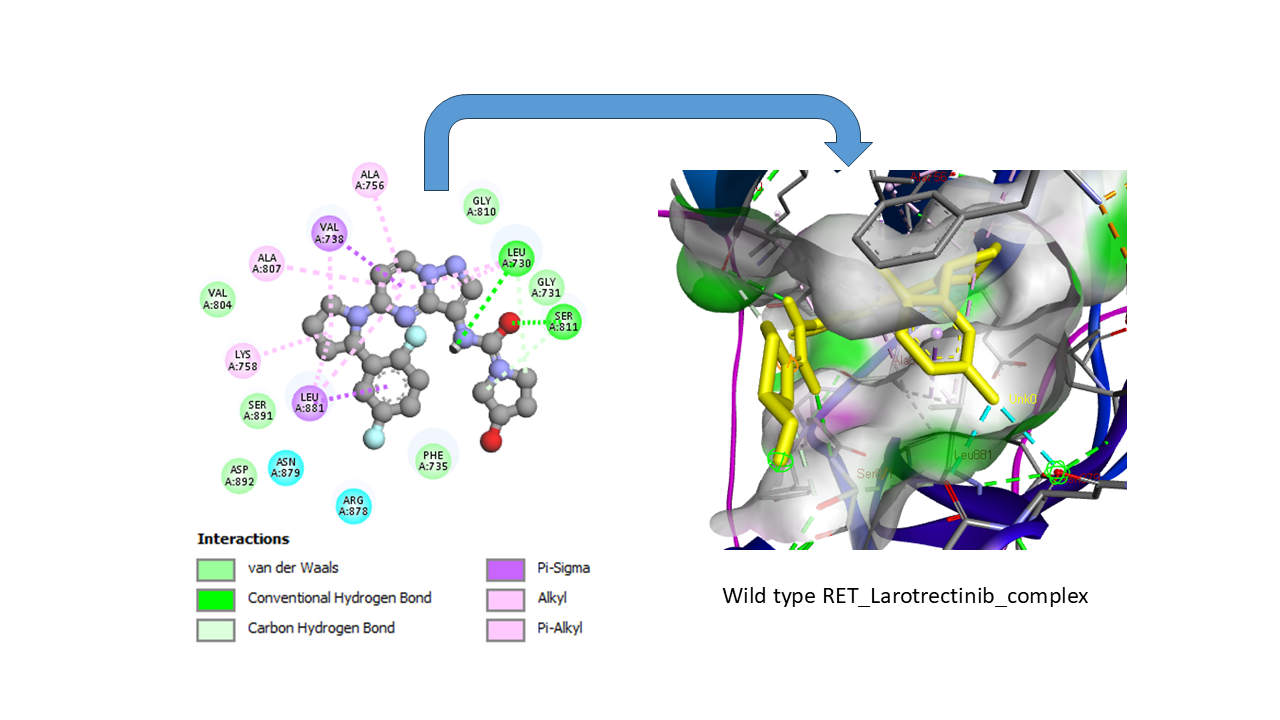

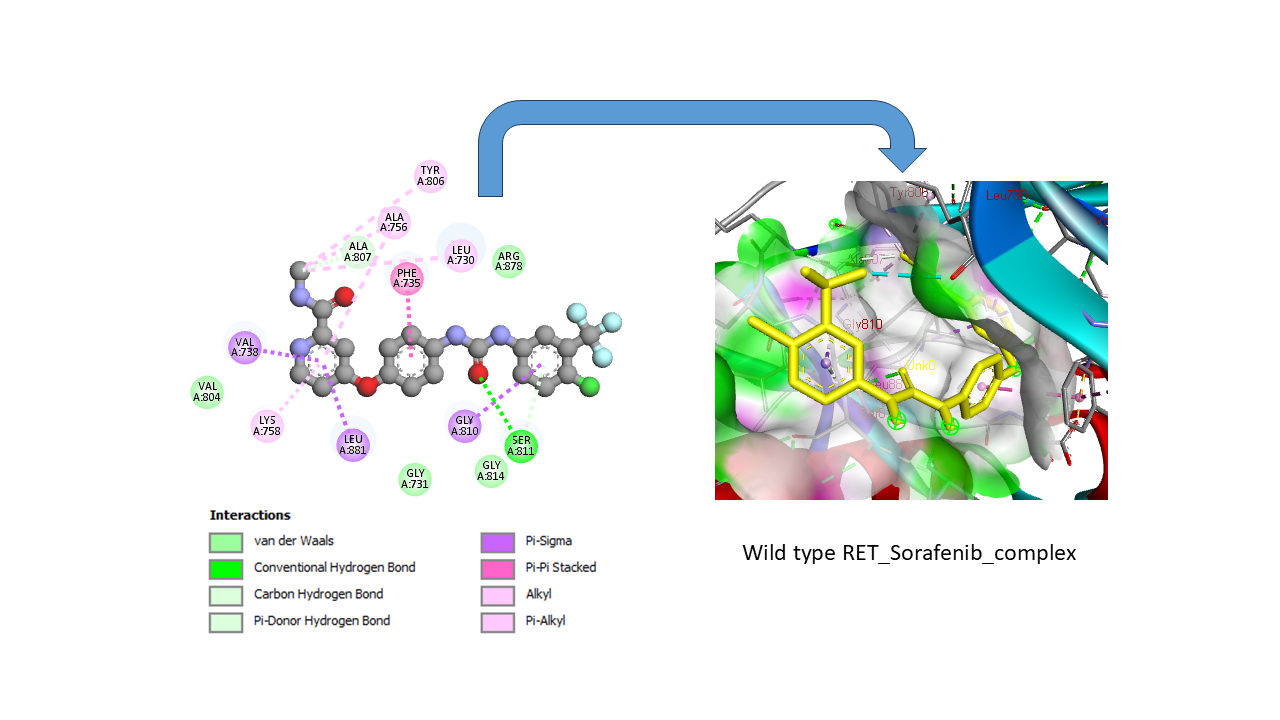


**FIGURE S5 |** Binding interaction analysis of 4 drug compounds (Dabrafenib, Entrectinib, Larotrectinib, and Sorafenib) against eight mutant structures (A756D, E734K, F893L, M918T, R897G, R897Q, Y791C, and E805K) and wild type RET protein. Left side indicates the 2D interaction and right side indicates the 3D interaction.

**3.11.3.3 Radius of Gyration Analysis**

The radius of gyration (Rg) analysis was conducted to evaluate the compactness and overall stability of RET mutants A756D, E734K, F893L, M918T, R897G, R897Q and Y791C during a 100 ns molecular dynamics simulation. Most variants maintained mean Rg values in the range of 5.0–5.4 Å, indicating retention of a compact conformation throughout the trajectory.

Specifically, A756D (**Figure S6A**), F893L (**Figure S6C**) and R897G (**Figure S6E**) exhibited highly stable profiles with minimal deviations, whereas M918T (**Figure S6D**) displayed an initially elevated Rg (6.0 Å) that rapidly converged to 5.2–5.4 Å. In addition, Y791C (**Figure S6G**) demonstrated a gradual decline from 5.4 to 4.8 Å, consistent with progressive compaction, while E734K (**Figure S6B**) and R897Q (**Figure S6F**) showed more pronounced fluctuations approaching 6.2 Å, indicating increased flexibility. In contrast, the RET wild type (**Figure S6H**) remained consistently stable with an Rg of 5.0–5.3 Å over the entire simulation, confirming the structural integrity of the native protein under identical conditions.

**3.11.3.4 Protein Ligand Interaction Analysis**

Molecular interaction analysis of the docked complexes revealed distinct binding patterns of the studied ligand with RET mutants A756D, E734K, F893L, M918T, R897G, R897Q and Y791C compared with the RET wild type (**Figure** **S7**).

In A756D (**Figure** **S7A**) and Y791C (**Figure** **S7G**), the ligand formed multiple hydrogen bonds with residues such as Arg, Asp and Glu together with several hydrophobic contacts, suggesting a highly stabilized binding mode. E734K (**Figure** **S7B**), M918T (**Figure** **S7D**) and R897G (**Figure** **S7E**) exhibited fewer polar interactions, with mainly Glu- and Ser-mediated hydrogen bonds, whereas F893L (**Figure** **S7C**) showed a distinct extended binding pose with additional van der Waals contacts along the hydrophobic pocket. R897Q (**Figure** **S7F**) presented a reduced interaction network dominated by non-polar contacts to Leu. In contrast, the RET wild type (**Figure S7H**) displayed the richest network of hydrogen bonds and hydrophobic contacts involving multiple residues within the ATP-binding cleft, indicating a more extensive and potentially stronger ligand engagement than most mutant complexes.

**
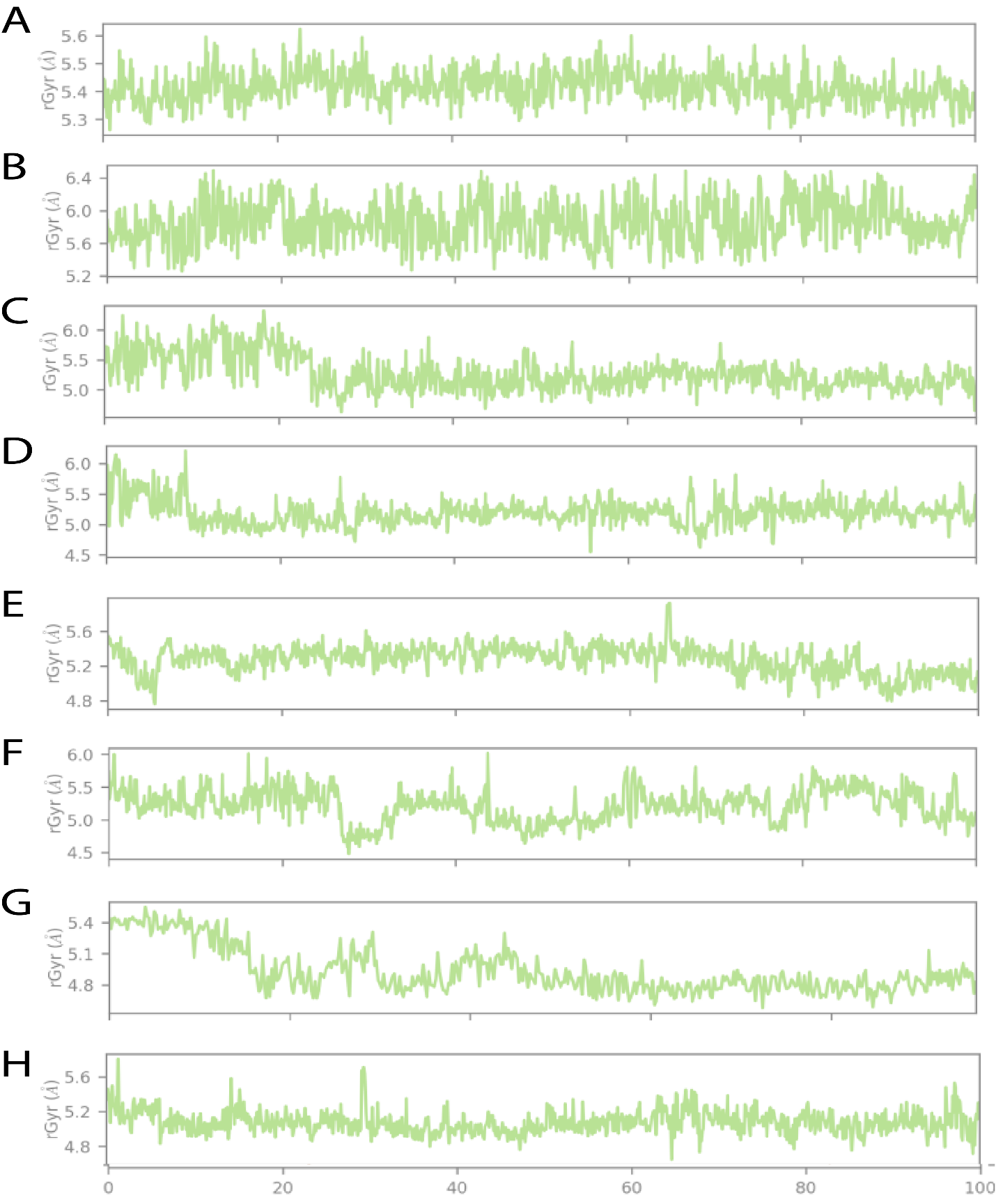
FIGURE S6 |** Radius of gyration (Rg) plots of RET mutants and wild type over a 100 ns molecular dynamics simulation. (A) A756D, (B) E734K, (C) F893L, (D) M918T, (E) R897G, (F) R897Q, (G) Y791C, and (H) RET wild type. The x-axis represents simulation time (ns), and the y-axis represents the radius of gyration (Å).

**
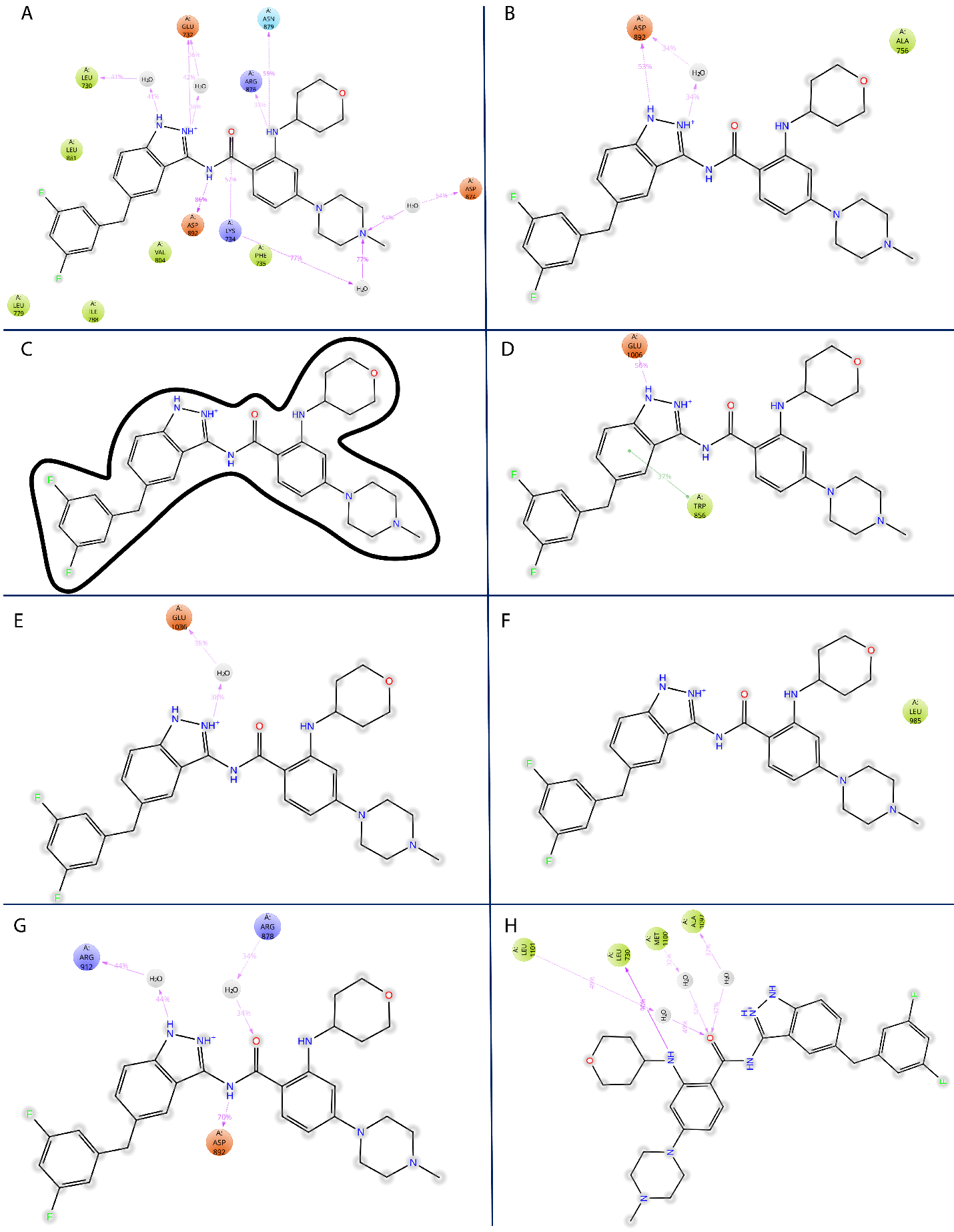
**

**FIGURE S7 |** Protein ligand interaction analysis via 2D diagram of RET mutants and wild type over a 100 ns molecular dynamics simulation. (**A**) A756D, (**B**) E734K, (**C**) F893L, (**D**) M918T, (**E**) R897G, (**F**) R897Q, (**G)** Y791C, and (**H**) RET wild type.
